# Bayesian Effect Size Ranking to Prioritise Genetic Risk Variants in Common Diseases for Follow‐Up Studies

**DOI:** 10.1002/gepi.22608

**Published:** 2025-01-03

**Authors:** Daniel J. M. Crouch, Jamie R. J. Inshaw, Catherine C. Robertson, Esther Ng, Jia‐Yuan Zhang, Wei‐Min Chen, Suna Onengut‐Gumuscu, Antony J. Cutler, Carlo Sidore, Francesco Cucca, Flemming Pociot, Patrick Concannon, Stephen S. Rich, John A. Todd

**Affiliations:** ^1^ JDRF/Wellcome Diabetes and Inflammation Laboratory, Nuffield Department of Medicine Centre for Human Genetics, NIHR Oxford Biomedical Research Centre University of Oxford Oxford UK; ^2^ Center for Public Health Genomics University of Virginia Charlottesville Virginia USA; ^3^ Nuffield Department of Orthopaedics Kennedy Institute of Rheumatology, Rheumatology and Musculoskeletal Sciences University of Oxford Oxford UK; ^4^ Department of Public Health Sciences University of Virginia Charlottesville Virginia USA; ^5^ Institute for Research in Genetics and Biomedicine (IRGB) Sardinia Italy; ^6^ Department of Pediatrics Herlev University Hospital Copenhagen Denmark; ^7^ Institute of Clinical Medicine, Faculty of Health and Medical Sciences University of Copenhagen Copenhagen Denmark; ^8^ Department of Clinical Research Steno Diabetes Center Copenhagen, Type 1 Diabetes Biology Gentofte Denmark; ^9^ Department of Pathology, Immunology, and Laboratory Medicine University of Florida Gainesville Florida USA; ^10^ Genetics Institute University of Florida Gainesville Florida USA

**Keywords:** effect size, empirical Bayes, false discovery rate, GWAS, significance

## Abstract

Biological datasets often consist of thousands or millions of variables, e.g. genetic variants or biomarkers, and when sample sizes are large it is common to find many associated with an outcome of interest, for example, disease risk in a GWAS, at high levels of statistical significance, but with very small effects. The False Discovery Rate (FDR) is used to identify effects of interest based on ranking variables according to their statistical significance. Here, we develop a complementary measure to the FDR, the priorityFDR, that ranks variables by a combination of effect size and significance, allowing further prioritisation among a set of variables that pass a significance or FDR threshold. Applying to the largest GWAS of type 1 diabetes to date (15,573 cases and 158,408 controls), we identified 26 independent genetic associations, including two newly‐reported loci, with qualitatively lower priorityFDRs than the remaining 175 signals. We detected putatively causal type 1 diabetes risk genes using Mendelian Randomisation, and found that these were located disproportionately close to low priorityFDR signals (*p* = 0.005), as were genes in the IL‐2 pathway (*p* = 0.003). Selecting variables on both effect size and significance can lead to improved prioritisation for mechanistic follow‐up studies from genetic and other large biological datasets.

## 1 Introduction

Biological processes are complex, stemming from combinations and interactions of many factors. Large, multivariate biological datasets reflect this complexity in that their analysis typically reveals large numbers of variables, such as genetic variants or biomarkers, to be associated with an outcome of interest, for example, disease risk. Variation in outcomes is therefore usually explained mostly by the combined small effects of many variables, which do not point to specific biological pathways or molecules. In genome‐wide association studies (GWAS), for example, genetic risk for common diseases is distributed across hundreds of loci, mostly with common alleles and small effect sizes (Ghoussaini et al. 2021). Interpretation of the biological effects of these variants, and establishment of which are causal, either statistically (fine‐mapping) or experimentally, requires considerable effort and the results may provide limited or non‐actionable mechanistic insight. In type 1 diabetes (T1D), for example, HLA, *INS* and several genes in the IL‐2 pathway (including *PTPN22* and *IL2RA)* have variants with alleles with relatively large effects on risk (Odds Ratios (ORs) > 1.2), and it is these loci that have yielded most biological insights so far (Dendrou et al. 2009; Downes et al. 2010; Ferreira et al. 2019; Garg et al. 2012; Smyth et al. 2008; Todd et al. 2016; Vafiadis et al. 1997), subsequently taken forward to translation and clinical trials (Marcovecchio et al. 2020). As sample sizes increase even further, for example from the availability of data from large biobanks, loci with *p* values that pass the established threshold for significance, *p* < 5 × 10^−8^, have either lower effect sizes or lower minor allele frequencies (MAFs), the latter of which are arguably preferable for follow‐up, but are less frequently found in practice (Crouch and Bodmer 2020).

A more permissive alternative to the typical genome‐wide significance threshold is the false discovery rate (FDR) (Benjamini and Yekutieli 2005; Brzyski et al. 2017; Chen et al. 2021; Stephens 2017), an estimate of the probability that associations beyond a specified P value threshold are false (e.g., at FDR = 0.01, 1% of associations are likely to be false). This yields more associations, but many may have small effect sizes, providing motivation to develop an alternative to the FDR that priorities variants based on both significance and effect size. A simple and popular approach is to threshold variables separately on both statistical significance (*p* value or FDR) and on the observed effect size estimate, but this does not provide quantitative evidence that the true effect size passes the threshold. Alternatively, it is possible to test whether variables pass a specified minimum effect size of interest (Cheng, Ramachandran, and Crawford 2020; McCarthy and Smyth 2009), but standard approaches are conservative as they test against the point null hypothesis that the effect equals the threshold, and furthermore do not prioritise among the variables passing the test. Bayesian approaches are best equipped to circumvent these issues by providing posterior distributions of effect sizes, but typically require some a priori assumptions about the distribution of effects. Here, we developed an empirical Bayesian method to estimate the ranks of the effect sizes in a data set, without needing to assume a specific form of prior distribution. Since the method both utilises the FDR and is complementary to it, we termed it the priorityFDR. We assessed the performance of the priorityFDR using simulated data, and compared its performance at rank estimation against a similar method, EBrank (Ferguson and Chang 2020), which models the prior as a mixture of normal distributions. Our approach is preferable to performing a simple ranking of the Bayesian estimates of effect sizes across the variables in the data set, as this does not account for the overlaps in their posterior distributions, which may be substantial.

We conducted a GWAS meta‐analysis of T1D, the largest to date (15,573 cases and 155,235 controls) and assessed how priorityFDR can be used to target the most biologically informative associations. To achieve this, we (a) tested whether the priorityFDR is able to objectively prioritise variants associated with the established T1D‐associated IL‐2 causal pathway, and (b) tested whether the method prioritises variants close to genes with putatively causal effects on T1D, using Mendelian Randomisation incorporating blood eQTL data from eQTLgen (Võsa et al. 2021).

In addition to prioritising many known associations, we found two highly prioritised signals not detected in previous GWAS reports, and 21 others at a second tier of prioritisation, compared to four previously unreported loci and 54 known signals using the genome‐wide significance threshold of *p* ≤ 5 × 10^−8^.

## 2 Results

### 2.1 Defining the PriorityFDR

The priorityFDR is defined, for a given variable, as the overall probability that either (a) the variable is a false positive association or (b) the variable is a true positive but randomly choosing variables from the distribution of true associations produces a larger effect size. This is equivalent to the proportion of non‐null variables with effect sizes that exceed the variable in question. It is useful to define the measure in this way because many users would want to first filter out variables that appear to be non‐null by applying a *p* value or FDR threshold, and in these cases would be less interested in the overall ranking.

Informally (see Supporting Information S1: Appendix S1 for formal definitions), the priorityFDR for variable *i* is:

(1) $${\text{priorityFDR}}_{i}={\text{FDR}}_{i}+{\text{effect priority}}_{i}\times \left(1-{\text{FDR}}_{i}\right),$$

where,

$${\text{effect priority}}_{i}=\text{Pr}\left(\text{Effect size of random non‐null variable}>\text{Effect size of variable}\;i\;\text{| variable}\;i\;\text{is non‐null}\right)$$

(2) ≈{Proportion of non‐null effect sizes>Effect size of variable i | variable i is non‐null},

and as

(3) {Proportion of non‐null effect sizes>Effect size of variable i | variableiis null}=1,

(4) $${\text{priorityFDR}}_{i}\approx \left\{\text{Proportion of non‐null effect sizes}>\text{Effect size of variable}\;i\right\}.$$

The priorityFDR is lower bounded by the FDR, so that all priorityFDRs below 1%, for example, will also have FDR ≤ 1%. Therefore, it applies further stringency to a set of associations passing the same FDR threshold. Variables with low effect priority will usually also have low FDRs, as if their effect sizes can be distinguished accurately from those of other variables (low effect priority) this implies that they can also be distinguished from zero (low FDR). However, variables with low FDR may not have low (or particularly low) effect priority, as they can have effect estimates lying close to zero compared with other variables, while having tight standard errors. For a variable to produce a low priorityFDR, it is necessary for it to have both high statistical power to reject the null hypothesis of no effect plus an effect size that is relatively large compared to other variables in the data set. Ability to detect whether its effect size is larger than that of the other variables will be lower than than the ability to detect whether it exceeds zero. Thus, even when the true effect size is high, low statistical power due to a small ratio of said effect size over the standard error (as is often the case for rare genetic variants) will translate to high priorityFDRs, just as it translates to high FDRs.

We developed an empirical Bayesian method for estimating the priorityFDR without the need to explicitly model prior distributions of variables' effects (see Supporting Information S1: Appendix S1 for full details). Our approach is to model the distribution (likelihood) of the data only, without modelling the prior, by fitting a flexible polynomial‐based function (henceforth a “polynomial”) to the observed histogram of effects (on the Z‐score scale), along with a point‐null distribution, providing estimated null and non‐null mixture components. We then sample from the estimated non‐null distribution, fitting two polynomial mixtures to this sampled histogram, corresponding to variables with negative and positive true effects, allowing for computation of Bayesian probabilities of the true effect signs. This is achieved by constraining the two polynomial likelihood models so that they contain variables with only positive and only negative true effects (see Supporting Information S1: Appendix S1). We then apply the same approach to modelling the distributions of randomly sampled effect differences between pairs of variables, rather than effects of individual variables, allowing computation of Bayesian sign probabilities for the true effect difference between a given pair. The Bayesian effect sign and effect difference sign probabilities together allow Bayesian computations of effect size order (i.e., whether one variable has a true effect more distant from zero than the other), and averaging over samples of many such pairwise differences, keeping the variable of interest *i* as the first member of the pair, allows estimation of the effect priority in Equation 1. One advantage of our approach is that there is no need to specify explicit forms for the prior distributions. Even though very flexible priors could be specified, for example using a large number of mixtures of normal distributions as in EBrank (Ferguson and Chang 2020), it is possible that these are more difficult to fit. Secondly, as we are interested in the rankings among non‐null variables, it may be important to ensure that the proportion of variables that are non‐null is estimated accurately, which may not be the case if the model for the prior is not able to well‐approximate the true prior. We aimed to avoid this limitation by capturing true null variables using a standard normal distribution with mean zero, mixed with a flexible polynomial‐based model for the non‐null distribution.

Implemented in R (as the priorsplitteR package), using multiple threads, the method typically requires around 15 min of computing time for datasets containing around 10,000 variables and 1–2 h for those containing 1 around million variables, not including the time required to generate the primary effect sizes and standard errors on which the method depends. We used 20 threads for priorityFDR analysis of our approximately 6 million GWAS SNPs, on a single core, which required 10 h and access to 12.3 GB of RAM. Estimates are produced for both ‘local’ priorityFDRs, namely the priorityFDR for a single variable conditional on its observed data, and tail‐area priorityFDRs: the mean priorityFDR for variables exceeding a given local priorityFDR threshold (here we use “priorityFDR” to refer to tail‐area estimates unless stated otherwise).

As users may also be interested in ranking each variable relative to the full distribution of effect sizes, not just the non‐null effect sizes, we also used the method to estimate this quantity, which we term the inclusive priorityFDR (priorityFDR^(inc)^). The null variables are easily included by multiplying the effect priority in Equation 1 by 1−π, representing the probability of drawing a non‐null variable at random, where π is the proportion of null variables (estimated during priorityFDR estimation), and the FDR by $1-\pi +\frac{1}{2}\pi$. This is because, if a variable is null, then all of the non‐null variants (1−π) and half the null variants ($\frac{1}{2}\pi$) exceed it when it is assumed that there are no ties in effect sizes:

(5) $${\text{priorityFDR}}_{i}^{\left(\text{inc}\right)}=\text{Pr}\left(\text{Effect size of random variable}>\text{Effect size of variable}i\right)={\text{FDR}}_{i}\times (1-\pi +\frac{1}{2}\pi )+\left[{\text{effect priority}}_{i}\times (1-\pi )+0\times \pi \right]\times (1-{\text{FDR}}_{i}),$$

assuming that

(6) $$\text{Pr}(\text{Effect size of random variable}>\text{Effect size of variable}i\text{|variable}i\text{is null})=(1-\pi +\frac{1}{2}\pi ).$$

As ${\text{FDR}}_{i}=\text{Pr}(\text{variable}i\;\text{is null})$, the first and second terms of Equation 5 sum over the probability that variable *i* is either null or non‐null, similar to how the regular priorityFDR in Equation 1 can be interpreted as the proportion of *non‐null* variable that exceed variable *i* in effect size. It can clearly be seen that, when power is very low and thus all variables are null, the priorityFDR (Equation 1) equals 1 whereas the priorityFDR^(inc)^ equals 0.5.

Users may find either approach useful, but a potential advantage of priorityFDR^(inc)^ is that if ${\text{effect priority}}_{i}$ decreases with increasing sample size owing to smaller effect variables featuring in the non‐null distribution, this is balanced by the factor 1−π increasing, providing stability across varying statistical power. On the other hand, it is possible for ${\text{effect priority}}_{i}$ to remain constant (or, in theory, increase) with increasing sample size due to large effect, low precision variables also becoming distinguishable from null variables, in which case the regular priorityFDR (Equation 1) may be more appropriate as ${\text{effect priority}}_{i}\times (1-\pi )$ will increase with the sample size. Furthermore, as already discussed, the priorityFDR is designed to be complementary to significance measures like P values and FDRs, for cases in which users wish to further prioritise among a set of variables that they believe mostly contains true positives. PriorityFDR^(inc)^ is less intuitively useful in this scenario, in which the user already assumes that the variable set contains effects (mostly) greater than zero. We proceeded on these bases using the regular priorityFDR, defined using Equation 1, except where stated otherwise.

### 2.2 Simulation Studies

**Figure 1:**
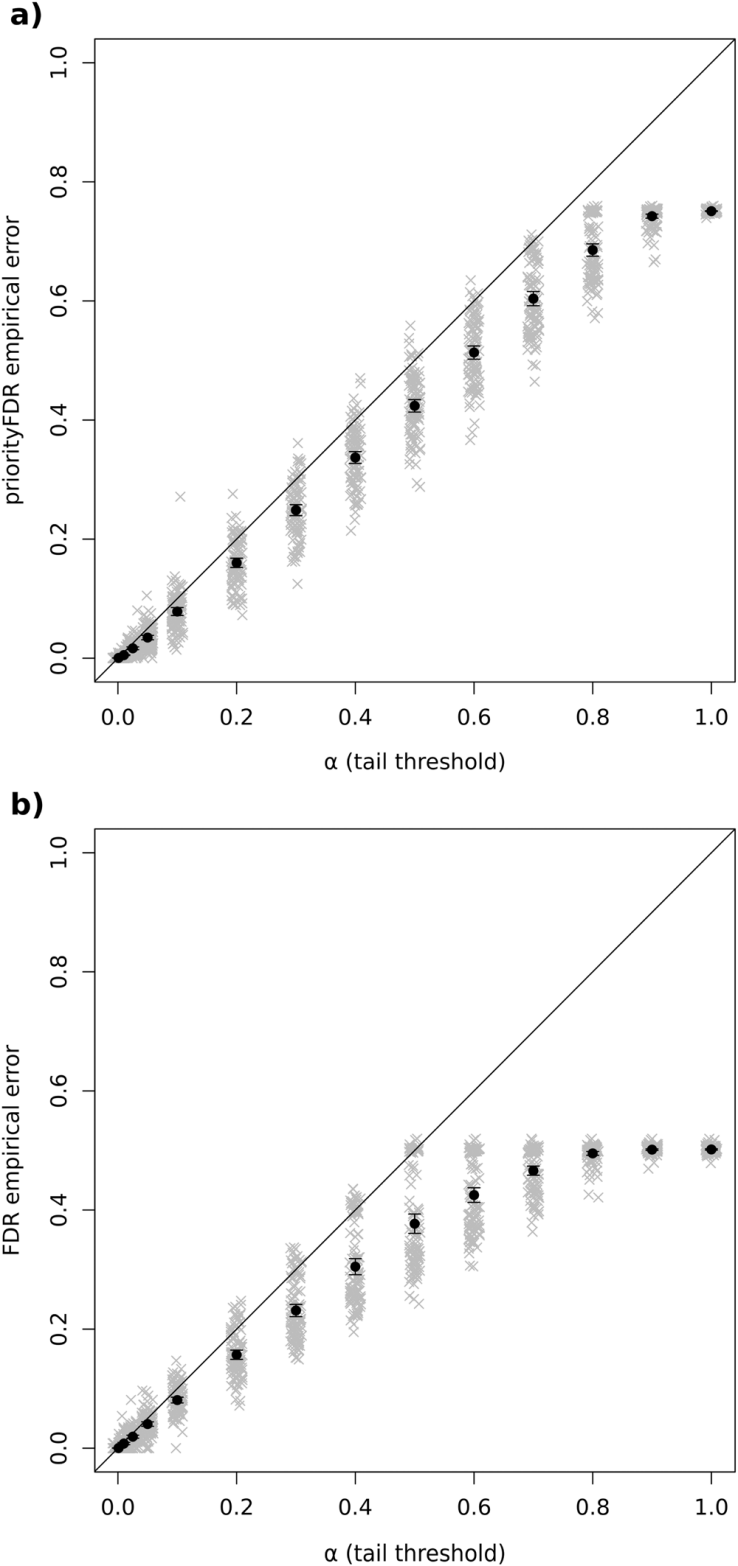
Average error rates of (a) priorityFDR and (b) FDR estimates for 100 GWAS simulations at 14 α thresholds (10^−3^, 10^−2^, 2.5 × 10^−2^, 5 × 10^−2^, and 0.1 to 1 in increments of 0.1) in grey, with means and 95% confidence intervals of the grey points shown in black. Means and upper confidence limits fell below the 1:1 line, indicating good error control. FDR empirical error (i.e. the proportion of null variables passing the threshold) approached 0.5 as α increased to 1, as half of the variables in our simulations had null effects of zero. Jitter was added to x‐axis values to aid visualisation. For computational reasons, each GWAS simulation contained 10,000 independent variants.

To test the method, we randomly simulated 100 effect‐size distributions using randomly chosen mixtures of normal distributions, representing 100 different biological phenomena, e.g. phenotypes with different genetic architectures, before simulating true and estimated effect sizes for 10,000 variables, representing a data set, from each of the 100 distributions (see materials and methods, example in Supporting Inforamtion S2: Figure S1a). In each simulated data set, the extent to which effect sizes varied with standard error, that is precision of the estimate, was randomly decided, so that low precision variables (e.g. rare genetic variants) might have either a similar or much more variable true effect size distribution compared to high precision variables (e.g. common genetic variants). Half of the true effect sizes were zero, i.e. true null effects, in all 100 distributions. We ran priorityFDR estimation separately on each simulated data set, returning tail‐area estimates of priorityFDR and FDR for each variable (FDRs are estimated as part of the priorityFDR model). We thresholded variables based on these two quantities, at several levels of error control (α), and then computed the empirical error rates for variables passing each threshold, using the known true effects. Variables with priorityFDRs passing a given α threshold had, on average, empirical error rates lower than α (Figure 1a), indicating good error control, and we found the equivalent was true for the standard FDR (Figure 1b). The FDR approached 0.5 as α increased to 1 as half of the simulated variables were null. As the effect priority always approaches 0.5 as α increases to 1, this had the effect of our priorityFDR approaching 0.75.

**Figure 2:**
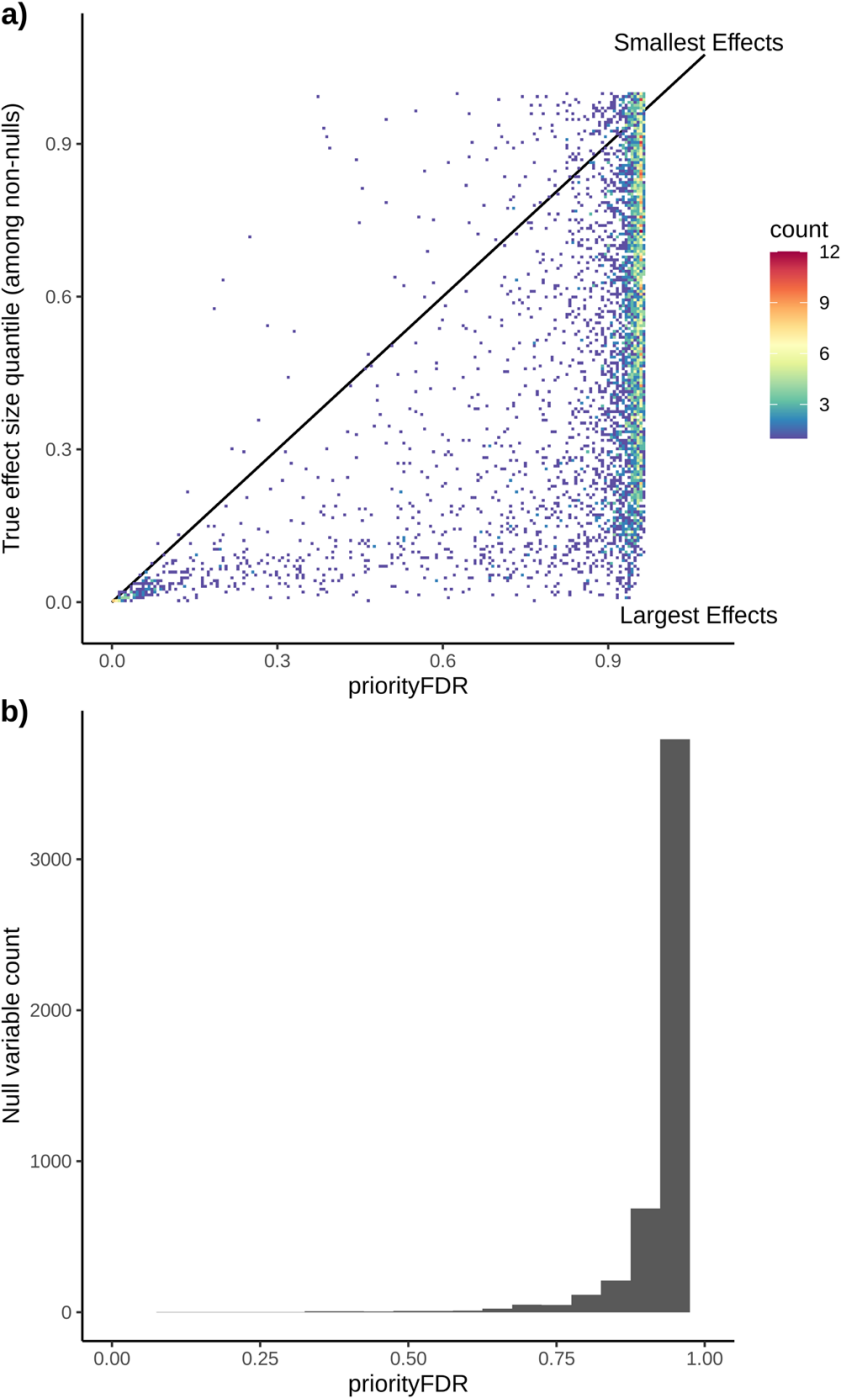
Estimated priorityFDRs versus true effect size rankings for a single simulated data set. Non‐null variables (*n* ≈ 5 K) are shown in (a) plotted against their true effect size ranks against all other non‐null variables, and the distribution of priorityFDRs among null variables (*n* ≈ 5 K), which have true effects of zero, are shown in (b). Plotted priorityFDRs are local rather than tail‐area estimates.

Within a single simulated data set (Z‐score distribution, Supporting Information S2: Figure S1a), estimated local priorityFDRs for non‐null variables displayed an approximately linear relationship with the true effect size rank quantiles, but were somewhat conservative, laying mostly beneath the 1:1 line (Figure 2a). Variables with low‐ranking, but non‐null, true effect sizes had local priorityFDRs mostly close to 1, indicating that they cannot usually be distinguished from null variables at the simulated level of power. A re‐simulation of this data set at higher power (Supporting Information S2: Figure S1b) followed by priorityFDR re‐estimation found a linear relationship closer to the 1:1 line (Supporting Information S2: Figure S2a).

At the original power, distribution of local priorityFDRs among null variables was highly concentrated above 0.75, verifying that the method is not prone to inferring null variables as having high ranking effect sizes (Figure 2b). In the higher‐powered data set this effect was less pronounced, probably due to there being 1/5th the number of simulated null variables and thus a lower prior null probability, but priorityFDRs were still concentrated above 0.5 (Supporting Information S2: Figure S2b). We found similar approximately linear relationships between local priorityFDR^(inc)^ and the effect size rank quantile, when ranking among both null and non‐null variables together, at both levels of power (Supporting Information S2: Figures S3 and S4), illustrating that priorityFDR^(inc)^ is the appropriate method for estimating an overall rank of an effect size among all variables. Again, the linear relationship was close to the 1:1 line in the highly powered data set (Supporting Information S2: Figure S4).

The relationship between true quantiles and estimated priorityFDRs was similar across different levels of significance, with variables in FDR ≤ 1%, 0.5% and 0.1% categories, using the same simulated datasets, showing a conservative linear relationship below the 1:1 line, and a linear relationship closer to 1:1 when power was higher, suggesting that performance is stable when prioritising among variables that appear, to the investigator, to be true positives (Supporting Information S2: Figure S5). Consistent with its intended usage, priorityFDR model fitting was performed using all simulated variables, not just those passing the FDR thresholds. Error rates recomputed, in the lower power data set, across subsets of variables satisfying FDR ≤ 1%, 0.5% and 0.1% thresholds were still correctly controlled, albeit very conservatively at higher priorityFDR thresholds (Supporting Information S2: Figure S6). This is expected as variables with low FDRs will tend to have low priorityFDRs.

To compare priorityFDR^(inc)^ against EBrank, we ran both methods on the simulated datasets and computed two types of error rate: (a) the proportion of variables in the top *x*% effect sizes that the method correctly assigned a rank quantile of *x*% or lower (analogous to power, where larger proportions are more desirable), and (b) the mean of the true effect size rank quantile taken over largest set of variables that the method claims has a true mean rank less than *x*% (analogous to type I error, where values close to or less than *x*% are desirable). Both error rates were computed at 5%, 1% and 0.5% thresholds of effect size quantile. Table 1a displays these results plus those for 100 further simulations using 1,000, rather than 10,000, simulated variables, and Table 1b shows the equivalent results for simulations using mixtures of Laplacian (double exponential), rather than normal, effect size distributions, which have broader tails producing more outliers. The performance of priorityFDR^(inc)^ was slightly stronger than EBrank, with an ability to detect the variables reaching high rank thresholds ranging from 14.5% better to 3.1% worse depending on the threshold and number of simulated variables (first three columns in Table 1), and was better in 11 out of the 12 comparisons while retaining correct average predictions for variables deemed to be highly ranking (second three columns in Table 1). PriorityFDR^(inc)^ had slightly less ability to predict ranks well for the top 0.5% of variables under the Laplacian simulations with only 1000 variables (0.370 vs. 0.382), although these values lie within 1 standard error of each other (SEs = 0.031 and 0.027 respectively, obtained from the SDs in Table 1). For the Laplacian simulations using 10,000 variables, priorityFDR^(inc)^ gave a slightly too high average rank quantile estimate of 0.006 for variables in the top 0.5%, but again this fell within one standard error (SE = 0.001) of the EBrank value (0.005), so could be attributable to sampling error which may disappear after further simulations. PriorityFDR had mostly higher variance in performance than EBrank between simulations (standard deviations in Table 1), which may stem from its greater modelling flexibility making it more prone to variance rather than bias.

**Table 1:** Performance of priorityFDR^(inc)^ versus EBrank in simulated datasets with (a) mixed‐normal distributions of effects and (b) mixed double exponential (Laplacian) distributions of effects, using both 1000 and 10,000 simulated variables, each across 100 simulations.

a) Mixed normal effect simulations							
		Proportion of top x% variables ranked in top x%			Mean true rank quantile of variables with mean predicted rank quantile of x%		
		5.0%	1.0%	0.5%	5.0%	1.0%	0.5%
**1000 variables**	priorityFDR^(inc)^	0.230 (0.167)	0.288 (0.231)	0.282 (0.286)	0.045 (0.035)	0.009 (0.017)	0.003 (0.003)
	EBrank	0.212 (0.162)	0.259 (0.211)	0.278 (0.273)	0.034 (0.024)	0.005 (0.004)	0.002 (0.002)
**10,000 variables**	priorityFDR^(inc)^	0.197 (0.136)	0.245 (0.165)	0.248 (0.186)	0.043 (0.015)	0.010 (0.006)	0.005 (0.003)
	EBrank	0.194 (0.122)	0.214 (0.153)	0.229 (0.176)	0.040 (0.010)	0.007 (0.004)	0.003 (0.002)

b) Mixed Laplacian effect simulations							
		Proportion of top x% variables ranked in top x%			Mean true rank quantile of variables with mean predicted rank quantile of x%		
		5.0%	1.0%	0.5%	5.0%	1.0%	0.5%
**1000 variables**	priorityFDR^(inc)^	0.213 (0.144)	0.354 (0.243)	0.370 (0.307)	0.040 (0.029)	0.009 (0.018)	0.003 (0.003)
	EBrank	0.212 (0.145)	0.333 (0.228)	0.382 (0.274)	0.037 (0.025)	0.007 (0.013)	0.003 (0.002)
**10,000 variables**	priorityFDR^(inc)^	0.202 (0.136)	0.335 (0.188)	0.371 (0.214)	0.043 (0.012)	0.010 (0.008)	0.006 (0.012)
	EBrank	0.195 (0.127)	0.296 (0.172)	0.348 (0.191)	0.041 (0.010)	0.008 (0.012)	0.005 (0.016)

*Note:* The first three columns of results, similar to statistical power, show the proportion of times that the method correctly identifies that a variable within the top x% of effect sizes does lie in that top x%, averaged over the 100 simulations. Larger values indicate greater success. The second 3 columns, similar to the type 1 error rate, show the average true rank of the set of variables identified as having a mean rank of x%, averaged over the 100 simulations. Values similar to or below x% indicate success of the method. Standard deviations of each value, taken across the 100 simulations, are shown in parentheses (divide each by 10 to obtain standard errors, corresponding to 100 simulations).

PriorityFDR does not account for dependence between variables, but we applied it to GWAS data containing linkage disequilibrium (LD) structure as standard methods of FDR control remain generally accurate under positive dependency between test statistics (Benjamini and Yekutieli 2001; Brzyski et al. 2017). To assess performance under LD, we generated simulated datasets similar to those above, but incorporating correlations between variables' effect sizes (see materials and methods). PriorityFDR and FDR performance was similar to when no correlations were simulated, and error rates were correctly controlled on average (Supporting Information S2: Figure S7 vs. Figure 1). If one wanted to perform priorityFDR for the causal effects of genetic variants taking LD into account, one approach would be to correct the marginal effect estimate sizes by regressing the corresponding chi‐square statistics against the LD scores constructed from all other variants within a physical window, e.g. 1 Mb in size, before performing priorityFDR analysis on the residuals after restoring the sign and scale of the original effect estimate.

### 2.3 Type 1 Diabetes Genome‐Wide Association Study Meta‐Analysis

Imputation, quality control (QC) and GWAS analysis was performed on Illumina (Infinium 550K, 3983 cases, 3994 controls) and Affymetrix (GeneChip 500K, 1926 cases, 3342 controls) SNP array‐genotyped UK samples, and on SNP array‐genotyped samples from Sardinia (Affymetrix 6.0 and Illumina Omni Express, 1558 cases and 2882 controls). Affected‐offspring trios (*n* = 3173) from Type 1 diabetes Genetics Consortium (T1DGC) were genotyped on the Illumina Human Core Exome beadchip and analysed with the transmission disequilibrium test (TDT), after imputation and QC. Results from the four cohorts were meta‐analysed under the additive model, together data from FinnGen (4933 cases and 148,190 controls). This constitutes the largest number of T1D cases analysed by GWAS to date, with 15,573 cases and 158,408 controls (Supporting Information S4: Table S1). Note that larger studies have been performed using the Immunochip SNP array, which concentrates genotyping around known and candidate immune loci (Chiou et al. 2021; Robertson et al. 2021).

We saw an inflation of meta‐analysed Chi‐square statistics (ratio of the observed median statistic over the null median of 0.456, $\lambda_{\text{GC}}$ = 1.12, quantile‐quantile plot in Supporting Information S2: Figure S9). Analysis of the T1DGC family cohort, using the TDT, is not subject to population stratification. Inflation was comparable within this cohort alone ($\lambda_{\text{GC}}$ = 1.09), consistent with polygenicity of T1D, rather than population stratification, being the major cause of test statistic inflation observed in the meta‐analysis. Applying LD‐Score regression gave an inflation factor estimate of 0.99, where values above 1 imply inflation in significance that is not due to polygenic causal associations, implying that our GWAS results are unbiased by the effects of population stratification (Bulik‐Sullivan et al. 2015).

LD‐based filtering found 201 independent T1D‐associated signals with FDR ≤ 1% (Supporting Information S2: Figure S8, Supporting Information S4: Table S2), where we used ‘independent signals' to refer to a disease‐associated variant or set of genetic variants in LD (r^2^ > 0.01), related to, but distinct, from a physical region. LD computations were performed up to physical distances of 1 Mb. We refer to the most significant variant in the signal as the lead variant, representing the overall signal in terms of effect size and significance. The largest *p* value among our 201 lead variants was 2.67 × 10^−5^.

Of these, 88 can be considered ‘new’ (Supporting Information S4: Table S3), being independent (r^2^ < 0.01) from any lead variants in neither the previously most highly powered GWAS/ImmunoChip studies (Barrett et al. 2009; Onengut‐Gumuscu et al. 2015), a significantly larger recent ImmunoChip study (Robertson et al. 2021), or a large recent GWAS (Chiou et al. 2021). Four of these new signals (near *RLIMP2*, *SLC25A37*, *MAGI3* and *LHFPL5*, Table 2) were new at genome‐wide significance, *p* ≤ 5 × 10^−8^, with the remaining 84 at FDR ≤ 1% but not at the genome‐wide threshold (*p* values ranging 7.12 × 10^−8^ to 2.67 × 10^−5^). Note that signals are named after the gene closest to the lead variant, although this may not be the causal gene. The new genome‐wide significant signals displayed a range of estimated effect sizes, with ORs for the risk variant (OR_risk_) higher for *RLIMP2* and *SLC25A37*, at 1.18 (95% CI 1.12–1.24) and 1.17 (1.11–1.22), than for *MAGI3* and *LHFPL5* at 1.12 (1.08–1.16) and 1.09 (1.06–1.13). Lead variants for all four signals had relatively common minor alleles (MAFs 7.32–29.86%). Among the 84 new signals not reaching genome‐wide significance, we saw a wide range of estimated effect sizes (OR_risk_ 1.07–1.48), with MAFs ranging from 0.77% to 47.75%, with larger effects corresponding to lower MAFs. Among previously established signals reaching genome‐wide significance (*n* = 54), we found a similarly large range of effect sizes (OR_risk_ 1.08–1.79), with MAFs ranging from 3.93% to 49.85%.

**Table 2:** Novel T1D signals reaching genome‐wide significance, plus two signals with FDR ≤ 1% and with priorityFDRs comparable to the four genome‐wide significant signals.

Nearest gene	Band	ID	MAJ	MIN (effect)	MAF^a^	Imputation info^b^	*p*	OR (95% CI)	FDR	priorityFDR	Annotation	Significance threshold
RLIMP2	1p13.2	rs12130424	T	C	7.32%	99.75%	2.12x10^‐10^	1.18 (1.12‐1.24)	7.95x10^‐07^	2.87x10^‐04^	intergenic	Genome‐wide
SLC25A37	8p21.2	rs36104352	A	C	12.34%	99.50%	6.17x10^‐10^	0.86 (0.82‐0.9)	1.12x10^‐06^	3.94x10^‐04^	intergenic	Genome‐wide
MAGI3	1p13.2	rs6696593	C	T	22.54%	97.73%	2.58x10^‐09^	0.89 (0.86‐0.93)	4.40x10^‐06^	2.07x10^‐03^	regulatory region	Genome‐wide
LHFPL5	6p21.31	rs2817023	C	G	29.86%	96.39%	2.67x10^‐08^	1.09 (1.06‐1.13)	7.12x10^‐05^	4.90x10^‐03^	upstream gene	Genome‐wide
ID4	6p22.3	rs75356149	G	T	2.33%	98.69%	1.04x10^‐06^	1.29 (1.16‐1.42)	1.08x10^‐03^	3.31x10^‐03^	intergenic	FDR 1%
ZBTB20	3q13.31	rs56240698	G	C	11.35%	75.19%	1.66x10^‐06^	0.85 (0.79‐0.91)	9.83x10^‐04^	3.96x10^‐03^	intergenic	FDR 1%

*Note:* See Supporting Information S4: Tables S2 and S3 for details of all associated signals, ordered by risk effect size. All ORs are for minor alleles, and IDs are for each signal's lead variant.

a UK Illumina controls.

b UK Illumina all samples.

**Figure 3:**
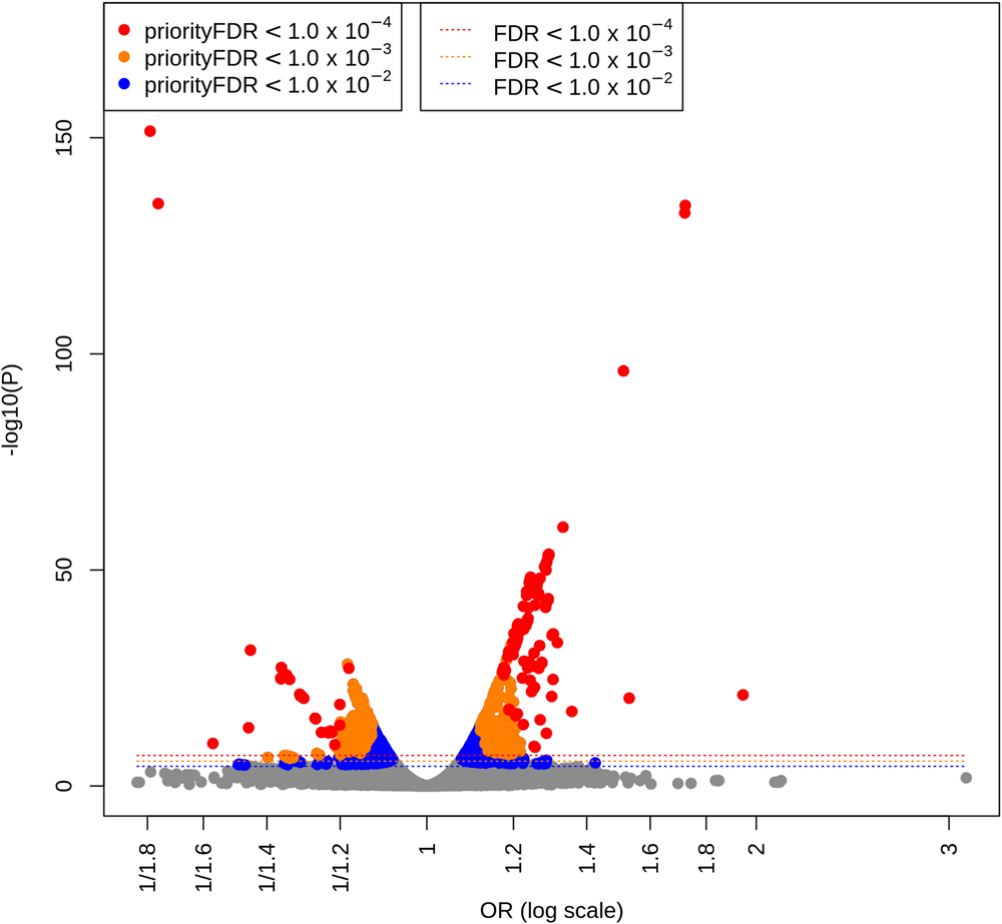
Volcano plot of genome‐wide minor allele T1D effect size (log odds ratio) versus significance (‐log10 *P* values), coloured by priorityFDR thresholds. All analysed variants are shown. Even stringent FDR thresholds, denoted by dashed lines, select variants with small ORs, whereas the equivalent priorityFDR thresholds select variants with increasingly larger effects.

The question of how to prioritise among these signals, which have widely ranging effect sizes and thus may be of highly variable biological importance, whether reaching genome‐wide or just FDR ≤ 1% levels of significance, is the purpose for which the priorityFDR was developed. The relationship between effect estimate size and statistical significance is illustrated genome‐wide by a volcano plot in Figure 3, showing that high levels of significance may be attained for relatively small effect estimates, but that generally the most highly significant variants have among the largest estimated effects. Variants are coloured according to whether they satisfy various priorityFDR thresholds, showing how the method selects on both effect size and significance. PriorityFDRs for the four new signals in Table 2 were all below 1%, indicating that they have true effects estimated to be larger than than 99% of non‐null associations, with the largest priorityFDR being 4.90 × 10^−3^ (*LHFPL5*). Two new signals that did not reach genome‐wide significance still had priorityFDRs below 4.90 × 10^−3^: *ID4*, with OR = 1.29 (1.16–1.42), and *ZBTB20*, with OR_risk_ = 1.18 (1.10–1.27), suggesting that these may be similarly worthy of follow up despite having lower levels of traditional significance. We found four other new signals satisfying these criteria (*NRSN1, RPL35AP21, MYC and NMNAT2*, Table S3), but highlight *ID4* and *ZBTB20* due to their low priorityFDRs and consistency of effects across cohorts (Figure S12). All 54 of the previously detected genome‐wide signals had priorityFDR ≤ 1%, but three (near *IKZF3*, *CCDC88B* and *C11orf30*) had priorityFDRs that were higher (less prioritised) than the four newly detected signals, with OR_risk_ ranging 1.08–1.09.

The correlation between FDRs and priorityFDRs across our 201 T1D GWAS lead variants was 0.89 (*r* ^2^ = 0.80), indicating that 80% of the priorityFDR variation was explained by the FDRs. The equivalent correlation between priorityFDRs and P‐values was 0.86 (*r* ^2^ = 0.74), explaining 74% of the variation. If one were to take the effect size as a measure of ‘biological importance’, this suggests that, at the level of power attained by our T1D GWAS, statistical significance as measured by *p*‐values or FDRs is a good predictor of biological importance, but can be substantially improved upon by using the priorityFDR.

We found that seven signals contained multiple conditional signals after stepwise model selection: *INS* (11p15.5), *PTPN22* (1p13.2), *IL2RA* (10p15.1), *PTPN2* (18p11.21), *RLIMP2* (1p13.2), *PRKCQ* (10p14) and *AKAP11* (13q14.11) (Supporting Information S4: Table S4), of which *RLIMP2* was the only new association. As meta‐analysis cohorts were genotyped on different platforms, fine‐mapping was performed using only the largest UK cohort (Illumina array genotypes) to avoid artefacts owing to varying imputation quality across platforms (Supporting Information S4: Table S5). We were, therefore, limited in our ability to fine‐map the new signals in Table 2, such that we were not able to attribute the *SLC25A37*, *LHFPL5*, or *ID4* signals to a specific credible set (Supporting Information S2: Figure S10). There was some evidence for two separate credible sets near to the *ZBTB20* signal, but neither could be narrowed down to a small number of variants (Supporting Information S2: Figure S10d). *RLIMP2* and *MAGI3* are located within 500 kb of each other in a region approximately 500 kb from the very strong T1D risk signal at *PTPN22* (rs2476601, OR = 1.78, *p* = 4.54 × 10^−135^). Although both signals had lead variants that were independent of rs2476601 (*r* ^2^ < 1%), as this is the threshold we used for defining separate signals (materials and methods), the presence of small amounts of long‐range LD with the strong *PTPN22* signal meant fine‐mapping was unlikely to be reliable in this region. While it is, therefore, difficult to ascribe causality to any of the lead variants, it is notable that the lead *MAGI3* signal variant (rs6696593) lies in an enhancer element approximately 70 kb upstream of the gene, and the lead *LHFPL5* signal variant lies less than 1 kb from its nearest promoter region. Three of the newly‐identified regions in Table 2 were associated at *P *≤ 5 × 10^‐4^ with other autoimmune diseases in the Open Targets Genetics portal: *RLIMP2* (RA and hypothyroidism), *SLC25A37* (hypothyroidism) and *ID4* (ulcerative colitis), further supporting their association with T1D (Supporting Information S4: Table S6).

### 2.4 PriorityFDR Selection of Type 1 Diabetes Signals

**Figure 4:**
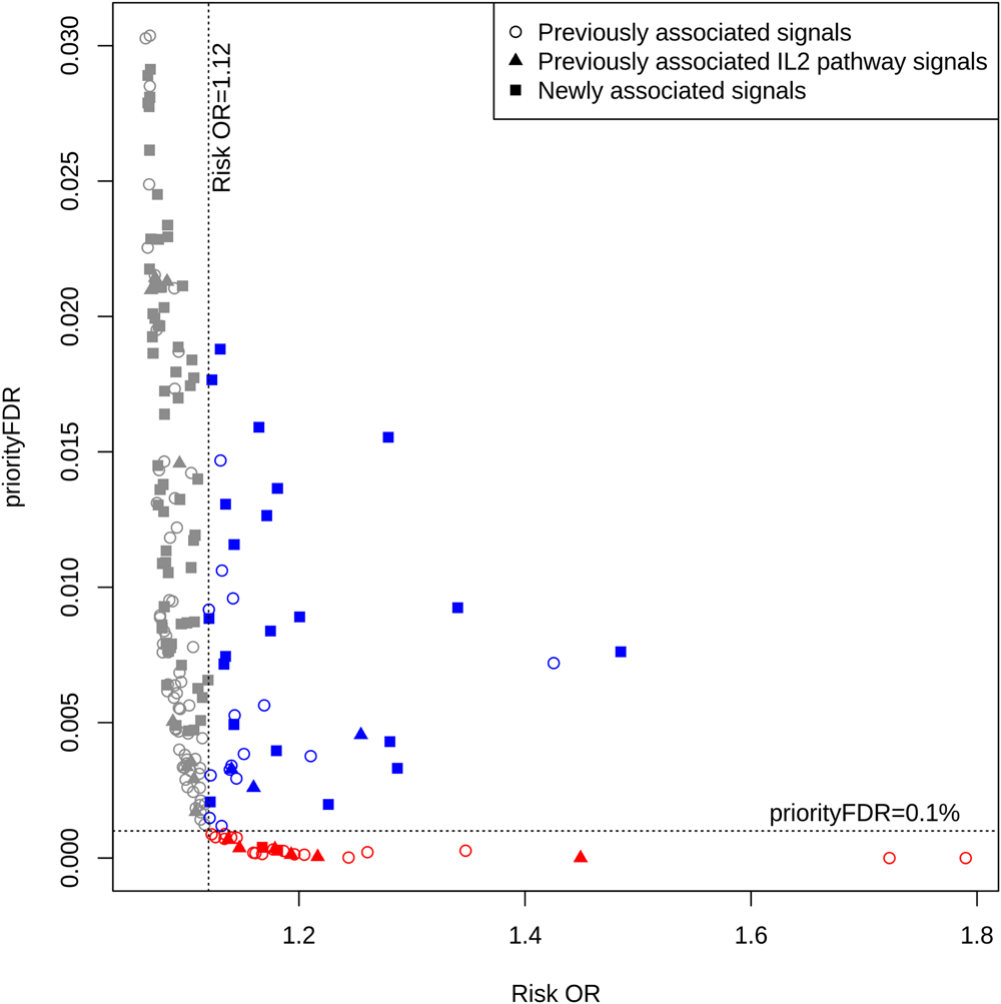
Two potential threshold strategies for prioritising variables using priorityFDRs, using the 201 independent T1D signals with FDR ≤ 1%. Circles and filled squares indicate previously and newly‐associated signals respectively. Red points (*n* = 26) pass a priorityFDR threshold of approximately 0.1%, an inflection point above which priorityFDRs start to rise steeply. The blue points (*n* = 39) have effect estimates greater than the smallest effect size in the red group (OR_risk_ ≈ 1.12), but are more statistically uncertain, so this might be used as more permissive threshold.

The fact that several of the novel signals had priorityFDRs within the range of the novel genome‐wide significant signals, despite not having genome‐wide significance themselves, suggests defining an objective priorityFDR threshold using the distribution of priorityFDRs observed among the signals. One objective way of choosing an important subset would be to find an approximate inflection point, by eye, above which the priorityFDRs rise sharply, and to exclude signals (or variables) above this threshold. We observe such an inflection point among the T1D signals at priorityFDR ≈ 0.1% (Figure 4), and refer to signals passing the threshold, highly likely to be both true positives and have large effect sizes, as the red group. This represents just one possible strategy for selecting among priorityFDRs, and users may, for example, prefer to equally prioritise all variables below a particular threshold, for example 1%, or may be limited by resources to pursuing a maximum number for example the smallest 10. A less stringent additional threshold could also be set at the minimum effect size that passes the first threshold (blue group of points in Figure 4), provided that this does not accept too many additional variables. This is because there may be a reasonable chance of some of the blue group's true effects being larger than some members of the red group, despite blue group effect estimates being more statistically variable, owing to lower MAF, for example, as the priorityFDR is an estimate rather than a known quantity. Blue group variables are higher‐risk for follow up, as priorityFDR analysis suggests that their expected effect size is lower than the red group members. However, if one were to disregard the prior information modelled via priorityFDR analysis, there would be some evidence that their their effect sizes are comparable to the red group, as indicated by the observed (maximum‐likelihood) estimates on the x‐axis of Figure 4. Although this approach selects directly on the effect size estimate, priorityFDRs must first be computed to find an appropriate effect estimate threshold. Across all SNPs in the T1D GWAS data set, we found no variants with OR_risk_ < 1.12 satisfying the 0.1% threshold, even with very small FDRs below 1 × 10^−4^ (Figure 3), demonstrating how priorityFDR selects only those variables with both high significance and relatively large effect sizes. This is because, as significance becomes increasingly strong and a variable's FDR gets closer to zero, the priorityFDR approaches the effect priority (see Equation 1), which thus forms a lower bound on the priorityFDR with respect to changes in FDR.

Variables meeting neither threshold (the grey group) would be de‐prioritised firstly due to having priorityFDRs that are mostly much higher than the red group, conveyed by the y‐axis threshold in Figure 4. Secondly, unlike the blue group, they have observed maximum likelihood effect sizes which suggest that their corresponding true effect sizes mostly don't overlap with those of the red group, conveyed by the x‐axis threshold, even if one were to disregard the Bayesian prior information incorporated by priorityFDR analysis.

Genes with roles in the IL‐2 pathway have led to many of most important biological insights into T1D biology, leading to clinical trials of low‐dose IL‐2, which upregulates the activity of regulatory T cells, for T1D therapy in children (Marcovecchio et al. 2020; Todd et al. 2016). To assess whether the priorityFDR selection approach in Figure 4 focusses on key biological mechanisms, we compiled a list of 23 known IL‐2 pathway genes (Supporting Information S4: Table S7), and found 29 of 201 lead variants were within 250 kb of one of these genes (Supporting Information S4: Table S8). We found overrepresentation of these in the red group (*n* = 26, *p* = 0.003, OR = 4.55), but not the blue group (*n* = 39, *p* = 0.396, OR = 1.58), using Fisher's exact test with the variants in neither group (the grey group) as controls (*n* = 136), suggesting that the priorityFDR selection approach may identify disproportionate numbers of biologically relevant effects in the red group, and possibly a smaller proportion in the blue group. An alternative selection of the inflection point at priorityFDR = 0.5% and OR_risk_ = 1.09 (Supporting Information S4: Figure S11) gave an even stronger enrichment of red group variants close to IL‐2 pathway genes (*p* = 7.04 × 10^−4^, OR = 6.13).

As a further test, we used the same method to analyse whether putatively causal T1D genes, inferred via MR of eQTL data, were disproportionately close to lead variants in the red or blue groups versus the grey group. We assembled a list of 219 putatively causal T1D genes from 13,499 with at least one genome‐wide significant genetic instrument, by performing MR analysis relating gene expression level causal effects on T1D risk and selecting those with significant MR effects at FDR ≤ 1%. We identified which T1D lead variants were within 250 kb of one of the putatively causal genes and tested for association with red and blue group membership, initially without variants close to IL‐2 pathway genes to maintain independence from the previous analysis (Table 3a). Red group lead variants were significantly overrepresented near MR significant genes, including when removing the 29 variants near IL‐2 pathway genes (*p* = 0.005, O = 5.98), thereby providing additional evidence that they have important biological effects. Similar to the IL‐2 pathway analysis, blue group lead variants had reduced overrepresentation which was not significant (*p* = 0.110, OR = 2.47), although the combined association of both groups was significant (*p* = 0.010, OR = 3.51). Results were similar after including lead variants near IL‐2 pathway genes (Table 3b). See Supporting Information S4: Table S8 for priorityFDR group memberships of T1D signals, plus whether they are proximal to IL‐2 pathway or MR genes.

**Table 3:** Association of T1D signals’ priorityFDR/effect size categories (from Figure 4) with putatively causal MR effect sizes of nearby genes’ expression levels on T1D risk, with signals near IL‐2 pathway genes (a) excluded and (b) included.

(a) Excluding T1D GWAS signals near IL‐2 pathway genes (*n* = 183)						
	Near MR associated gene	Not near MR associated gene	*p*	*OR*	*p* (combined)	*OR* (combined)
Red group	6	11	0.005	5.983	0.01	3.506
Blue group	6	27	0.11	2.471		
Grey group	10	112	—	—	—	—

(b) All T1D signals (*n* = 201)						
	Near MR associated gene	Not near MR associated gene	*p*	*OR*	*p* (combined)	*OR* (combined)
Red group	8	18	0.005	4.532	0.004	3.351
Blue group	8	31	0.082	2.649		
Grey group	12	124	—	—	—	—

*Note:* ORs and *p* values (Fisher's exact test) are for red and blue groups (and both combined) relative to the grey group.

Some previously detected signals in the red group, for example those near *PRF1*, *SH2B3*, *FKBP5* (near *DEF6*) and *SLC1A5* (near *PRKD2*) (Supporting Information S4: Table S8), may be less widely recognised than others, like those near IL‐2 pathway genes. All but four out of 26 red group signals (*PRF1*, *CTRB*2, *MEG3* and *RNLS*) were associated with other autoimmune diseases found in the Open Targets Genetics portal (Supporting Information S4: Table S6).

The blue group consisted of signals with similar effect size estimates to the red group, but with higher standard errors, indicating greater uncertainty in the true effect size, leading to higher priorityFDR estimates (see Supporting Information S2: Figure S12 for meta‐analysis estimates and confidence intervals in each group). In some cases (22/39 signals), this was because we had removed the lead variant from the T1DGC data set if it showed Mendelian inconsistencies between parents and offspring, a consequence of imputing genotypes in family samples. Three further signals were present in T1DGC but absent in FinnGen, and another three were present in both datasets but had MAF < 5%, thus also having relatively large standard errors. In total 9/39 blue group signals had MAF < 5% and 2/39 were below 1%, whereas only 2/26 red group MAFs were below 5% and none were below 1%.

Around half (20/39, 51%) of the blue group lead variants were associated with other autoimmune traits (Supporting Information S4: Table S6), which was lower proportionally than in the grey group (77/136, 57% of lead variants), although not significantly so (Fisher's exact *p* value = 0.59). The blue group contained many more new T1D associations (*n* = 21) than the red group (*n* = 2, *RLIMP2* and *SLC25A37*), only one of which, *MAGI3*, was genome‐wide significant (*p* = 2.58 × 10^−9^). Fine‐mapping these signals is challenging, but nine of the 21 lead variants lie in noncoding transcripts or regulatory regions, suggesting they may be worth further investigation. Six of the 21 had priorityFDRs lower than at least one genome‐wide significant signal (largest genome‐wide significant priorityFDR observed = 0.64%), despite only one being genome‐wide significant itself. Only one red group signal, *FKBP5*, was not genome‐wide significant (*p* = 8.10 × 10^−8^).

## 3 Discussion

We developed the priorityFDR as an extension to the FDR, measuring the probability that a variable is either null or is exceeded in size by a randomly chosen non‐null effect, equivalent to estimating the effect size rank among non‐null variables. When applied to a set of variables assumed to be mostly true positives, due to satisfying an FDR or other type of significance threshold for example Bonferroni correction, the priorityFDR provides a means of choosing a smaller set of variants to follow up on the assumption that those with larger effects are more likely to yield to promising biological discoveries. This is likely to be helpful in large modern datasets, for example biobanks, where thresholding on significance alone may produce very large numbers of variables, and following up only the most highly significant may overlook important true effects. Our method is also capable of ranking variables' effect sizes relative to all variables in the data set, both null and non‐null (priorityFDR^(inc)^), which might be used as an alternative method of analysis, more appropriate when variables haven't already been selected for significance. PriorityFDR^(inc)^ performed well compared to EBrank in our simulated datasets, in most cases more accurately, but had slightly higher variability in performance between datasets. However, as priorityFDR^(inc)^ uses a novel approach to estimation like priorityFDR, basing all inferences on estimating likelihoods for observed data with positive and negative true effects, rather than estimating the prior distribution of true effects, it is possible that many further improvements can be made. It is likely that the average advantage that priorityFDR^(inc)^ displayed in simulations are due to it not having to estimate the prior distribution of effects, which can be challenging (Efron and Hastie 2016). EBrank is computationally faster than priorityFDR^(inc)^ in its current implementation, but this might be improved in several ways for example by implementing aspects of the method in a compiled language. Although further simulation studies will help to establish which method is more effective in different circumstances, our results demonstrate that priorityFDR^(inc)^ performs at least similarly as well as EBrank.

Our main statistical innovation for estimating the priorityFDR is the prior splitting method, which allows the distribution of pairwise between‐variable effect estimate differences to be modelled as a mixture of negative, null (zero) and positive differences, using the distribution of observed effect estimates (likelihood) directly. This is achieved by constraining the relationship of the negative and positive mixture component distributions with the null distribution, in such a way that they contain only negative and positive true effects, respectively. For putative positive and negative effects, one can then repeat a similar procedure to compute the posterior probability that they are exceeded in effect size by a randomly chosen variable, allowing one to estimate the priorityFDR (see Supporting Information S1: Appendix S1 for full details). Our simulations showed that the method controls the priorityFDR under a variety of simulated effect distributions.

It is acknowledged that many, even most, regions of the genome are likely to contain non‐zero effects on many complex phenotypes (Boyle, Li, and Pritchard 2017; Crouch and Bodmer 2020; O'Connor 2021). A large proportion of small‐effect ‘polygenic’ risk variants are thus likely to be discovered in a well‐powered GWAS, especially when using FDRs rather than more conservative Bonferroni‐corrected *p* values. Increasing availability of even larger genome‐wide SNP and sequencing datasets will detect increasing numbers of these associations, while improving the accuracy of genetic risk scores (Visscher et al. 2021) and explaining further heritability (Yengo et al. 2022). However, some associations detected for lower MAF variants may have larger effects, despite modest levels of significance, as sample sizes increase further and sequencing technologies improve, and it is important to be able to identify these variants that may be highly biologically relevant.

Conducting the largest T1D GWAS to date allowed us to find four novel signals reaching genome‐wide significance (near *RLIMP2, SLC25A37, MAGI3* and *LHFPL5*), but priorityFDR analysis allowed us to detect additional new signals, all satisfying FDR ≤ 1%, that may also be worthy of follow up. First, we found that several FDR ≤ 1% signals, including two near *ID4* and *ZBTB20,* had priorityFDRs lower than *LHFPL5*, suggesting that they might be equally worth pursuing. The *ID4* lead variant is associated with a large increase in ulcerative colitis risk (de Lange et al. 2017) (*p* = 1.59 × 10^−7^, OR = 1.32), providing further evidence that it may play an important role in T1D aetiology.

Secondly, we ranked FDR ≤ 1% signals by effect size and thresholded at the inflection point where their priorityFDR starts to rapidly rise, thereby creating a set of variables with qualitatively similar, low priorityFDRs, termed the ‘red’ group. Although the red group contained mostly previously known associations, the exceptions being *RLIMP2* and *SLC25A37*, another less stringent set was defined with higher priorityFDRs but effect size estimates that would qualify them for membership of the red group if their standard errors were lower (the “blue” group). This set contained 21 new signals, including *ID4* and *ZBTB20*. We found the red group to be significantly overrepresented for lead variants that were within 250 kb of (a) genes in the IL‐2 pathway, one of the most important pathways in T1D aetiology, and (b) putatively causal T1D risk genes as inferred using MR. The same trends were apparent when combining the red and blue groups together, although were not significant at 5% in the blue group alone. These results suggest that the red group, and possibly to a lesser degree the blue group, are enriched for biologically important effects. The presence of the *ID4* signal in the blue group is consistent with some members of this set playing important roles, with others have smaller true effect sizes. We found that nine of the 21 novel blue group lead variants were located in regulatory regions or noncoding transcripts, and seven were associated with other immune traits. For example, rs35859457 near *TRNP1* is located in a transcription factor binding site, and associates with basophil percentage (Astle et al. 2016).

One of our findings from applying priorityFDR to T1D GWAS data is that, when many genome‐wide associated signals are present, signals with fairly large stand out effects can be overlooked, perhaps due to the presence of others with higher significance or a more obvious biological connection to the phenotype. For example, *SH2B3* and *PRF1* are relatively understudied as causal genes for T1D, despite belonging to the red group and having priorityFDRs comparable to the largest T1D associations. However, mechanistic understanding can also be attained from signals that are less highly prioritised by priorityFDR. Recent work reports evidence that *CFTR* is a causal T1D gene (Chiou et al. 2021), even though the GWAS signal proximal to this gene, at 7q31.2, has a relatively small effect, with priorityFDR = 8.88 × 10^−3^ and OR = 1.08 (1.05–1.11), and was not included in either red or blue prioritisation groups.

Disease‐associated genomic regions with high ranking effect sizes, selected via objectively chosen priorityFDR thresholds, can be prioritised for further analyses and integrated with the emerging wealth of whole genome sequence, mRNA and protein QTL data (Koprulu et al. 2023; Rasooly et al. 2023; Sun et al. 2022), improving our understanding of physiology, disease diagnosis, initiation, progression and prevention, and the success rate in drug development pipelines.

## 4 Materials and Methods

### 4.1 PriorityFDR Simulation Experiments

For 100 effect architectures representing different biological phenomena under study, we simulated a mixture of 19 normal distributions with mean zero and SDs randomly drawn from an exponential distribution, plus a point‐null distribution (SD = 0) as the 20th mixture component. Exponential rate parameters were chosen separately for each architecture by drawing 100 random uniform variables between 0.1 and 1, allowing variability in signal/noise ratios between architectures. Mixing proportions were simulated from a Dirichlet distribution parameterised by the vector $e^{-s_{k}}/\sum_{k=1}^{19}e^{-s_{k}}$ divided by its median value, where *k* identifies a mixture distribution and $s_{k}$ is its SD, such that smaller mixing proportions were favoured for distributions with large SDs, consistent with realistic biological effect architectures (e.g. genetic risk architectures). The 19 resulting mixture proportions were standardised to sum to 0.5, with a 20th mixture proportion of 0.5 corresponding to the null mixture. We sampled 10,000 multinomial variables from the vector of 20 mixture proportions, providing the mixture memberships for each variable, and for each variable then drew a random sample from the corresponding normal distribution, representing its true effect size on the Z‐sore scale, θ. Assuming that each observed variable is binomial, like a SNP variant, frequencies were simulated from a beta distribution with shape parameters equal to 0.8, after which they were scaled to lie between 0.5% and 99.5% (reflecting how variants with MAF < 0.5% are typically not analysed in GWAS) and approximate standard errors (SEs) for the log OR estimates calculated as:

$${\hat{\sigma }}_{i}=\frac{1}{\sqrt{N}}\sqrt{\frac{1}{{\text{MAF}}_{i}\times c}+\frac{1}{(1-{\text{MAF}}_{i})\times c}+\frac{1}{{\text{MAF}}_{i}\times (1-c)}+\frac{1}{\left(1-{\text{MAF}}_{i})\times (1-c\right)}},$$

where *N* is the sample size, *c* the proportion of cases, which we set to 15,000 and 0.5 respectively, and *i* a variable (e.g. SNP) identifier. Rather than simply taking true effect sizes (log ORs) to be $\beta_{i}=\theta_{i}{\hat{\sigma }}_{i}$, following the definition of $\theta_{i}$ on the Z‐score scale, we allowed for the relationship between SE (${\hat{\sigma }}_{i}$) and effect size to differ between architectures by setting:

$$\beta_{i}=\theta_{i}(\gamma {\hat{\sigma }}_{i}+(1-\gamma )\bar{\sigma }),$$

where γ was sampled separately for each genetic architecture from a uniform distribution bounded by 0 and 1, and $\bar{\sigma }$ was the mean ${\hat{\sigma }}_{i}$ across all variables within the simulation. High γ therefore induces a strong positive relationship between effect size and SE, as larger SEs amplify $\theta_{i}$ further from zero. Estimated log ORs were then simulated as ${\hat{\beta }}_{i}=\beta_{i}+\varepsilon_{i}$ where $\varepsilon_{i}$ is a normal random variable with mean zero and SD ${\hat{\sigma }}_{i}$. Z‐scores, ${\hat{\beta }}_{i}/{\hat{\sigma }}_{i}$, provide an indication of how much signal is present, and are shown for an individual simulated data set in Supporting Information S2: Figure S1a. PriorityFDR estimation, returning tail‐area estimates of priorityFDRs, FDRs and effect priorities (see Appendix: Supporting Information S1) for each variable, was run using priorsplitteR with inputs ${\hat{\beta }}_{i}$ and ${\hat{\sigma }}_{i}$ (for all variables *i* in 1, 2…10,000), for each simulation separately.

After priorityFDR estimation, variables were thresholded according to priorityFDR, effect priority and FDR tail‐area probabilities, using α thresholds of 0.001, 0.01, 0.025, 0.05, 0.1, 0.2, 0.3… 0.9 and 1. At each value of α, we recorded the true error for each variable *i* with estimated priorityFDR ≤ α, to quantify how well this error was controlled by the estimation method, based on true FDR and effect priority errors:

$${\text{err}(i)}_{\text{FDR}}=I(\beta_{i}=0),$$

$${\text{err}(i)}_{\text{effect priority}}=\sum_{j}I(\beta_{j}\neq 0)I(\left|\beta_{j}\right|>\left|\beta_{i}\right|)/\sum_{j}I(\beta_{j}\neq 0),$$

$${\text{err}(i)}_{\text{priorityFDR}}={\text{err}(i)}_{\text{FDR}}+\left(1-\text{err}{\left(i\right)}_{\text{FDR}}\right){\times \text{err}(i)}_{\text{effect priority}},$$

where I(⋅) is an indicator function taking the value 1 if the condition in parentheses is met or 0 otherwise, and *j* indexes a variable in the dataset, with the summations being taken over all variables. As ${\text{err}(i)}_{\text{effect priority}}=1$ when $\beta_{i}=0$, ${\text{err}(i)}_{\text{priorityFDR}}$ is equal to ${\text{err}(i)}_{\text{effect priority}}$. The true FDR error is 1 if the variable is null and 0 if it is non‐null, whereas the true effect priority error is the proportion of non‐null variables with true effect sizes larger than variable *i*. The true priorityFDR error is an empirical version of Equation 1 using the known true effects from the simulation. Means of each of the three errors were taken over *i* for variables passing the different α thresholds, within each architecture (Figure 1). We also took mean errors among variables passing both a given α threshold for the priorityFDR plus a threshold on the FDR (see main text and Supporting Information S2: Figure S6).

To assess the effect of increased statistical power on the relationship between estimated priorityFDRs and true effect sizes within a single simulated data set, we multiplied the standard deviation of each mixture distribution, $s_{k},$ by 4, and changed the proportion of null variables from 0.5 to 0.1 (Supporting Information S2: Figure S1b).

Correlations between variables' effect sizes (manifest as LD in genetic datasets) were simulated by first assigning variables randomly into 100 blocks each containing 100 variables. Variables were assigned randomly to blocks with replacement, so were allowed to fall into more than one block. A randomly sampled normal variable with mean 0 and SD of 0.1 was drawn for each block separately, and the absolute value taken, representing a shared shift in observed effect size, on the Z‐score scale, across the block. We subtracted the theoretical expected value, approximately 0.0798, from each shared shift variable, so as not to introduce undue additional signal into the data. Because variables usually differ in how strongly they associate with the other variables in their block, we also simulated uniform variables between 0 and 1, for each variable in the block, with 0 representing no association with the rest of the block, and 1 the highest possible association. These were multiplied by the block's shared effect size shift, the resulting product added to the absolute Z‐score for each variable in the block, and this sum then multiplied by the original Z‐score sign (so that variables' Z‐scores were moved further from zero by the effect size shift, whether they were positive or negative). We then multiplied the shifted Z‐scores by standard errors to return to the observed effect scale. We applied the same shift to the true simulated effect sizes. PriorityFDR analysis was performed as for the other simulation analyses, and it does not attempt to model correlation structure.

Comparison against EBrank was performed using the 100 simulated mixtures of normal distributions, drawing simulated datasets of (a) 10,000 variables and (b) 1,000 variables from each of the 100 simulated mixture distributions. This was repeated using 100 mixtures of Laplacian distributions, by using the same $s_{k}$ values to produce Laplacian scale parameters $s_{k}/\sqrt{2}$. For computing the mean effect size rank among the largest set of variables deemed to have mean rank greater than a given quantile (final 3 columns in Table 1a,b), it was necessary to convert EBrank's estimated quantiles into tail‐area estimates. We computed these by sorting the variables by estimated rank and taking the mean of the extreme tail beyond the point represented by each variable, as we do for priorityFDR (see Supporting Information S1: Appendix S1).

### 4.2 T1DGC Data: Quality Control and Genome‐Wide Association Analysis

T1DGC family data were obtained through the T1DGC, which collected the families from Europe and the UK, North America, and the Asia‐Pacific region. Samples were genotyped on Illumina Human Core Exome beadchip following manufacturer protocols and genotype clusters were generated using the Illumina GeneTrain2 algorithm at University of Virginia. Since multiple array versions were used, we harmonized genetic variants across array versions in the following way: for those SNP variants that are available on the 1000 Genomes Project SNP panel, we align them to the 1000 Genomes Project SNPs separately for each array version; for those SNPs that are not available on the 1000 Genomes Project SNP panel, we harmonize them according to their positions/names, allele labels and allele frequencies that are specific to each of the four array versions.

Sample identity was confirmed by comparing genotypes from the same samples generated with an alternative array (Robertson et al. 2021) (Immunochip). Variants were removed for the following reasons: (a) more than 5% of genotypes were missing, (b) genotypes were inconsistent across duplicates (discordant in > 1% of duplicate samples or monozygotic twins), (c) genotype frequencies deviated from Hardy–Weinberg Equilibrium (*p* ≤ 10^−6^), (d) Mendelian inconsistencies in more than 1% of trios or parent‐offspring pairs or (e) more than 10% of homozygous parent‐offspring pairs or trios with heterozygous offspring. Samples were removed if more than 5% of genotypes were missing or genotypes were inconsistent with reported sex. Sample pedigree information was confirmed or corrected using genotype‐inferred relationships, as determined using the software KING (Manichaikul et al. 2010). After QC, there were 3173 affected‐offspring trios.

Subjects with European ancestry were identified for analysis using KING. Specifically, genetic principal components (PCs) were generated for 1000 Genomes Phase 3 subjects and T1DGC subjects were projected onto this PC space. Then a Support Vector Machine was used to classify T1DGC subjects into one of five ancestral super‐populations, as described here: https://www.kingrelatedness.com/manual.shtml.

European individuals (*n* = 10,406) were aligned to the Haplotype Reference Consortium (HRC) reference panel using available tools (https://www.well.ox.ac.uk/~wrayner/tools/index.html#Checking) and imputed to the HRC using the Michigan Imputation Server. Imputed variants were filtered for imputation quality (removed variants with imputation R‐squared < 0.3) and Mendelian errors (removed variants with errors in > 1% of homozygous parent‐offspring pairs or trios with heterozygous offspring). ORs were derived as OR = T/U, where T and U are the numbers of transmitted and non‐transmitted alleles, and SEs of log ORs for inverse‐variance weighting were obtained following Kazeem and Farrall (Kazeem and Farrall 2005). To prevent extreme ORs (e.g. zero or infinity), we added 0.5 to both T and U for a given variant whenever either value was 5 or lower.

### 4.3 UK Case Control T1D Samples: Quality Control and Genome‐Wide Association Analysis

T1D summary statistics were generated using GWAS data from 13,245 UK individuals in two sets of case control samples: 7977 genotyped using the Illumina Infinium 550K platform (3983 cases and 3994 controls) and 5268 using the Affymetrix GeneChip 500K platform (1926 cases and 3342 controls), analysed in previous publications (Cooper et al. 2017; Inshaw et al. 2021).

Genotypes were imputed using the HRC haplotypes and the Michigan Imputation server, pre‐phasing using SHAPEIT2 and imputing using Minimac3 (Das et al. 2016). Variants failing either of two imputation quality criteria in either UK cohort were removed: (a) imputation information score of < 60% in either cases or controls, or (b) difference in imputation information score between cases and controls > 1% together with MAF < 5%. Exceptions were made for two well‐established T1D variants in the *INS‐IGF2* region (rs689 and rs3842753), which were poorly imputed in the Affymetrix cohort but well imputed in the Illumina cohort. GWAS summary statistics were produced using the ‘newml’ method from SNPTEST, including the three largest PC covariates. Variants were LD pruned (*r* ^2^ < 0.3) and low (< 1%) MAF variants were removed during calculation of PCs. PCs were calculated within UK Affymetrix and Illumina collections separately.

### 4.4 GWAS Meta‐Analysis of Five Type 1 Diabetes Cohorts

Summary statistics for the two UK cohorts and T1DGC were meta‐analysed, together with a Sardinian cohort (Inshaw et al. 2021) (1558 cases and 2882 controls, genotyped on Affymetrix 6.0 and Illumina Omni Express), imputed from a custom reference panel of 3514 Sardinians, and samples from the FinnGen biobank resource (data freeze 4, phenotype code E4_DM1, *n* = 4933 cases and 148,190 controls). FinnGen test statistics correlated well with the test statistics obtained using strictly defined T1D cases (Supporting Information S2: Figure S13). Variants with MAF < 0.5% in either UK cohort or in the Sardinian cohort were removed. Variants not present in at least one of the UK cohorts were removed, as statistical power was likely to be low. As the HLA region is already well established as being T1D‐associated, and has extensive LD which may interfere with downstream analysis, we removed this as standard (40,656 variants with build 37 positions 25‐35 Mb on chromosome 6), leaving 6,254,180 variants.

Combined estimates of effect size from the five cohorts were obtained using inverse‐variance weighting, in R:

$${\hat{\beta }}_{\text{meta}}=\frac{\sum_{i=1}^{5}{\hat{\beta }}_{i}/{\hat{\sigma }}_{i}^{2}}{\sum_{i=1}^{5}1/{\hat{\sigma }}_{i}^{2}},$$

where ${\hat{\beta }}_{i}$ is the estimate of the log OR for the *i*th cohort and ${\hat{\sigma }}_{i}$ its estimated SE, which are set to zero and infinity respectively when the variant is missing in cohort *i*. *p* values were computed from the meta‐analysis Chi‐square statistics ${\hat{\beta }}_{\text{meta}}^{2}/{\hat{\sigma }}_{\text{meta}}^{2}$ (1 degree of freedom), where ${\hat{\sigma }}_{\text{meta}}^{2}$ is the variance of ${\hat{\beta }}_{\text{meta}}$:

$${\hat{\sigma }}_{\text{meta}}^{2}=\frac{1}{\sum_{i=1}^{5}1/{\hat{\sigma }}_{i}^{2}}.$$

The square root of ${\hat{\sigma }}_{\text{meta}}^{2}$ is the meta‐analysis standard error which, together with ${\hat{\beta }}_{\text{meta}}$, for each variable, constitutes the input data for priorsplitteR priorityFDR estimation.

### 4.5 Definition of Signals

PriorityFDR analysis was run on our T1D GWAS summary statistics (effect estimates and standard errors) using non‐HLA variants, providing tail‐area estimates of both FDR and priorityFDR (see Appendix for details on priorityFDR estimation: Supporting Information S1). Although it would be possible to use the popular Benjamini‐Hochberg procedure (Benjamini and Hochberg 1995) to calculate FDRs, we use the priorityFDR software to maintain a consistent model of variants' effects. Our significance criterion was FDR ≤ 1%. To define independent signals among the significant variants, we selected the most significant variant (lowest *p* value) on each chromosome, designating this as the lead variant for the signal, before removing any significant variant in LD (*r* ^2^ > 0.01, up to maximum 1 Mb distance), then choosing the next most significant variant remaining as the lead variant for the following signal. This process (‘LD clumping’) was repeated until no variants pass our significance criteria remained. Owing to the general ubiquity of LD, single‐variant associations, i.e. those that have no disease‐associated LD partners, are likely to be spurious due to problems with genotyping or imputation, especially when MAF is low. At each step, we therefore excluded the most significant variant from the process if it had no LD partners (*r* ^2^ > 0.1) with log10 *p* values lower than log10(P)/3‐1, where *p* is the lead variant's *p* value. LD calculations were performed in plink (Purcell et al. 2007) using 381,380 individuals from UK Biobank (UKBB) (Bycroft et al. 2018), after restricting to white‐Europeans and removing first and second‐degree relatives (using data field 22006). LD comparisons were restricted to a sliding window of 1 Mb. We designated GWAS signals as ‘new’ if they had *r* ^2^ < 0.05 with, and were physically located at least 250 kb from, the lead variants from any established regions (Barrett et al. 2009; Chiou et al. 2021; Onengut‐Gumuscu et al. 2015; Robertson et al. 2021) (*r* ^2^ calculations made using the UKBB LD data).

### 4.6 Annotation of Lead Variants

Functional annotations for each lead variant were obtained using the biomaRt R package (Ensembl build 38 human SNP database). We also wrote command‐line GraphQL and R scripts to download and filter lists of immune pheWAS associations (*p* ≤ 5 × 10^−4^) from the Open Targets Genetics portal (Ghoussaini et al. 2021) (https://genetics.opentargets.org), for each lead variant. The diseases/traits we filtered for were hypothyroidism, Addison's disease, asthma, coeliac disease, Crohn's disease, eczema, hayfever, lupus, multiple sclerosis, psoriasis, rheumatoid arthritis, ulcerative colitis, vitiligo, thrombocytopenia, monocyte count and basophil percentage.

### 4.7 Stepwise Regression and Fine‐Mapping

Stepwise model selection was performed on our T1D GWAS summary statistics and LD data from UKBB white Europeans (*n* = 381,380), before joint regression analysis of the selected variants, using COJO (Yang et al. 2012). Variants were incorporated into the model if they had a stepwise *p* value either (a) lower than genome‐wide significance (*P *≤ 5 × 10^−8^) for signals where the lead variant was genome‐wide significant or (b) lower than the GWAS *p* value for the lead variant for signals where this was greater than 5 × 10^−8^. Signals were defined as the set of variants within 250 kb of each lead variant, but not in LD with any more significant lead variants (r^2^ > 0.01). Variants previously determined to be single‐variant associations were omitted, as described above. The meta‐analysis sample size, for each variant, was calculated as the summed sample sizes of the four case‐control cohorts plus half the number of informative allele transmissions in the T1DGC TDT test. Fine‐mapping for each signal was performed with FINEMAP version 1.4 (Benner et al. 2016), within 500 kb windows around the lead variant, using LD data from UKBB (381,380 white Europeans). Analysis was restricted to the largest UK meta‐analysis cohort only (UK Illumina 550K, 3983 cases and 3994 controls), to ensure homogeneity of genotyping coverage.

### 4.8 Mendelian Randomisation Analysis of eQTL and T1D GWAS Data

Combining whole‐blood gene eQTL data for 19,942 genes from eQTLgen (*n* = 31,684) (Võsa et al. 2021) with our T1D GWAS meta‐analysis data, we performed MR with gene expression levels as exposures and T1D status as the outcome, using the TwoSampleMR R package (Hemani et al. 2018). Only variants with *p* < 5 × 10^−8^ in eQTLgen were included as instruments, leaving 13,499 genes with at least one instrument. Variants were LD‐pruned to exclude those with *r* ^2^ > 0.001, within a distance of 10MB. MR was performed using inverse variance weighting for genes with more than one genetic variant instrument, or with the Wald ratio otherwise. Ensembl Biomart queries for identifying MR significant genes (and IL‐2 pathway genes) lying close to our lead variants were performed using the biomaRt R package.

## Author Contributions

The project was conceived by John A. Todd, Stephen S. Rich and Daniel J.M. Crouch. Genotype data processing, quality control, imputation, and statistical analyses were performed by Daniel J.M. Crouch, Jamie R.J. Inshaw, Catherine C. Robertson, Esther Ng, Jia‐Yuan Zhang, Wei‐Min Chen, Suna Onengut‐Gumuscu, and Carlo Sidore. New statistical methods were designed and tested by Daniel J.M. Crouch. T1DGC DNA samples for genotyping were managed by Suna Onengut‐Gumuscu Carlo Sidore and Francesco Cucca contributed genotype data for the Sardinian cohort. Flemming Pociot and Patrick Concannon provided samples for genotyping through their affiliated institutions and research programs. Biological interpretation of results and IL‐2 pathway genes were provided by Antony J. Cutler and John A. Todd. The manuscript was written by Daniel J.M. Crouch and John A. Todd with technical input from Jamie R.J. Inshaw and Catherine C. Robertson.

## Conflicts of Interest

John A. Todd consults for GSK, Precion, Avammune, Immunocore and Vesalius Therapeutics. The other authors declare no conflicts of interest.

## Supporting information

Supporting information.

Supporting information.

Supporting information.

Supporting information.

## Data Availability

Type 1 diabetes GWAS meta‐analysis summary statistics generated during the study are available from GWAS Catalog (https://www.ebi.ac.uk/gwas, study accession GCST90013791), and have been incorporated into the Open Targets Genetics Portal (https://genetics.opentargets.org, same accession number). The R package for priorityFDR estimation, priorsplitteR, is available from https://github.com/djmcrouch/priorsplitteR.
