# Supplementary material for: Bayesian Effect Size Ranking to Prioritise Genetic Risk Variants in Common Diseases for Follow‐Up Studies
Source: Genet Epidemiol. 2025 Jan 3;49(1):e22608. doi: 10.1002/gepi.22608 (PMC11696485; doi:10.1002/gepi.22608)
Supplement: Supplementary file 1 — Supporting information. [file GEPI-49-0-s002.pdf]

PriorsplitteR method for priorityFDR estimation

Appendix to:

Bayesian effect size ranking to prioritise genetic risk variants in common diseases for follow-up studies

Daniel J. M. Crouch Jamie R.J. Inshaw Catherine C. Robertson Esther Ng
Jia-Yuan Zhang Wei-Min Chen Suna Onengut-Gumuscu Antony J. Cutler
Carlo Sidore Francesco Cucca Flemming Pociot Patrick Concannon
Stephen S. Rich John A. Todd

See main text for author affiliations.

Contents

1 Overview **2**

2 Estimating priorityFDRs **4**

Summary 4
Stage 1: Estimate the parameters of ANE 6
Stage 2: Simulate alt effect estimates and estimate the NPE parameters 9
Stage 3: Estimate the parameters of NPE-diff models 11
Stage 4: Compute fdrs 13
Stage 5: Compute ep estimates 14
Stage 6: Estimate tail area FDRs, EPs and priorityFDRs 16

3 Prior splitting theory **17**

Splitting priors into non-negative and non-positive mixtures 17
Splitting from the zero-effect distribution 19

1 Overview

The priorityFDR is a metric for controlling the overall cost of following up variables based on association data from a family of tests, based on a) the probability that the variables are a null (have zero true effect), and b) the probability that they are non-null, but with effects that are insubstantial compared to other variables from the same family of tests. Our motivation for developing the priorityFDR came from observing the many variables that appear to be statistical outliers on the x-axis of a volcano plot, representing effect size, have modest levels of significance, shown on the y-axis. Variables may therefore be interesting despite not passing standard significance criteria, or relatively uninteresting despite having high significance. The priorityFDR can also be viewed as an estimate of the rank of the true effect size, of a given focal variable, among the non-null effect sizes in the same dataset. Once estimated, it can easily be used to compute an estimate of the inclusive priorityFDR (priorityFDR$^{(\text{inc})}$), which measures the effect size rank among all variables in the dataset, both null and non-null (see Equation 5 in the main text). The priorityFDR and priorityFDR$^{(\text{inc})}$ are related quantities providing answers to slightly different questions. Researchers who wish to rank effect sizes among a set that has already been selected for statistical significance are most likely to find the priorityFDR useful, as it may be assumed that most of these are non-null. Alternatively, the priorityFDR$^{(\text{inc})}$ is most suited to prioritising among all available variables without applying a significance threshold. The two measures may also show different dynamics with increasing sample size, as power to distinguish increasingly small effects from null status will affect them in different ways, as discussed in the main text.

The method we introduce for estimating priorityFDRs in the following sections can be applied to any kind of multivariate data e.g. associations between genetic variants and phenotypes, or protein or mRNA gene expression measurements, provided these are normally distributed under the null hypothesis of no true effect. Individual level data are not required, only effect estimates and standard errors for each variable. All variables are treated as having the same underlying prior distribution of true effect sizes, so some consideration should be given as to whether this is indeed the case, or whether there are sets of variables that ought to be analysed separately if they are expected to have different prior distributions.

See main text Equations 1-4 for an informal definition of the priorityFDR. When capitalised, we use priorityFDR$_i$ to refer to a tail-area quantity, like a P-value, pertaining to variables with results more extreme than variable i:

$$\text{priorityFDR}_i = \text{FDR}_i + \text{EP}_i \times (1 - \text{FDR}_i), \tag{1}$$

$$\text{FDR}_i = \Pr(H_0 = 1 \big| \text{fdr} \leqslant \text{fdr}_i, \text{ep} \leqslant \text{ep}_i), \tag{2}$$

$$\text{EP}_i = \Pr(|\beta_{\text{alt}}| \geqslant |\beta| \big| \text{fdr} \leqslant \text{fdr}_i, \text{ep} \leqslant \text{ep}_i, H_0 = 0), \tag{3}$$

where EP refers to 'effect priority', β is a variable's risk effect, β_{alt} is a second variable's effect drawn from the prior distribution of alternative (alt, i.e. non-null) effect sizes, and H_0 is a binary indicator variable taking the value 1 when the null hypothesis is true and 0 when false. Following a general convention, we use the lower-case fdr and ep to refer to 'local' versions of the FDR and EP. These are defined as:

$$\text{fdr}_j = \Pr(H_{0(j)} = 1 \big| z_j), \tag{4}$$

$$\text{ep}_j = \Pr(|\beta_{\text{alt}}| \geqslant |\beta_j| \big| \hat{\beta}_j, \hat{\sigma}_j, H_{0(j)} = 0), \tag{5}$$

where z_j, $\hat{\beta}_j$ and $\hat{\sigma}_j$ are the observed Z-score, estimated effect size and estimated standard error for variable j, and $H_{0(j)}$ is the null hypothesis indicator variable for variable j. Our method for estimating priorityFDRs, described in the following sections, is to first estimate ep_j and fdr_j for $j = 1, 2, ...N$, where N is the total number of variables analysed, then to empirically integrate them across the tail area to obtain EP_i and FDR_i. As the method relies on empirical Bayesian reasoning in which it is assumed that all variables share the same prior distribution of risk effects, N should be large, probably at least 1000, in order to obtain good priorityFDRs estimates. If there is reason to believe that there is known subset of variables, e.g. SNPs in the HLA region, which is believed to share a markedly different prior effect distribution to the majority, we advise excluding this.

The first term in Equation 1 is the posterior probability that variables passing a bivariate threshold defined by variable i are false positives, while the second term, which can also be rewritten as

$$\text{EP}_i \times (1 - \text{FDR}_i) = \Pr(|\beta_{\text{alt}}| \geqslant |\beta| \cap H_0 = 0 \big| \text{fdr} \leqslant \text{fdr}_i, \text{ep} \leqslant \text{ep}_i), \tag{6}$$

is the probability that they are non-null, but have effect sizes that are exceeded by a randomly chosen non-null variables. Using the fact that the events

$$\{H_0 = 1\},\ \{|\beta_{\text{alt}}| \geqslant |\beta| \cap H_0 = 0\} \text{ and } \{|\beta_{\text{alt}}| < |\beta| \cap H_0 = 0\}, \tag{7}$$

have a total probability of 1, the usefulness of the priorityFDR can be further appreciated by subtracting it from 1:

$$1 - \text{priorityFDR}_i = \Pr(|\beta_{\text{alt}}| < |\beta| \cap H_0 = 0 \big| \text{fdr} \leqslant \text{fdr}_i, \text{ep} \leqslant \text{ep}_i), \tag{8}$$

which is the posterior probability that a variable in the tail-area is a true positive ($H_0 = 0$) and has an effect size greater than a randomly chosen alt (non-null) variable ($|\beta_{\text{alt}}| < |\beta|$), equivalent to its ranking among non-null variables where 1 is the largest effect and 0 the smallest. Correspondingly, the priorityFDR estimates 1 minus the rank of the true affect among non-null variables (see also main text).

When computing the tail-area priorityFDR in Equation 1, we assume for simplicity that FDR_i only depends on the variables with $\text{fdr} \leqslant \text{fdr}_i$, and EP_i only depends on variables with $\text{ep} \leqslant \text{ep}_i$:

$$\Pr(H_0 = 1 \big| \text{fdr} \leqslant \text{fdr}_i, \text{ep} \leqslant \text{ep}_i) \equiv \Pr(H_0 = 1 \big| \text{fdr} \leqslant \text{fdr}_i), \tag{9}$$

$$\Pr(|\beta_{\text{alt}}| \geqslant |\beta| \big| \text{fdr} \leqslant \text{fdr}_i, \text{ep} \leqslant \text{ep}_i, H_0 = 0) \equiv \Pr(|\beta_{\text{alt}}| \geqslant |\beta| \big| \text{ep} \leqslant \text{ep}_i, H_0 = 0). \tag{10}$$

2 Estimating priorityFDRs

Summary

In essence, the method we present uses polynomial models to fit the distribution of standardised variable effect estimates (Z-scores) as a two-mixture model of null and alternative effects. Further polynomials are then used to fit a two-mixtures model for the non-null Z-scores: a 'negative' mixture with true effects below zero and a 'positive' mixture with true effects above zero. We abbreviate these two mixture models as ANE (alt/null effects) and NPE (negative/positive effects). To simplify computation, we opt to fit and then fix the parameters of the ANE model before fitting the NPE model, rather than fitting both mixture models simultaneously, despite NPE depending on the parameters of ANE. Fitting the NPE model involves a novel technique we call 'prior splitting', described in Section 3, which is the major statistical innovation in our approach to priorityFDR estimation.

We use the fitted ANE distribution together with Bayes' theorem to find fdr_j for $j = 1, 2, ...N$. Fitting another NPE distribution, this time to randomly chosen pairwise effect estimate differences ('NPE-diff'), Bayes' theorem is reapplied to find the posterior probabilities that variables $j = 1, 2, ...N$ have effects larger or equal to those for large random selections of variables $k_j \in \{1, 2, ...N\}$, so that averaging over k_j estimates ep_j for each j in a computationally tractable way, avoiding comparisons between all pairwise variable combinations. Tail-area estimates are then obtained from fdr_j and ep_j $\{\forall j \in 1, 2, ...N\}$ as overviewed in the previous section. The method, which uses summary statistics rather than individual-level genotype data, is implemented in R and is available in the package `priorsplitteR` (https://github.com/djmcrouch/priorsplitteR).

The ANE and NPE models are estimated using polynomial likelihood models for the observed variable effect estimate data, so our method falls within the f-modelling category of empirical Bayesian approaches, in contrast with g-modelling which estimates the prior explicitly. An advantage of f-modelling is that the observed data can be fit directly without having to estimate a prior distribution, which can be challenging as different priors can produce similar observed data [Efron and Hastie, 2016].

The stages of priorityFDR estimation are:

1) Estimate the parameters of ANE, including choosing the degree of the polynomial likelihood function using the AIC.

2) Simulate effect estimates from the estimated alt distribution using the `distr` R package. Estimate NPE parameters using the simulated effect estimate dataset.

3) Simulate two sets of effect estimates, one from the NPE mixture corresponding to variables with positive effects, and one from the mixture corresponding to negative effects. Take the difference between each of these and the alt simulation from Stage 2, and fit models of to both sets of differences using the NPE method (NPE-diff) as a means of estimating $\Pr(\beta_k > \beta_j | \beta_j > 0, \hat{\beta}_j, \hat{\sigma}_j, \hat{\beta}_k, \hat{\sigma}_k, H_{0(j)} = 0, H_{0(k)} = 0)$ and $\Pr(\beta_k < \beta_j | \beta_j < 0, \hat{\beta}_j, \hat{\sigma}_j, \hat{\beta}_k, \hat{\sigma}_k, H_{0(j)} = 0, H_{0(k)} = 0)$.

4) Compute fdr_j for each variable $j = 1, 2, ...N$ using the fitted ANE model

5) Compute ep_j for each variable $j = 1, 2, ...N$ using the fitted NPE-diff models, by averaging $\Pr(\beta_k > \beta_j|\beta_j > 0, \hat{\beta}_j, \hat{\sigma}_j, \hat{\beta}_k, \hat{\sigma}_k, H_{0(j)} = 0, H_{0(k)} = 0)$ and $\Pr(\beta_k < \beta_j|\beta_j < 0, \hat{\beta}_j, \hat{\sigma}_j, \hat{\beta}_k, \hat{\sigma}_k, H_{0(j)} = 0, H_{0(k)} = 0)$ over a set of alt-distribution variables indexed by k.

6) Estimate local and tail-area FDR_i and EP_i using estimates of fdr_j and ep_j, and the approach outlined in the previous section.

Stage 1: Estimate the parameters of ANE

In the current version of `priorsplitteR`, we assume that optimal ANE model fitting can be achieved using z-scores ($z_j = \hat{\beta}_j/\hat{\sigma}_j$ for variable j), without considering the effect sizes and standard errors separately, i.e. that variables with different standard errors have similar z-score distributions[1]. Simulation experiments demonstrated that the method is robust to violations of this assumption (see main text Figure 1).

We estimate distributions using an implementation of Lindsey's Poisson regression method ([Lindsey, 1974a, Lindsey, 1974b]). As if producing a histogram, imagine discretising the z-scores into B equal-width bins $b = 1, 2...B$ (with equal widths chosen by the Freedman-Diaconis Rule), treating the number of variables falling into each bin as a random Poisson variable. Thus, the likelihood for the number of variables (the count, c_b) falling into bin b is

$$\frac{(N\lambda(z_b))^{c_b} e^{-N\lambda(z_b)}}{!c_b}, \tag{11}$$

where $\lambda(z_b)$ approximates the probability density function of z within the region spanned by bin b as a function of its central point z_b, and $N\lambda(z_b)$ is the Poisson parameter for bin b. As described in Efron and Hastie ([Efron and Hastie, 2016]), one could use polynomial model for $\ln\lambda(z_b)$:

$$\lambda(z_b) = e^{\sum_{n=0}^{D} \gamma_n z_b^n}, \tag{12}$$

where D is the degree of the polynomial[2]. Extending Lindsey's method, we use two λ_b variables for each bin, representing the alternative and null mixture components:

[1]Before fitting the ANE model in Stage 1, we remove small numbers of outlying variables (by default as those with $|z_j| > 12$, or the 99.9% quantile of absolute Z-scores, if the latter is larger, up to a maximum of 20), as these can make model fitting unnecessarily difficult. The remaining variables are sorted into a number of z-score bins of equal width, spanning the range of z-scores. Bin width is chosen using the Freedman-Diaconis Rule: $2 \times IQR \times N^{-1/3}$, where IQR is the interquartile range of the z-scores

[2]Scaling is applied to z_b^n so that SD=1 across b for each n, equivalent to scaling each γ_n, to simplify numerical optimisation.

$$\lambda_1(z_b) = we^{\sum_{n=0}^{D} \gamma_n z_b^n}, \tag{13}$$

$$\lambda_0(z_b) = w\phi(z_b), \tag{14}$$

where $\phi(\cdot)$ is the standard normal distribution function, γ_n is a model parameter and w the width of the bins[3]. The likelihood for the count in bin b is

$$L_b(c_b) = \frac{\big(N\pi\lambda_0(z_b) + N(1-\pi)\lambda_1(z_b)\big)^{c_b} e^{-N\left(\pi\lambda_0(z_b)+(1-\pi)\lambda_1(z_b)\right)}}{!c_b}, \tag{15}$$

where π is the mixing proportion, also the prior probability of a variable belonging to the null distribution of zero effect. To obtain maximum-likelihood estimates of the parameters π and $\boldsymbol{\gamma} = [\gamma_1, \gamma_2, ...\gamma_D]$, we maximise the overall log-likelihood $\sum_{b=1}^{B} \ln L_b(c_b)$. Rather than maximising directly, which is challenging for mixture models, we employ an EM-algorithm [Dempster et al., 1977], treating the expected proportion of counts belonging to the null distribution within each bin, conditioned on the current parameter choices $\theta = \{\pi, \boldsymbol{\gamma}\}$, as an unknown latent variable:

$$m_b = \frac{\lambda_0(z_b)\pi}{\lambda_0(z_b)\pi + \lambda_1(z_b)(1-\pi)}, \tag{16}$$

where the expected proportion belonging to the alt distribution is $1 - m$. Using this equation to compute m_b based on θ (the E-step), we maximise (in the M-step) $Q(\theta') = \sum_{b=1}^{B} \big(f_0(c_b, m_b, z_b) + f_1(c_b, m_b, z_b)\big)$, where

$$f_1(c_b, m_b, z_b) = c_b(1-m_b)(\ln(1-\pi') + \ln\lambda_1'(z_b)) - (1-\pi')\lambda_1'(z_b)N, \tag{17}$$

$$f_0(c_b, m_b, z_b) = c_b m_b \ln\pi' - \pi'\lambda_0(z_b)N, \tag{18}$$

with respect to $\theta' = \{\pi', \boldsymbol{\gamma}'\}$, and $\lambda_1'(z_b)$ is Equation 13 parameterised by $\boldsymbol{\gamma}'$[4]. Maximisation is performed with the Broyden-Fletcher-Goldfarb-Shanno (BFGS) algorithm [Broyden, 1970, Fletcher, 1970, Goldfarb, 1970, Shanno, 1970] implemented in the `constrOptim` R function, using the first partial derivatives w.r.t. each parameter, which we derived analytical expressions for. The BFGS algorithm requires an initialisation point (described below), from which

[3] In practice, we use the cumulative normal distribution to improve the approximation $w\phi(z_b)$ (and for the equivalent null distribution in the NPE model), by computing the probability mass lying within the bin, but this is not essential.

[4] $\lambda_0(z_b)$ contains no parameters.

it explores the parameter space using the $Q(\theta')$ values and derivatives until a convergence threshold is exceeded. The π' parameter is constrained to be within $[0,1]$[5].

Equations 17 and 18 are derived from the expected value of the log-likelihood $\sum_{b=1}^{B}(h_0\big(c_b, M_b, z_b)+h_1(c_b, M_b, z_b)\big)$, where

$$h_1(c_b, M_b, z_b) = c_b(1-M_b)(\ln(1-\pi') + \ln\lambda'_1(z_b) + \ln N) - (1-\pi')\lambda'_1(z_b)N - \ln!(c_b(1-M_b)), \tag{19}$$

$$h_0(c_b, M_b, z_b) = c_b M_b(\ln\pi' + \ln\lambda_0(z_b) + \ln N) - \pi'\lambda_0(z_b)N - \ln!(c_b M_b), \tag{20}$$

and M_b is a random variable, conditioned on π and $\lambda_1(z_b)$, giving the proportion of counts in bin b belonging to the null distribution under the previous parameter estimates. The expected value of M_b is m_b. In Equations 17 and 18 we ignore terms for which the equivalents in Equations 19 and 20 contain no parameters, as they remain constant under maximisation. This is particularly helpful as there are no closed-form expressions for expectations of the final terms $\ln!(c_b(1-M_b))$ and $\ln!(c_b M_b)$.

The M- and E-steps are iterated until convergence. In order to increase the log-likelihood using a generalised EM-algorithm, it is sufficient to find values of π' and $\lambda'_1(z_b)$ that increase (i.e. improve) the expected value of $Q(\theta')$, relative to $Q(\theta)$ [Hastie et al., 2009]. We therefore iterate until $Q(\theta')-Q(\theta)$ becomes stable and close to zero, with a default of less than 5×10^{-4} for three consecutive iterations[6]. The first M-step is initialised at an arbitrary point in the parameter space, but subsequent M-steps are initialised at the maximised parameter values obtained by the previous M-step ($\theta' \equiv \theta$) as, being generally close to the optimum, we found this provided the fastest and most reliable convergence.

Quality of the fit was improved by performing EM with different degrees of polynomial, D, for the alt distribution. By default we iterate D from 2 to 12, and selected the value producing the lowest AIC of the final likelihood fit after EM maximisation. Estimation of ANE using our type 1 diabetes (T1D) GWAS meta-analysis data produces the fitted models shown in Figure A1.

[5]We also aid fitting of ANE by constraining $\lambda_1(z_b) < \lambda_0(z_b)$ at $z_b = 0$, so as to avoid impossible distributions, by constraining $\gamma_0 < \ln w\phi(0)$.

[6]We also impose a default maximum of 10^5 iterations before accepting the parameter estimates.

Figure A1: Fitted ANE model for type 1 diabetes (T1D) GWAS meta-analysis data. Red, green and blue curves show the marginal mixture distribution, the alt mixture and null mixture respectively. In this example, each variable entered into the priorityFDR analysis is a SNP variant.

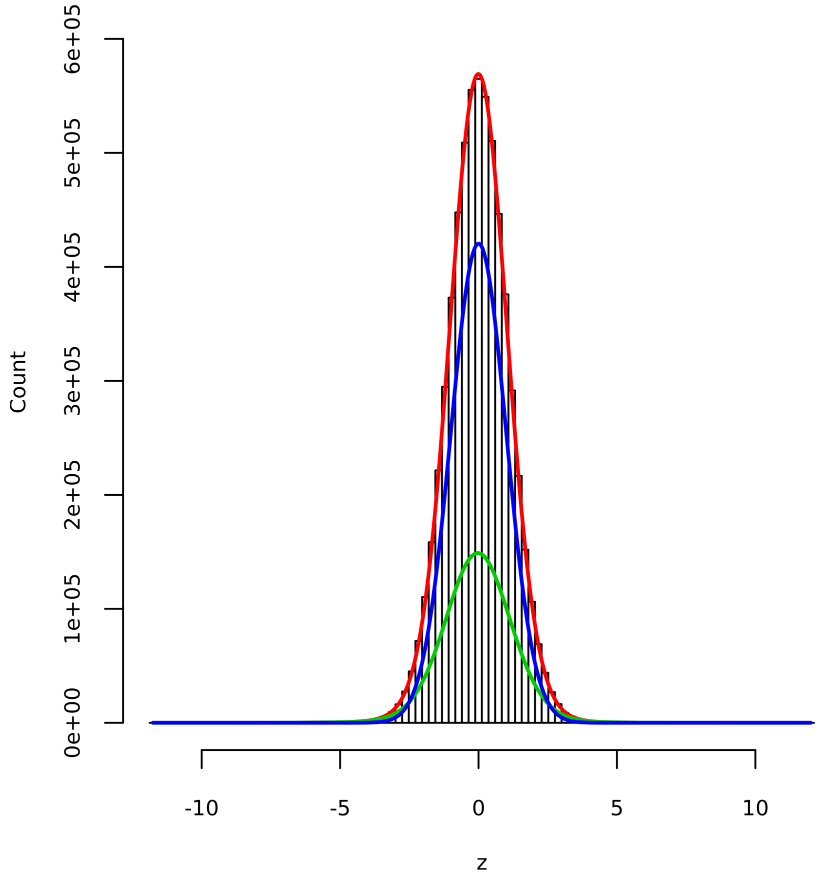

Stage 2: Simulate alt effect estimates and estimate the NPE parameters

Once the ANE model has been estimated, we fix its parameters at the maximum-likelihood estimates $\hat{\pi}$ and $\hat{\lambda}_1(z_b)$ (hat symbols throughout indicate maximum-likelihood estimates of the corresponding parameter) and proceed to estimate the parameters of NPE[7]. The NPE model decomposes the distribution of alt effect estimates into separate mixtures for those with true effects above zero and those with true effects below zero. As NPE is only concerned with non-null variables, we draw a large sample of z-scores, z_s for $s \equiv 1, 2, ...S$, from the fitted alt distribution defined by $\lambda_1(z_b)$ using the R package `distr`. Sufficiently many

[7] We take this approach for computational tractability, though in theory better estimates might be found by estimating both models simultaneously

samples should be drawn to avoid introducing significant levels of noise, so by default we set $S = 10^7$.

The purpose of the NPE model is to provide a basis for estimating the probability that a true effect β_s is positive or negative, given its corresponding z-score:

$$z_s = \frac{\beta_s}{\hat{\sigma}_s} + \epsilon_s, \tag{21}$$

where ϵ_s is a normal random variable with mean zero and unit variance. Though $\beta_s/\hat{\sigma}_s$ has an unknown distribution, we assume that it is drawn from the same prior distribution $g(\cdot)$ for all s. Rather than attempting to estimate $g(\cdot)$ explicitly, we use a novel technique (prior splitting) to split the distribution of z_s into a mixture of two distributions with non-overlapping priors, corresponding to the positive and negative effects. Following Lindsey's method as applied in Stage 1[8], the distributions of z-scores within each mixture respectively are:

$$\Lambda_{(+)}(z_b) = W\phi(z_b)e^{z_b \sum_{n=0}^{D}\sum_{p=0}^{D} \frac{\Gamma_n \Gamma_p z_b^n z_b^p}{n+p+1} + a}, \tag{22}$$

$$\Lambda_{(-)}(z_b) = W\phi(z_b)e^{-z_b \sum_{n=0}^{D}\sum_{p=0}^{D} \frac{\Gamma_n^* \Gamma_p^* z_b^n z_b^p}{n+p+1} + a^*}, \tag{23}$$

where z_b is the central point of bin b, W is the width of the bins, and Γ_n, Γ_p, a, Γ_n^*, Γ_p^* and a^* are model parameters, with a and a^* acting as intercept terms. Summation expressions within the exponents are similar to squared polynomials[9], and as such have a general flexibility that becomes greater as D is increased, similar to the polynomial used for ANE in Equation 13. However, derivatives of the ratios $\Lambda_{(+)}(z_b)/(W\phi(z_b))$ and $\Lambda_{(-)}(z_b)/(W\phi(z_b))$ are always non-negative and non-positive respectively. If $g_{(+)}(\cdot)$ and $g_{(-)}(\cdot)$ are the priors corresponding to distributions $\Lambda_{(+)}(z_b)$ and $\Lambda_{(-)}(z_b)$, we show in Section 3 that these constraints are equivalent to $g_{(-)}(x) = 0$ for $x \geqslant 0$ and $g_{(+)}(x) = 0$ for $x \leqslant 0$.

Estimation is performed using an EM-algorithm similar to Stage 1, where the expected proportions in each bin deriving from each mixture, conditioned on the current parameter estimates $\theta = \{\rho_{(+)}, \rho_{(-)}, \Lambda_{(+)}(z_b), \Lambda_{(-)}(z_b)\}$ are computed in the E-step as:

[8]By default setting D to half the value of D produced by ANE estimation after minimising the AIC, rounded upwards to the nearest integer, and using the same histogram bins as in ANE.

[9]Becoming squared polynomials after multiplyin each term by $n + p + 1$.

$$m_{b(+)} = \frac{\Lambda_{(+)}(z_b)\rho_{(+)}}{\Lambda_{(+)}(z_b)\rho_{(+)} + \Lambda_{(-)}(z_b)\rho_{(-)}}, \tag{24}$$

$$m_{b(-)} = 1 - m_{b(+)}, \tag{25}$$

where $\rho_{(+)}$ and $\rho_{(-)}$, summing to 1, are the mixing proportions for $\Lambda_{(+)}(z_b)$ and $\Lambda_{(-)}(z_b)$. In the M-step, similar to ANE, we maximise $Q(\theta') = \sum_{b=1}^{B}(f_{b(+)}(c_b) + f_{b(-)}(c_b))$, with respect to new parameters $\theta' = \{\rho'_{(+)}, \rho'_{(-)}, \mathbf{\Gamma} = \{\Gamma_1, \Gamma_2, ...\Gamma_D\}, \mathbf{\Gamma^*} = \{\Gamma^*_1, \Gamma^*_2, ...\Gamma^*_D\}, a, a^*\}$ where

$$f_{b(+)}(c_b) = c_b m_{b(+)}\big(\ln\rho'_{(+)} + \ln\Lambda'_{(+)}(z_b)\big) - \rho'_{(+)}\Lambda'_{(+)}(z_b)S, \tag{26}$$

$$f_{b(-)}(c_b) = c_b m_{b(-)}\big(\ln\rho'_{(-)} + \ln\Lambda'_{(-)}(z_b)\big) - \rho'_{(-)}\Lambda'_{(-)}(z_b)S, \tag{27}$$

and where c_b now represents the number of z_s falling into each bin b, with $\sum_{b=1}^{B} c_b = S$. As for ANE estimation, maximisation of $Q(\theta')$ is made easier using functions we wrote to compute its first partial derivatives, and non-linear optimisation R functions `alabama` and `constrOptim.nl` are used to search the parameter space for the optimum. Each M-step is initialised at $\theta' \equiv \theta$, as in ANE estimation, and we iterate E- and M-steps until the same convergence criteria are met[10]. Both $\rho'_{(+)}$ and $\rho'_{(-)}$ are constrained to be within $[0, 1]$ and sum to 1[11]. Equations 26-27 are derived from the expected log-likelihoods for each mixture distribution, ignoring terms containing no parameters, as in Stage 1. Estimates of NPE for the T1D GWAS meta-analysis data are shown in Figure A2.

Stage 3: Estimate the parameters of NPE-diff models

As a route towards modelling $\Pr(\beta_k > \beta_j | \beta_j > 0, \hat{\beta}_j, \hat{\sigma}_j, \hat{\beta}_k, \hat{\sigma}_k, H_{0(j)} = 0, H_{0(k)} = 1)$ and $\Pr(\beta_k < \beta_j | \beta_j < 0, \hat{\beta}_j, \hat{\sigma}_j, \hat{\beta}_k, \hat{\sigma}_k, H_{0(j)} = 0, H_{0(k)} = 1)$, we use simulated z-scores to define two sets of scaled effect estimate differences:

[10] By default convergence criteria are less strict then for ANE maximisation, as computation is more demanding for this part of the method. Estimates are accepted when the expected likelihood does not improve by more than 5×10^{-2} for 3 iterations, or a maximum of 1000 iterations is reached.

[11] Similar to ANE, we constrain a and a' to be negative with `alabama` and `constrOptim.nl`, thereby avoiding impossible distributions by ensuring $\Lambda_{(+)}(z_b)$ and $\Lambda_{(-)}(z_b)$ are less than $W\phi(z_b)$ at $z_b = 0$. To prevent potential maximisation problems arising from asymmetry in the model, we also constrain Γ_0 and $\Gamma^*_0 > 0$.. We further aid optimisation by applying constraints ensuring that, at each z_b, the value of the fitted likelihood for each mixture cannot be greater than $\phi(0)$, the null distribution at zero.

Figure A2: Fitted NPE model for T1D GWAS meta-analysis data. Green and turquoise curves are the fitted distributions for negative and positive true effects respectively, and the fitted marginal mixture distribution is red.

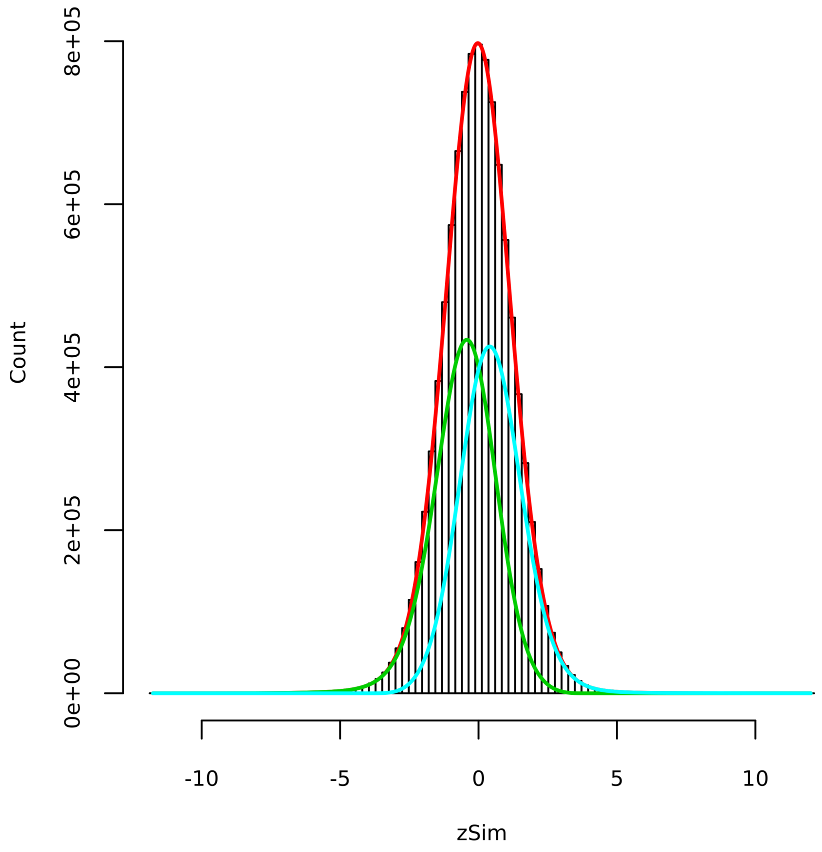

$$\delta_s = \frac{z_{s(1)}\hat{\sigma}_{s(1)} - z_{s(2)}\hat{\sigma}_{s(2)}}{\sqrt{\hat{\sigma}^2_{s(1)} + \hat{\sigma}^2_{s(2)}}}, \tag{28}$$

where $z_{s(1)}$ and $z_{s(2)}$ are simulated independently from the alt distribution in the same way as z_s (taking S samples), and the SEs $\hat{\sigma}_{s(1)}$, $\hat{\sigma}_{s(2)}$ are sampled independently from the empirical distribution of SEs according to their probabilities of belonging alt distribution[12]. As elements of δ_s are scaled differences between z-scores, we model them as

[12] S random samples are drawn from a multinomial distribution with N categories, where the probability of drawing each variable $i = 1, 2, ...N$ is $\frac{1}{N}\frac{\hat{\lambda}_1(z_i)(1-\hat{\pi})}{\hat{\lambda}(z_i)}$, in which hat symbols indicate estimated ANE parameters and N^{-1} is the empirical marginal distribution, which estimates an empirical version of the marginal distribution $\lambda(z_i)$.

$$\delta_s = \Delta_s + \epsilon_s, \tag{29}$$

where ϵ_s is normal distributed with mean zero and unit variance, and Δ_s has an unknown prior distribution similar to $g(\cdot)$ from the previous subsection. Applying the NPE estimation method again[13] to the scaled differences δ_s (NPE-diff) provides estimates of two mixture distributions conditioned on $\Delta_s > 0$, $\Delta_s < 0$:

$$p(\delta_s|\beta_k > \beta_j, H_{0(k)} = 0, H_{0(j)} = 0) \equiv \hat{\Lambda}^{(\text{diff})}_{(-)}(\delta_s), \tag{30}$$

$$p(\delta_s|\beta_k < \beta_j, H_{0(k)} = 0, H_{0(j)} = 0) \equiv \hat{\Lambda}^{(\text{diff})}_{(+)}(\delta_s), \tag{31}$$

which we assume, to avoid fitting further likelihood models, to well-approximate the likelihoods:

$$p(\delta_s|\beta_k > \beta_j, \beta_j > 0, \beta_k > 0) \text{ and} \tag{32}$$

$$p(\delta_s||\beta_k| > \beta_j, \beta_j > 0, \beta_k < 0), \tag{33}$$

and

$$p(\delta_s|\beta_k < \beta_j, \beta_j < 0, \beta_k < 0) \text{ and} \tag{34}$$

$$p(\delta_s|(-\beta_k) < \beta_j, \beta_j < 0, \beta_k > 0), \tag{35}$$

respectively. Estimated NPE-diff models are displayed in Figure A3. Estimation of this model also produces estimates of the mixing proportions $\hat{\rho}^{(\text{diff})}_{(+)}$ and $\hat{\rho}^{(\text{diff})}_{(-)}$, corresponding to $\hat{\Lambda}^{(\text{diff})}_{(+)}$ and $\hat{\Lambda}^{(\text{diff})}_{(-)}$ respectively. We use $\hat{\Lambda}^{(\text{diff})}(\delta_s)$ to refer to the marginal likelihood $\hat{\Lambda}^{(\text{diff})}_{(+)}(\delta_s)\hat{\rho}^{(\text{diff})}_{(+)} + \hat{\Lambda}^{(\text{diff})}_{(-)}(\delta_s)\hat{\rho}^{(\text{diff})}_{(-)}$.

Stage 4: Compute fdrs

We compute the estimate of fdr_j (see Equation 4) for each variable[14] j using Bayes' theorem and the ANE maximum-likelihood parameter estimates:

[13] Bins are reset for analysis of δ_s, using the same bin width as before, but we discard simulated differences with absolute values greater than 1.33 times the outlier threshold for the observed data, or the 99.99% quantile, whichever is larger.

[14] Z-scores lying outside the range of those used for fitting the distributions (due to removal of outliers) are set to the value of either the most negative or most positive z-score used, depending on their sign, as fits are likely to be unreliable outside of this range.

Figure A3: Fitted NPE-diff model for T1D GWAS meta-analysis data. Histograms show effect estimate differences between SNPs with positive true effects and randomly selected alt SNPs. Green and turquoise curves are the fitted distributions for negative and positive true effect differences respectively, and the fitted marginal mixture distribution is red.

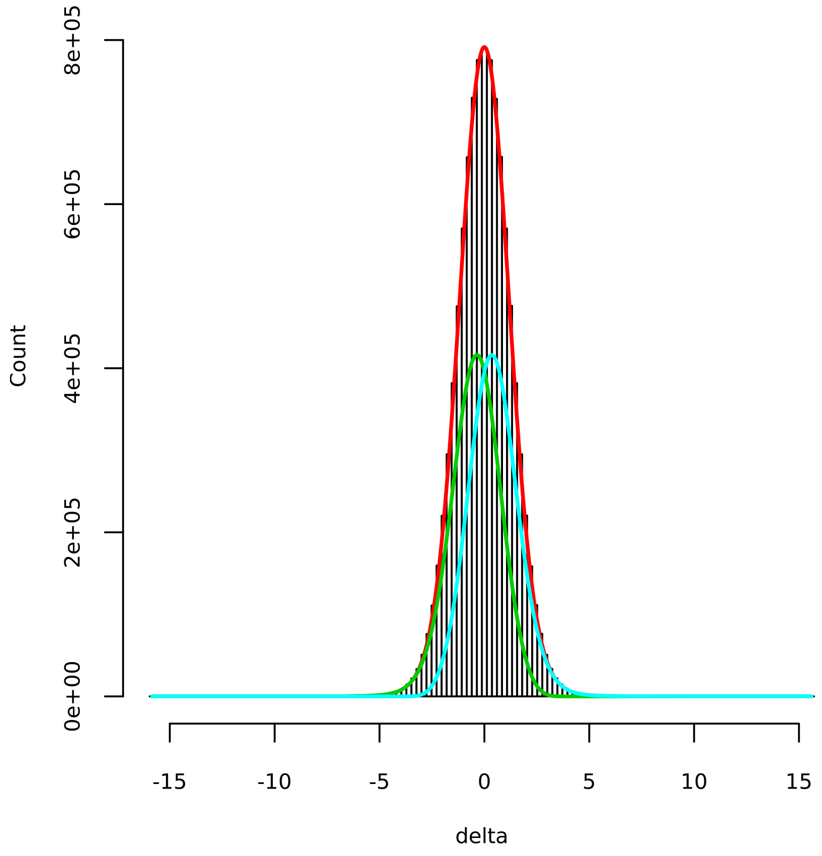

$$\widehat{\text{fdr}}_j = \frac{\hat{\lambda}_0(z_j)\hat{\pi}}{\hat{\lambda}_0(z_j)\hat{\pi} + \hat{\lambda}_1(z_j)(1-\hat{\pi})}. \tag{36}$$

Stage 5: Compute ep estimates

To estimate ep_j (Equation 5), a random selection of S variables is first drawn as if sampling from $\hat{\Lambda}_{(+)}$ and $\hat{\Lambda}_{(-)}$[15], before computing sets of effect differences[16] for each j, $\delta^{(\text{obs})}_{jk(+)}$ and $\delta^{(\text{obs})}_{jk(-)}$, where $k(+)$ and $k(-)$ indexes random subsets $t_j(+)$ and $t_j(-) \in \{1, 2, 3...S\}$ of the S simulated variables from $\hat{\Lambda}_{(+)}$ and $\hat{\Lambda}_{(-)}$, both with size $T \ll S$, to reduce computational

[15]Using the same method as for randomly selecting $\hat{\sigma}_s(1)$ and $\hat{\sigma}_s(2)$ in Stage 3.

[16]Suppressing outliers as in Stage 4.

resources involved in sampling S differences for each variable. Differences are computed as:

$$\delta^{(\text{obs})}_{jk(+)} = \frac{z_j\hat{\sigma}_j - \hat{x}_{k(+)}\hat{\sigma}_{k(+)}}{\hat{\sigma}_j}, \quad (37)$$

$$\delta^{(\text{obs})}_{jk(-)} = \frac{z_j\hat{\sigma}_j - \hat{x}_{k(-)}\hat{\sigma}_{k(-)}}{\hat{\sigma}_j}, \quad (38)$$

where $\hat{x}_{k(+)}$ and $\hat{x}_{k(-)}$ are the posterior expectations of the expected values of $z_{k(+)}$ and $z_{k(-)}$, estimated using Tweedie's method [Efron and Hastie, 2016, Robbins, 1956] applied to the positive-effect and negative-effect simulated datasets $\delta_{s(+)}$ and $\delta_{s(-)}$. Using posterior expectations instead of observed Z-scores means that $\delta^{(\text{obs})}_{jk(+)}$ and $\delta^{(\text{obs})}_{jk(-)}$ can be used to approximate the likelihood for z_j under the different mixture distributions, which is the observation to be conditioned upon, rather than a likelihood of a scaled difference between z_j and another Z-score. Reversed-sign differences are also computed, as:

$$\delta^{(\text{rev})}_{jk(+)} = \frac{z_j\hat{\sigma}_j + \hat{x}_{k(+)}\hat{\sigma}_{k(+)}}{\hat{\sigma}_j}, \quad (39)$$

$$\delta^{(\text{rev})}_{jk(-)} = \frac{z_j\hat{\sigma}_j + \hat{x}_{k(-)}\hat{\sigma}_{k(-)}}{\hat{\sigma}_j}. \quad (40)$$

We use the likelihoods in Expressions 30 and 31 to compute estimated posteriors

$$\widehat{\Pr}(\beta_k > \beta_j | \delta^{(\text{obs})}_{jk(+)}, \beta_j > 0, \beta_k > 0) = \frac{\hat{\Lambda}^{(\text{diff})}_{(-)}(\delta^{(\text{obs})}_{jk(+)})\hat{\rho}^{(\text{diff})}_{(-)}}{\hat{\Lambda}^{(\text{diff})}(\delta^{(\text{obs})}_{jk(+)})}, \quad (41)$$

$$\widehat{\Pr}(|\beta_k| > \beta_j | \delta^{(\text{rev})}_{jk(-)}, \beta_j > 0, \beta_k < 0) = \frac{\hat{\Lambda}^{(\text{diff})}_{(-)}(\delta^{(\text{rev})}_{jk(-)})\hat{\rho}^{(\text{diff})}_{(-)}}{\hat{\Lambda}^{(\text{diff})}(\delta^{(\text{rev})}_{jk(-)})}, \quad (42)$$

$$\widehat{\Pr}(\beta_k < \beta_j | \delta^{(\text{obs})}_{jk(-)}, \beta_j < 0, \beta_k < 0) = \frac{\hat{\Lambda}^{(\text{diff})}_{(+)}(\delta^{(\text{obs})}_{jk(-)})\hat{\rho}^{(\text{diff})}_{(+)}}{\hat{\Lambda}^{(\text{diff})}(\delta^{(\text{obs})}_{jk(-)})}, \quad (43)$$

$$\widehat{\Pr}((-\beta_k) < \beta_j | \delta^{(\text{rev})}_{jk(+)}, \beta_j < 0, \beta_k > 0) = \frac{\hat{\Lambda}^{(\text{diff})}_{(+)}(\delta^{(\text{rev})}_{jk(+)})\hat{\rho}^{(\text{diff})}_{(+)}}{\hat{\Lambda}^{(\text{diff})}(\delta^{(\text{rev})}_{jk(+)})}. \quad (44)$$

Averaging each over the selection indexed by k gives one-tailed ep estimates conditioned on either $\beta_j > 0$ or $\beta_j < 0$:

$$\widehat{\text{ep}}_j|\beta_j > 0, \beta_k > 0 = \frac{1}{T}\sum_{k \in t_j(+)} \widehat{\Pr}(\beta_k > \beta_j | \delta^{(\text{obs})}_{jk(+)}, \beta_j > 0, \beta_k > 0), \tag{45}$$

$$\widehat{\text{ep}}_j|\beta_j > 0, \beta_k < 0 = \frac{1}{T}\sum_{k \in t_j(-)} \widehat{\Pr}(|\beta_k| > \beta_j | \delta^{(\text{rev})}_{jk(-)}, \beta_j > 0, \beta_k < 0), \tag{46}$$

$$\widehat{\text{ep}}_j|\beta_j < 0, \beta_k < 0 = \frac{1}{T}\sum_{k \in t_j(+)} \widehat{\Pr}(\beta_k < \beta_j | \delta^{(\text{obs})}_{jk(-)}, \beta_j < 0, \beta_k < 0), \tag{47}$$

$$\widehat{\text{ep}}_j|\beta_j < 0, \beta_k > 0 = \frac{1}{T}\sum_{k \in t_j(-)} \widehat{\Pr}((-\beta_k) < \beta_j | \delta^{(\text{rev})}_{jk(+)}, \beta_j < 0, \beta_k > 0). \tag{48}$$

This stage may be parallelised across several thousand variables simultaneously[17]. By default we take a sample of variables that is not too large ($T \equiv 1000$), to save computation time. However, variables with low initial ep estimates (default below 0.05) in Equations 45-48 are sampled with more variables (default $T \equiv 10000$) to ensure that estimates are accurate for potentially interesting variables.

The overall two-tailed ep estimate is produced by summing over and weighting by the posterior probabilities that β_j and β_k are positive or negative, obtained from the NPE mixture distribution estimates $\hat{\Lambda}_{(+)}$ and $\hat{\Lambda}_{(-)}$, with estimated marginal $\hat{\Lambda}(z_j) = \hat{\Lambda}_{(+)}(z_j)\hat{\rho}_{(+)} + \hat{\Lambda}_{(-)}(z_j)\hat{\rho}_{(-)}$:

$$\widehat{\text{ep}}_j = \frac{\hat{\Lambda}_{(+)}(z_j)\hat{\rho}_{(+)}}{\hat{\Lambda}(z_j)}\big((\widehat{\text{ep}}_j|\beta_j > 0, \beta_k > 0)\hat{\rho}_{(+)} + (\widehat{\text{ep}}_j|\beta_j > 0, \beta_k < 0)\hat{\rho}_{(-)}\big) \tag{49}$$

$$+ \frac{\hat{\Lambda}_{(-)}(z_j)\hat{\rho}_{(-)}}{\hat{\Lambda}(z_j)}\big((\widehat{\text{ep}}_j|\beta_j < 0, \beta_k > 0)\hat{\rho}_{(+)} + (\widehat{\text{ep}}_j|\beta_j < 0, \beta_k < 0)\hat{\rho}_{(-)}\big), \tag{50}$$

where the probabilities of randomly chosen null variables having positive or negative true signs are given by the NPE prior probability estimates $\hat{\rho}_{(+)}$ and $\hat{\rho}_{(-)}$ respectively.

Stage 6: Estimate local and tail-area FDRs, EPs and priorityFDRs

Local priorityFDR (denoted priorityfdr) estimates are obtained using the local analogue of Equation 1, and substituting in the estimates:

$$\widehat{\text{priorityfdr}}_i = \widehat{\text{fdr}}_i + \widehat{\text{ep}}_i \times (1 - \widehat{\text{fdr}}_i). \tag{51}$$

[17] In practice the four conditional ep estimates are estimated separately using new random subsets t_j each time, but the procedure is simpler to explain in terms of a single subset.

To obtain tail area FDR estimates for each variable i we use a standard approach of taking the mean local priorityFDR over variables with local priorityFDRs more extreme than variable i:

$$\widehat{\text{priorityFDR}}_i = \frac{\sum_{\widehat{\text{priorityfdr}}_j \leqslant \widehat{\text{priorityfdr}}_i} \widehat{\text{priorityfdr}}_j}{\sum_j I(\widehat{\text{priorityfdr}}_j \leqslant \widehat{\text{priorityfdr}}_i)}. \tag{52}$$

Although it would also be possible to plug in tail area estimates of FDR and EP as in Equation 1, which more intuitively represents the idea underlying tail-area priorityFDR estimation, the approach above may result in better performance when there are correlations between fdr and ep. Simulation studies demonstrating the effectiveness of these estimates are described in the main text.

3 Prior splitting theory

Splitting priors into non-negative and non-positive mixtures

During NPE/NPE-diff estimation, we model the ratios[18] $\Lambda_{(+)}(z)/\Lambda_{(u)}(z)$ and $\Lambda_{(-)}(z)/\Lambda_{(u)}(z)$ as curves similar to squared-polynomials, but for which the derivates are always non-negative and non-positive respectively (Section 2, Stage 2). In Stage 3 the same model is applied to scaled effect estimate differences, δ. Here we show why the resulting distribution models[19] $\Lambda_{(+)}(z)/W$ and $\Lambda_{(-)}(z)/W$ are conditional on $\beta > 0$ and $\beta < 0$ respectively, allowing the partitioning of the overall likelihood into mixtures with non-overlapping priors on β, which we term 'prior splitting'. Focussing first on the positive-effect distribution, based on the modelling assumptions provided in Section 2, $\Lambda_{(+)}(z)/W$ can be written as

$$\frac{\Lambda_{(+)}(z)}{W} = \int_{-\infty}^{\infty} f_x(z) g_{(+)}(x) dx, \tag{53}$$

where x is expected value of z, the observed z-score, also its scaled true effect size $\beta/\hat{s}$, $g_{(+)}(x)$ is its unknown prior distribution function, and $f_x(z)$ is a normal distribution with mean x and unit variance: $\frac{1}{\sqrt{2\pi}} e^{-(z-x)^2/2}$. As $\Lambda_{(u)}(z)/W = f_0(z)$, the ratio $\Lambda_{(+)}(z)/\Lambda_{(u)}(z)$ is

$$\frac{\Lambda_{(+)}(z)}{\Lambda_{(u)}(z)} \equiv R_{(+)}(z) = \int_{-\infty}^{\infty} e^{zx - x^2/2} g_{(+)}(x) dx, \tag{54}$$

[18]We illustrate the argument in this section with generic variables e.g. z, β and $\hat{s}$, dropping the subscripts.

[19]Recall that W is used to scale densities to give probability masses falling into z-bins of width W (see Equation 22).

which has the derivative

$$R'_{(+)}(z) = \int_{-\infty}^{\infty} x e^{zx - x^2/2} g_{(+)}(x) dx. \tag{55}$$

It can now be seen that whenever $g_{(+)}(x) > 0$ for any $x < 0$, one can find a $z < 0$ that is sufficiently negatively large that for any $y > 0$,

$$e^{zx - x^2/2} g_{(+)}(x) \gg e^{zy - y^2/2} g_{(+)}(y) \geqslant 0, \tag{56}$$

so that

$$x e^{zx - x^2/2} g_{(+)}(x) + y e^{zy - y^2/2} g_{(+)}(y) \ll 0 \tag{57}$$

As such $R'_{(+)}(z)$ being negative anywhere is a necessary condition for $g_{(+)}(x) > 0$ when any $x < 0$. It follows that non-negativity of $R'_{(+)}(z)$ is a sufficient condition for $g(x) = 0$ when any $x < 0$, and as we have constrained $R'_{(+)}(z)$ to be non-negative everywhere this implies that $g_{(+)}(x) = 0$ for any $x < 0$.

The reverse implication, that $g_{(+)}(x) = 0$ for all $x < 0$ implies $R'_{(+)}(z) \geqslant 0$, is apparent in Equation 55, as the right hand side can only be negative when there is at least one $x < 0$ for which $g_{(+)}(x) > 0$. Therefore, $R'_{(+)}(z) \geqslant 0$ if and only if $g_{(+)}(x) = 0$ for all $x < 0$.

The same essential reasoning (with signs reversed) can be applied to $R_{(-)}(z)$. As its derivative is non-positive everywhere, neither a positive z or positive x can be found for which

$$x e^{zx - x^2/2} g_{(-)}(x) + y e^{zy - y^2/2} g_{(-)}(y) \gg 0, \tag{58}$$

for any y where $y < 0$, therefore $g_{(-)}(x) = 0$ for any $x > 0$. Conversely, replacing $g_{(+)}(x)$ with $g_{(-)}(x)$ in Equation 55, one sees that the right hand side can only be positive when there is at least one $x > 0$ for which $g_{(-)}(x) > 0$. Therefore, $g_{(-)}(x) = 0$ for all $x > 0$ implies that $R'_{(-)}(z) \leqslant 0$, so $R'_{(-)}(z) \leqslant 0$ if and only if $g_{(-)}(x) = 0$ for all $x > 0$.

The theory we have presented is analogous to Tweedie's formula [Efron and Hastie, 2016, Robbins, 1956], which uses the derivative of a log-likelihood function to estimate the posterior expectation of x conditioned on z. Our innovation is to constrain the derivative of the

likelihood:null-likelihood ratio, allowing estimation of likelihoods with non-overlapping prior distributions of effects across either side of a given threshold, in this case zero.

Splitting from the zero-effect distribution

While the reasoning above demonstrates that the prior distributions $g_{(+)}(x)$ and $g_{(-)}(x)$ both equal zero for negative and positive x respectively, they should also ideally be constrained to equal zero when $x = 0$. Assuming that $g_{(+)}(x) = 0$ for $x < 0$ as shown in the previous subsection, $R_{(+)}(z)$ decreases as $z \to (-\infty)$, due to $(zx - x^2/2)$ decreasing inside the exponent whenever x is positive (see Equation 54). If it is also the case that $g_{(+)}(0) = 0$, $R_{(+)}(z) \to 0$ as $z \to (-\infty)$, because the exponent approaches zero for any $g_{(+)}(x) > 0$. This is not the case if $g_{(+)}(0) > 0$, as there will be a 'sill' at $g_{(+)}(0)$ in the function $R_{(+)}(z)$ as $z \to (-\infty)$, due to $e^{z0-0^2/2} = 1$. Therefore, $R_{(+)}(z) \to 0$ as $z \to (-\infty)$ if and only if $g_{(+)}(0) = 0$.

The condition is met as the expression inside the exponent on the right hand side of Equation 22 becomes dominated by the largest power of z (when $n = D$ and $p = D$), so as $z \to (-\infty)$ the ratio approaches[20]

$$R_{(+)}(z) \to e^{z \frac{\Gamma_D \Gamma_D z^D z^D}{2D+1}} \to 0. \tag{59}$$

This ensures that the prior $g_{(+)}(x)$ has zero probability at $x = 0$. Together with the reasoning in the previous subsection we are thus able to state that $g_{(+)}(x) = 0$ for all $x \leqslant 0$.

Reversing the logic shows that the right hand side of Equation 23 approaches zero as $z \to \infty$. Because $R_{(-)}(z) \to 0$ as $z \to \infty$ if and only if $g_{(-)}(0) = 0$, this ensures that $g_{(-)}(x) = 0$ for all $x \geqslant 0$.

[20] This assumes that $|\Gamma_D| > 0$ which should be the case, but if not the same reasoning can be applied to $D - 1$, or to whichever is the largest term in the polynomial which for which $|\Gamma_n| > 0$.

References

[Broyden, 1970] Broyden, C. G. (1970). The Convergence of a Class of Double-rank Minimization Algorithms 1. General Considerations. *IMA Journal of Applied Mathematics*, 6(1):76–90.

[Dempster et al., 1977] Dempster, A. P., Laird, N. M., and Rubin, D. B. (1977). Maximum likelihood from incomplete data via the em algorithm. *Journal of the Royal Statistical Society: Series B (Methodological)*, 39(1):1–22.

[Efron and Hastie, 2016] Efron, B. and Hastie, T. (2016). *Computer Age Statistical Inference: Algorithms, Evidence, and Data Science*. Institute of Mathematical Statistics Monographs. Cambridge University Press.

[Fletcher, 1970] Fletcher, R. (1970). A new approach to variable metric algorithms. *The Computer Journal*, 13(3):317–322.

[Goldfarb, 1970] Goldfarb, D. (1970). A family of variable-metric methods derived by variational means. *Mathematics of Computation*, 24(109):23–26.

[Hastie et al., 2009] Hastie, T., Tibshirani, R., Friedman, J. H., and Friedman, J. H. (2009). *The elements of statistical learning: data mining, inference, and prediction*, volume 2. Springer.

[Lindsey, 1974a] Lindsey, J. K. (1974a). Comparison of probability distributions. *Journal of the Royal Statistical Society: Series B (Methodological)*, 36(1):38–47.

[Lindsey, 1974b] Lindsey, J. K. (1974b). Construction and comparison of statistical models. *Journal of the Royal Statistical Society. Series B (Methodological)*, 36(3):418–425.

[Robbins, 1956] Robbins, H. (1956). An empirical Bayes approach to statistics. Proc. 3rd Berkeley Sympos. Math. Statist. Probability 1, 157-163 (1956).

[Shanno, 1970] Shanno, D. F. (1970). Conditioning of quasi-newton methods for function minimization. *Mathematics of Computation*, 24(111):647–656.
