# Supplementary material for: Bayesian Effect Size Ranking to Prioritise Genetic Risk Variants in Common Diseases for Follow‐Up Studies
Source: Genet Epidemiol. 2025 Jan 3;49(1):e22608. doi: 10.1002/gepi.22608 (PMC11696485; doi:10.1002/gepi.22608)
Supplement: Supplementary file 2 — Supporting information. [file GEPI-49-0-s001.pdf]

Figure S1

Z-scores and true effect sizes on the Z-score scale (divided by standard error for each variable) for a single simulated dataset (N=10000, truncated at ±6) chosen from among the 100 simulations used for priorityFDR testing, a) at original power and b) with increased power due to a decreased proportion of null variables and higher variance in true non-null effect sizes (see methods). The theoretical null distribution of Z-scores (when all true effects are zero) is shown in black.

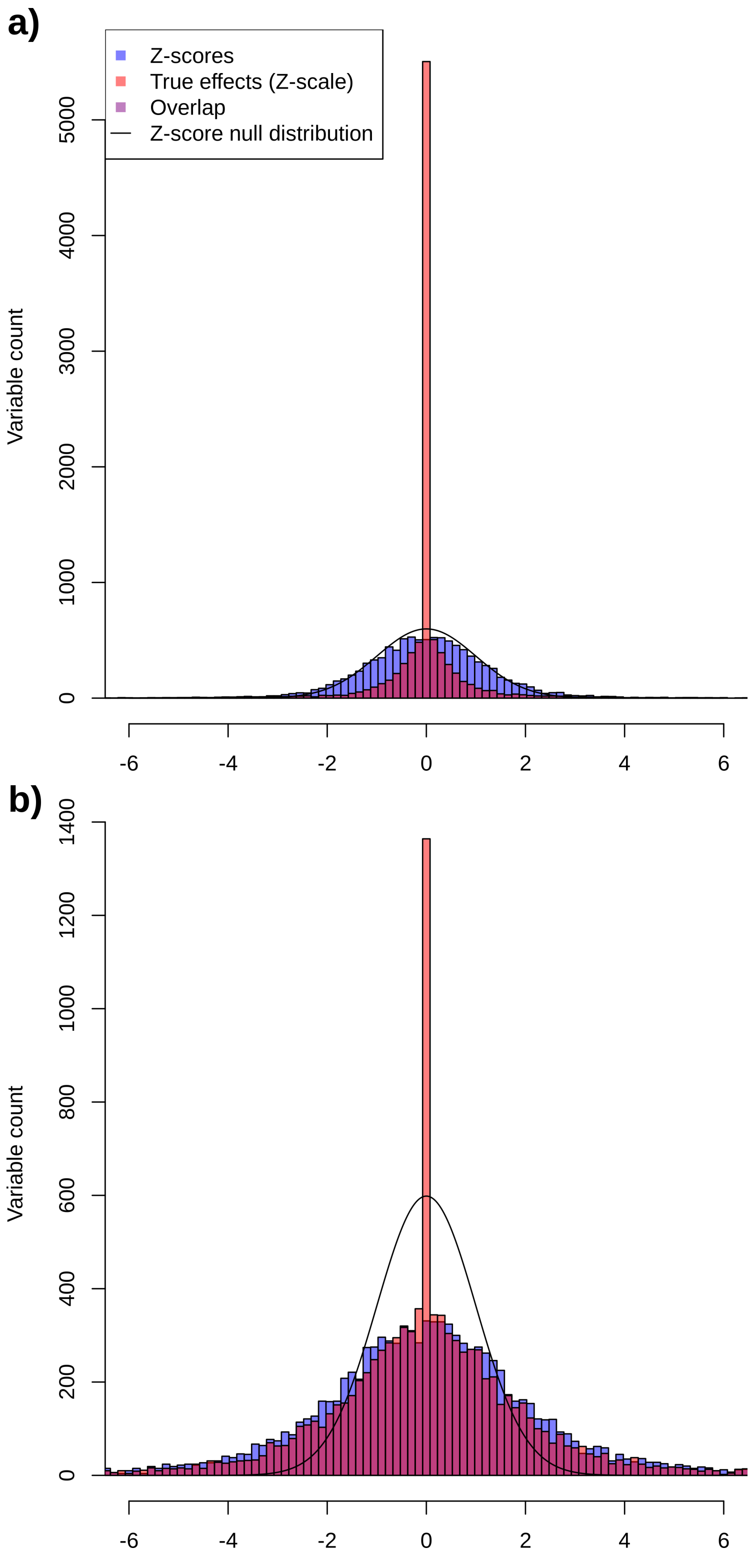

Figure S2

Estimated priorityFDRs versus true effect size rankings for a high-powered version of the simulated dataset used in main text Figure 2. Non-null variables ($n \approx 9K$) are shown in (a) plotted against their true effect size ranks against all other non-null variables, and the distribution of priorityFDRs among null variables ($n_{null} \approx 1K$), which have true effects of zero, are shown in (b). Plotted priorityFDRs are local rather than tail-area estimates.

a)

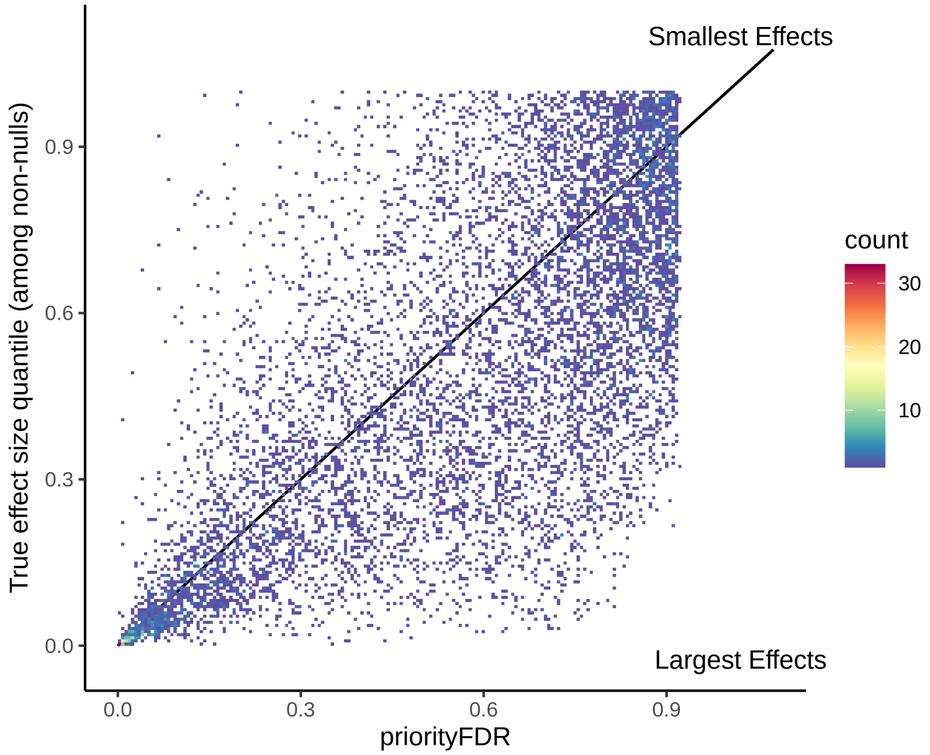

b)

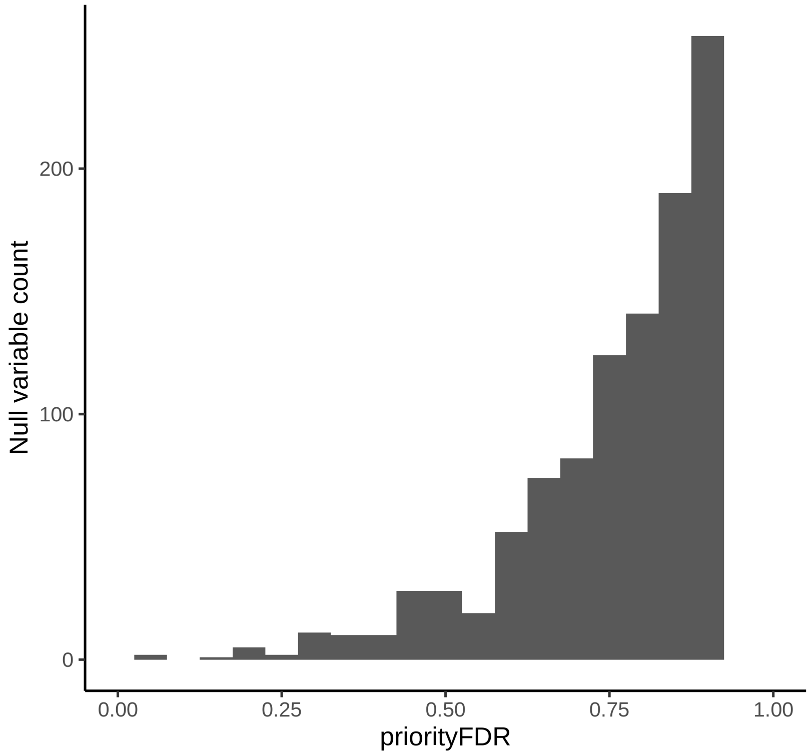

Figure S3

Estimated inclusive priorityFDRs ($\text{priorityFDR}^{(\text{inc})}$) versus true effect size rankings for the simulated dataset used in main text Figure 2 (n=10K). Non-null variables (n≈5K) are shown in (a) plotted against their true effect size ranks against all other variables, and the distribution of $\text{priorityFDR}^{(\text{inc})}$s among null variables ($n_{null}$≈5K), which have true effects of zero, are shown in (b). Plotted priorityFDRs are local rather than tail-area estimates.

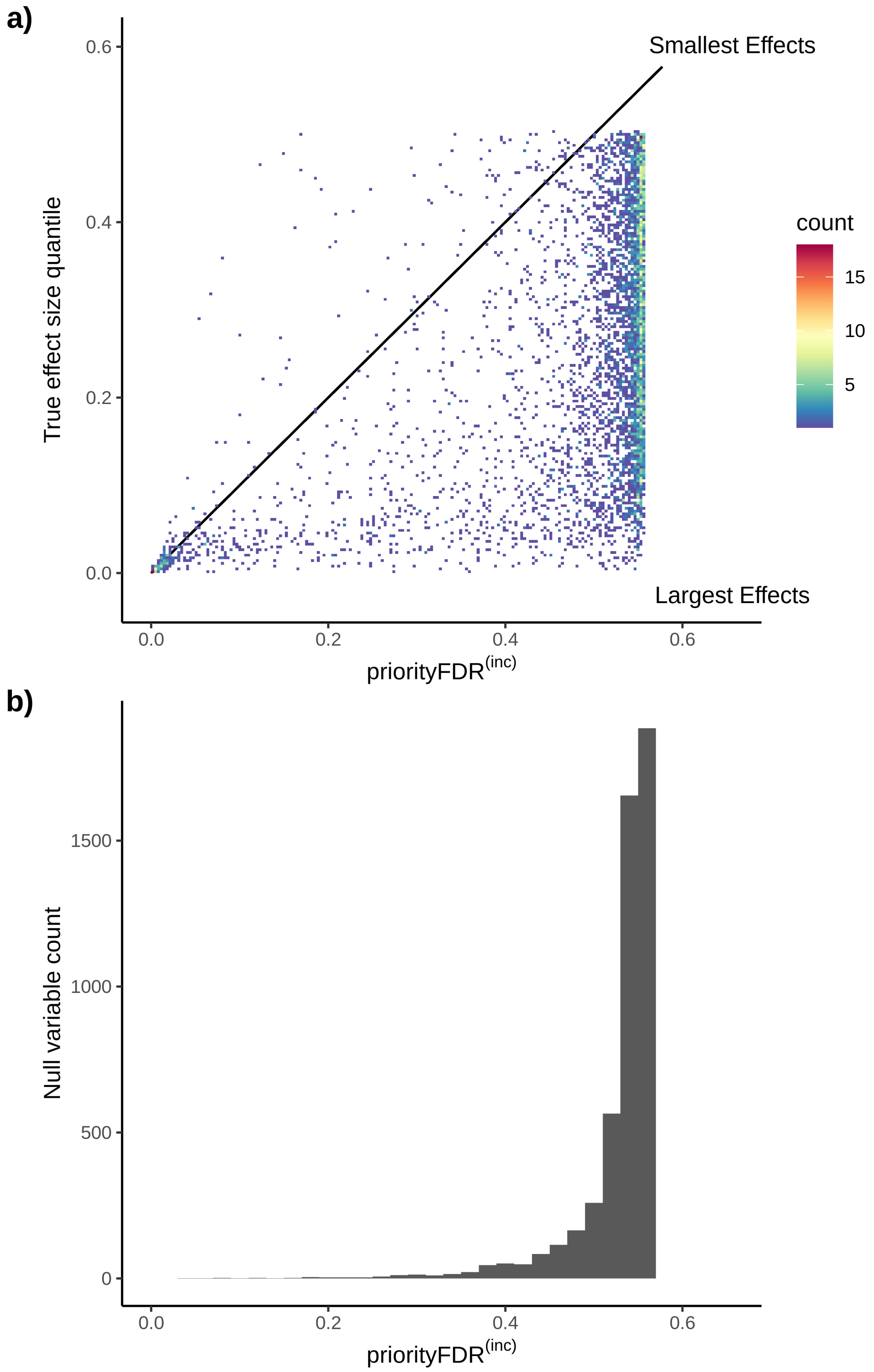

Figure S4

Estimated inclusive priorityFDRs (priorityFDR$^{(inc)}$) versus true effect size rankings for the high-powered simulated dataset used in Figure S2 (n=10K). Non-null variables (n≈9K) are shown in (a) plotted against their true effect size ranks against all other variables, and the distribution of priorityFDR$^{(inc)}$s among null variables (n_{null}≈1K), which have true effects of zero, are shown in (b). Plotted priorityFDRs are local rather than tail-area estimates.

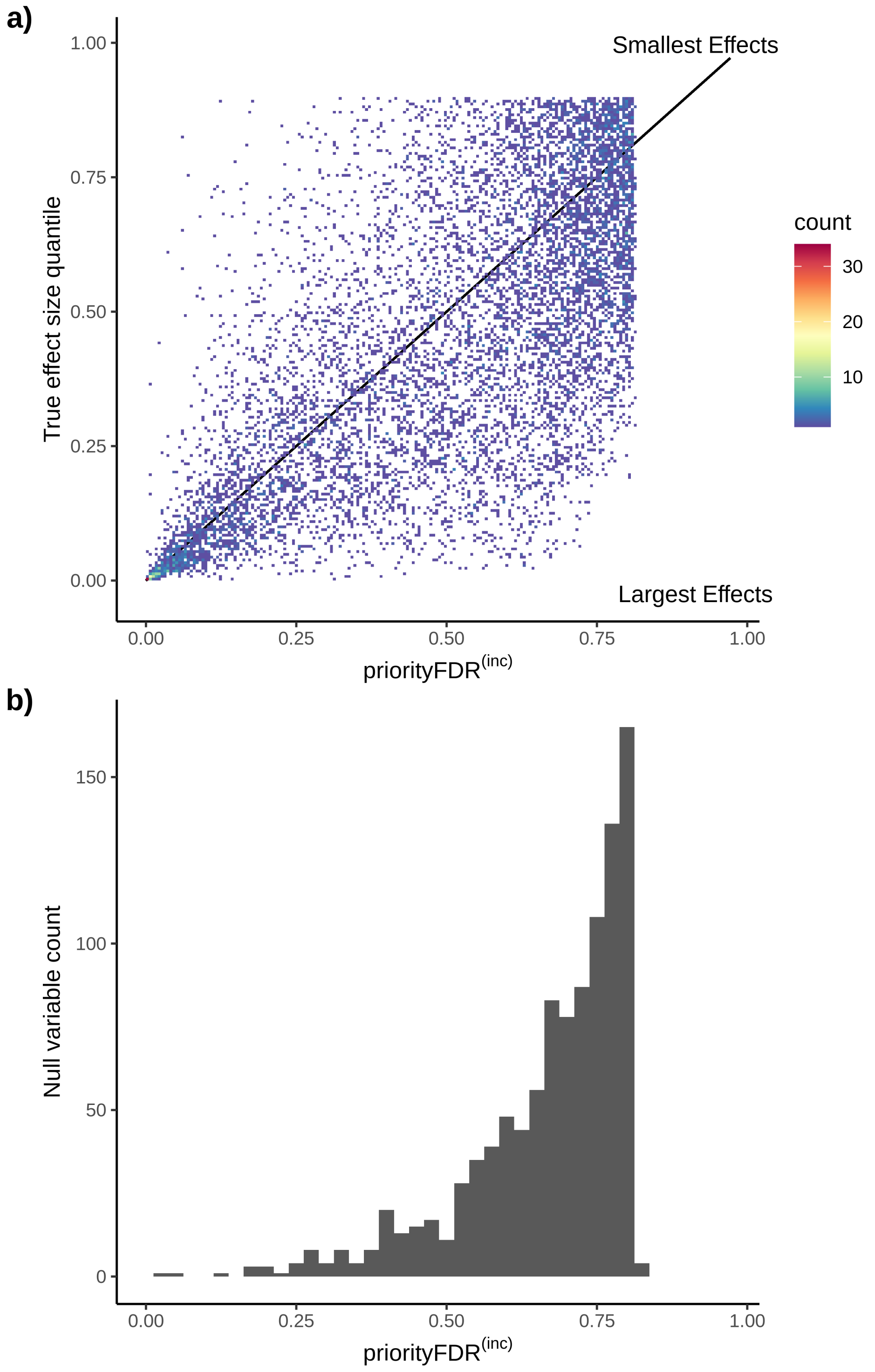

Figure S5

Results from Figures 2, S2, S3 and S4 restricting to variables satisfying 1%, 0.5% and 0.1% FDR thresholds. Consistent with other analyses, FDRs were computed using our estimated priorityFDR models, rather than using the Benjamini-Hochberg method. Simulations (both regular and high power) were the same as in the other analyses, and priorityFDR modelling was performed using all simulated variables, according to its intended usage. Relationships between estimated priorityFDRs and true effect size quantiles were similar across the different levels of statistical significance. Where panel (b) is empty this is because no null variables were found passing the FDR threshold.

i) Variables with FDR≤1%, regular power

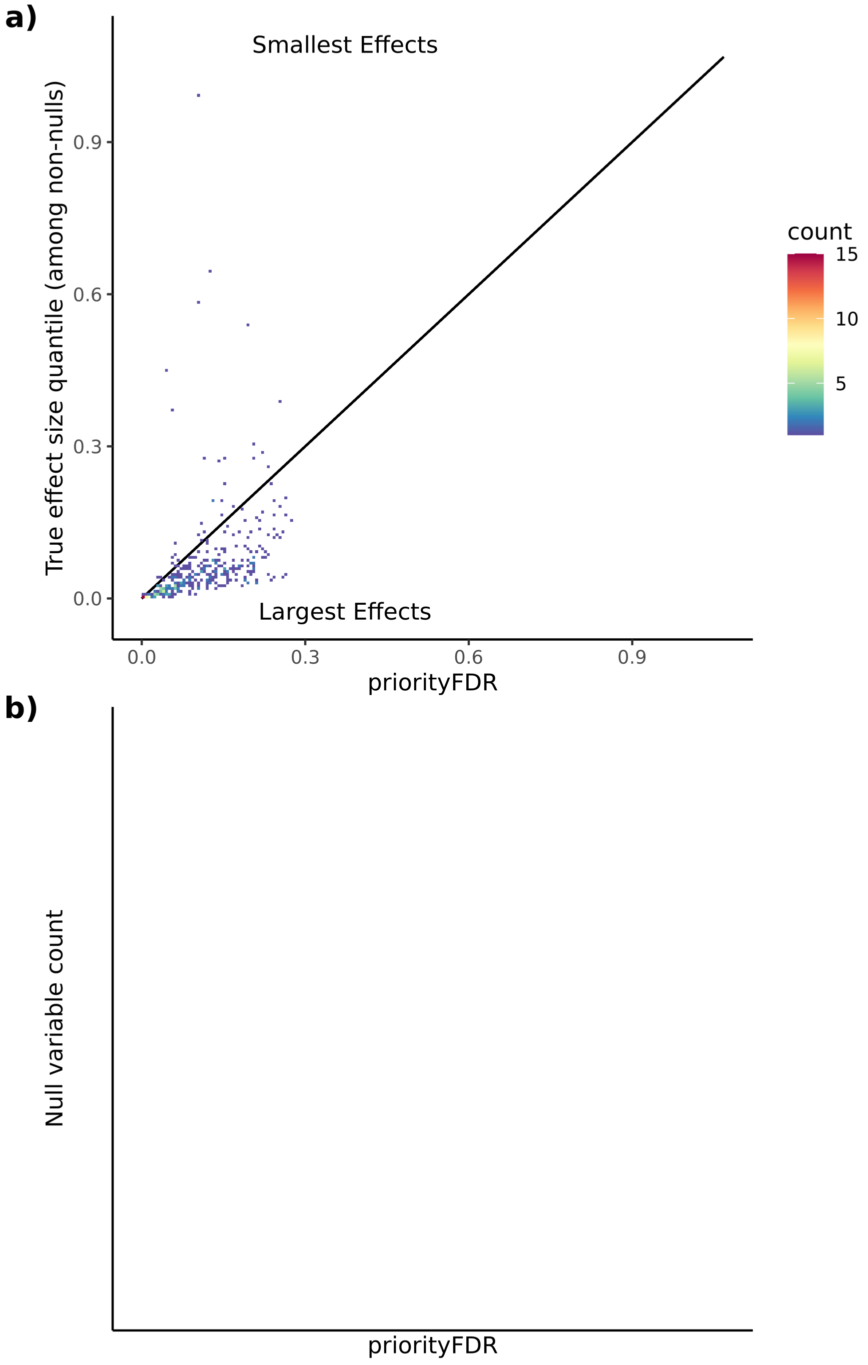

ii) Variables with FDR≤1%, high power

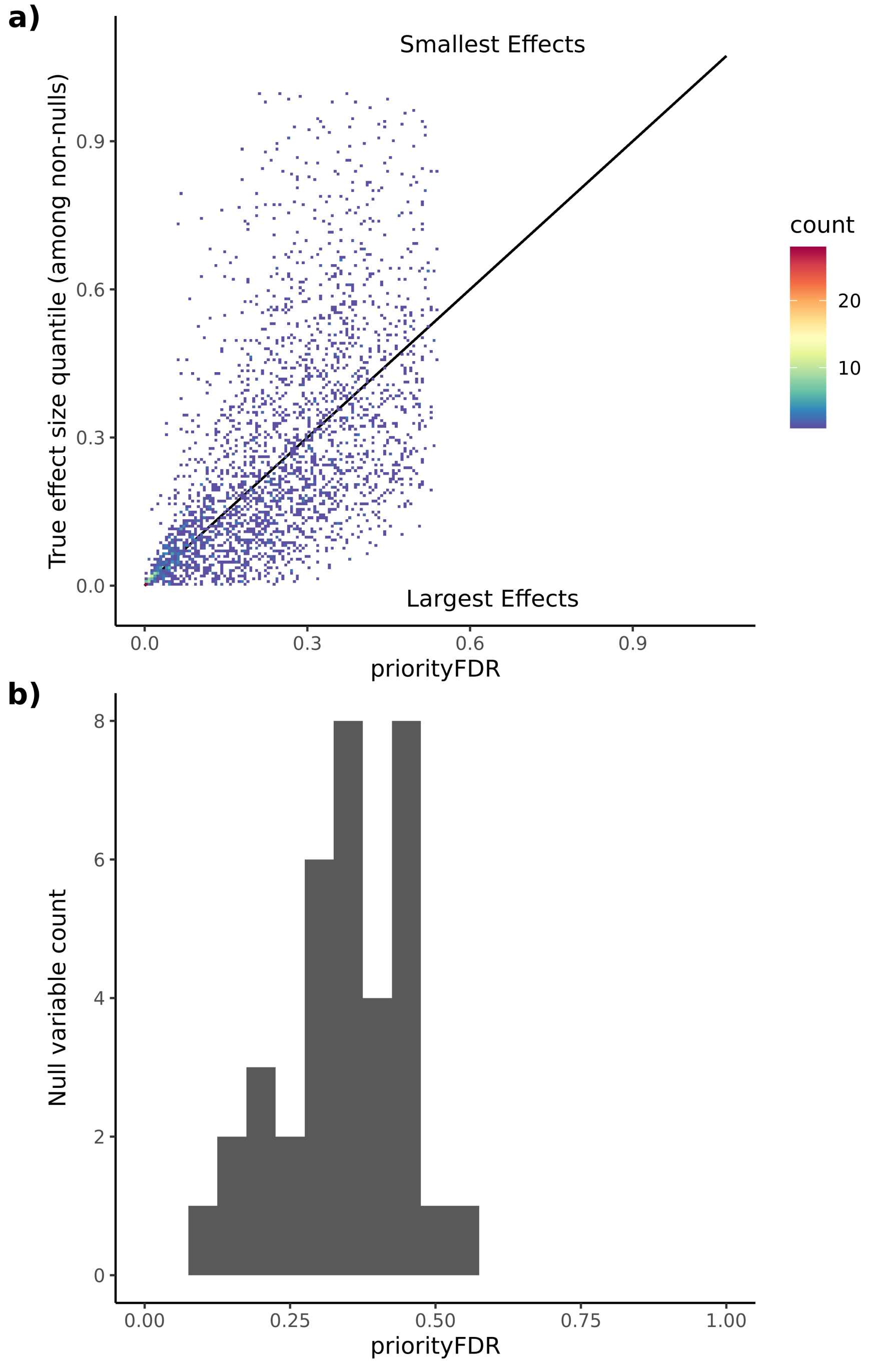

iii) Variables with FDR≤0.5%, regular power

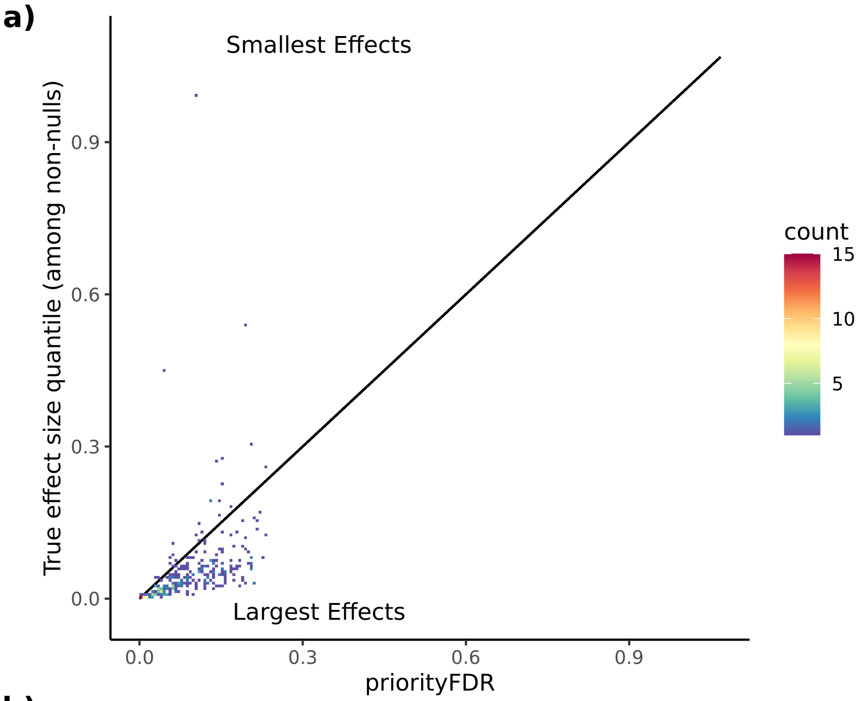

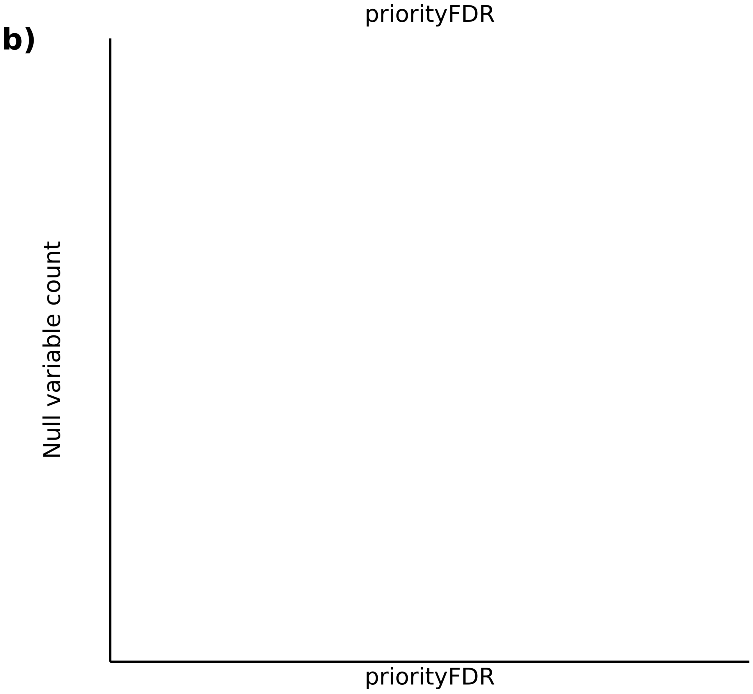

iv) Variables with FDR≤0.5%, high power

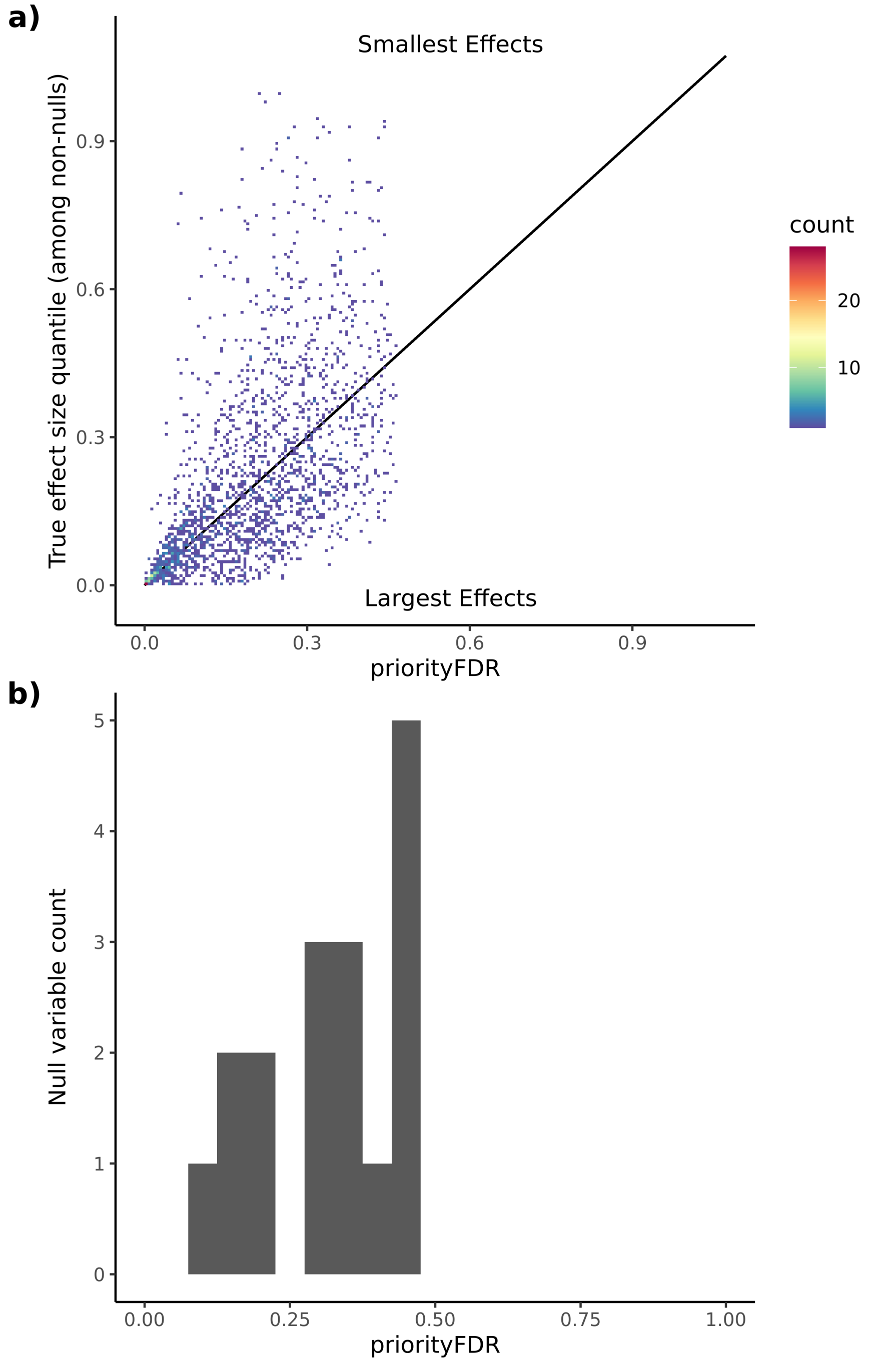

v) Variables with FDR≤0.1%, regular power

a)

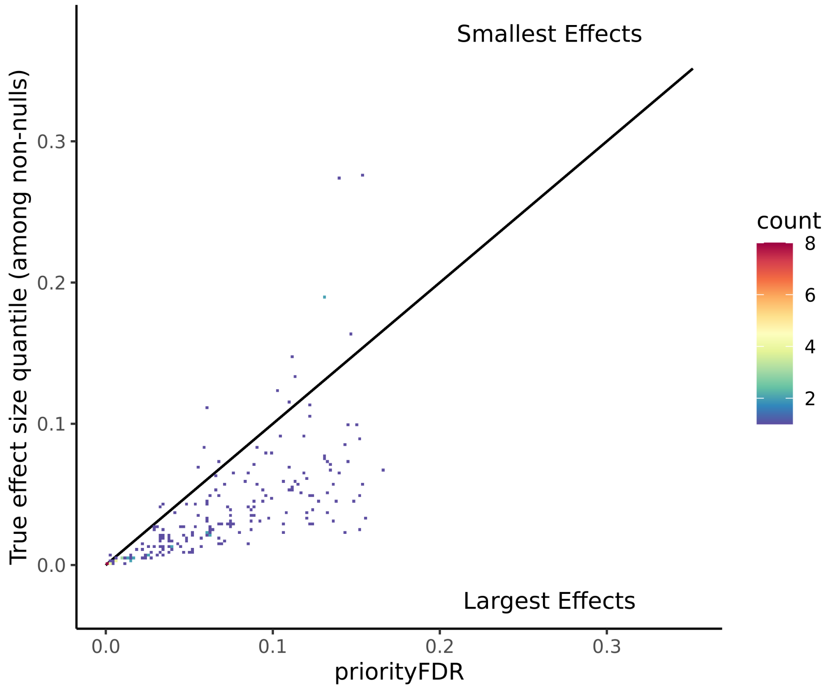

b)

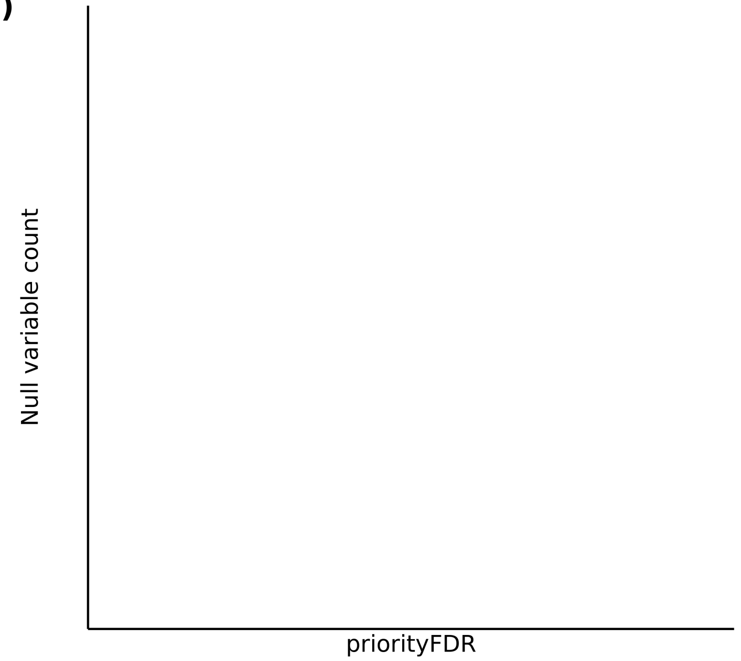

vi) Variables with FDR≤0.1%, high power

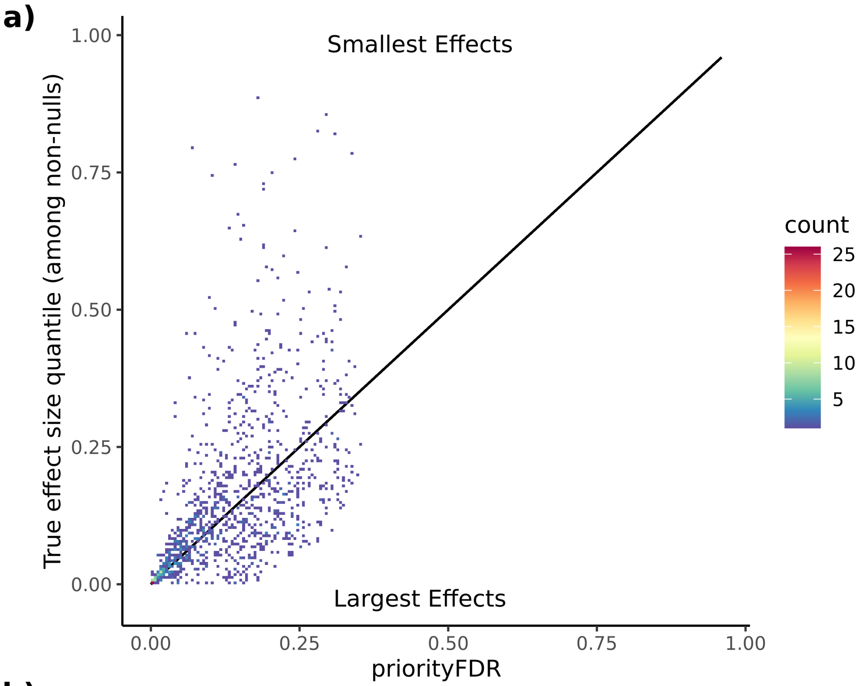

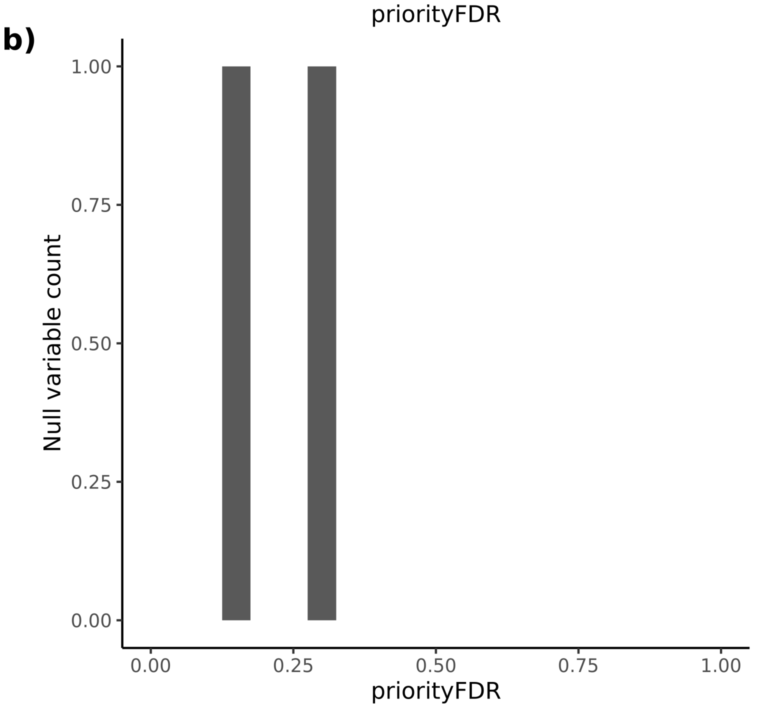

<u>Figure S6</u>

Simulation study from Figure 1 repeated, but restricting to variables satisfying 1%, 0.5% and 0.1% FDR thresholds. Consistent with other analyses, FDRs were computed using our estimated priorityFDR models, rather than using the Benjamini-Hochberg method. Average error rates of a) priorityFDR and b) FDR estimates for 100 GWAS simulations at 14 α thresholds (10^{-3}, 10^{-2}, 2.5×10^{-2}, 5×10^{-2}, and 0.1 to 1 in increments of 0.1) in grey, with means and 95% confidence intervals of the grey points shown in black. Each simulated GWAS contained 10,000 variants. Error rates were controlled correctly on average, but conservatively so at more lenient priorityFDR thresholds (x-axis), as low FDR variables are also likely to have low priorityFDRs.

<u>i) Variables with FDR≤1%</u>

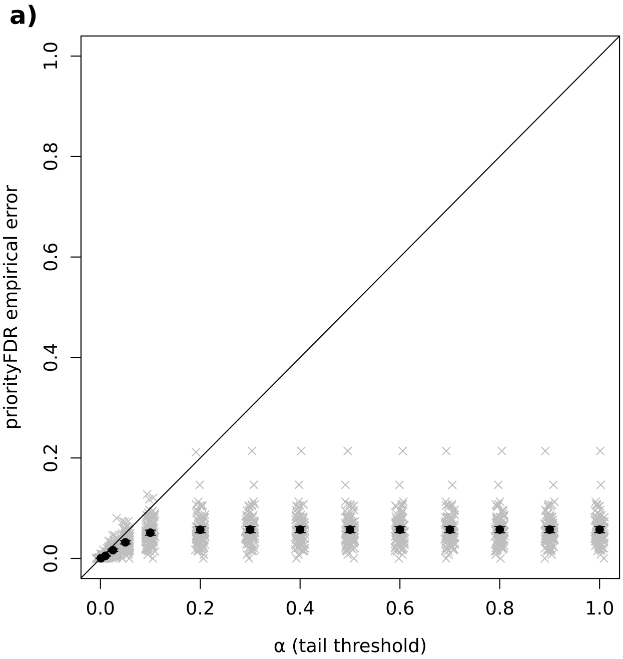

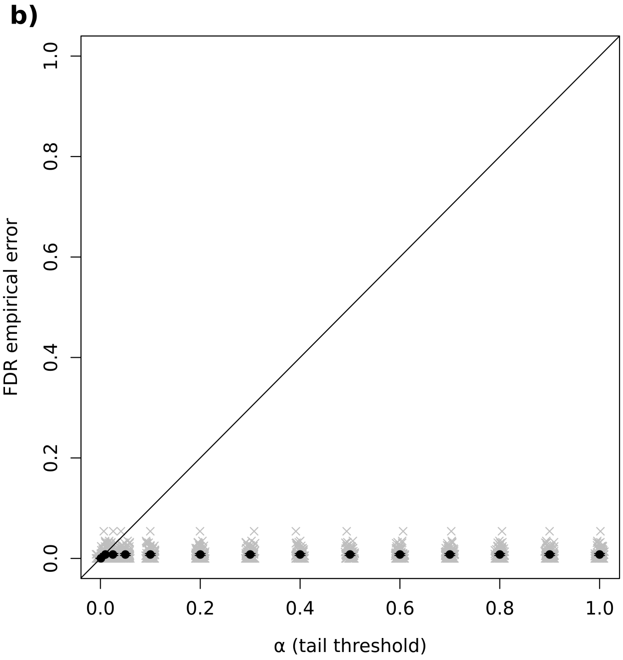

ii) Variables with FDR≤0.5%

a)

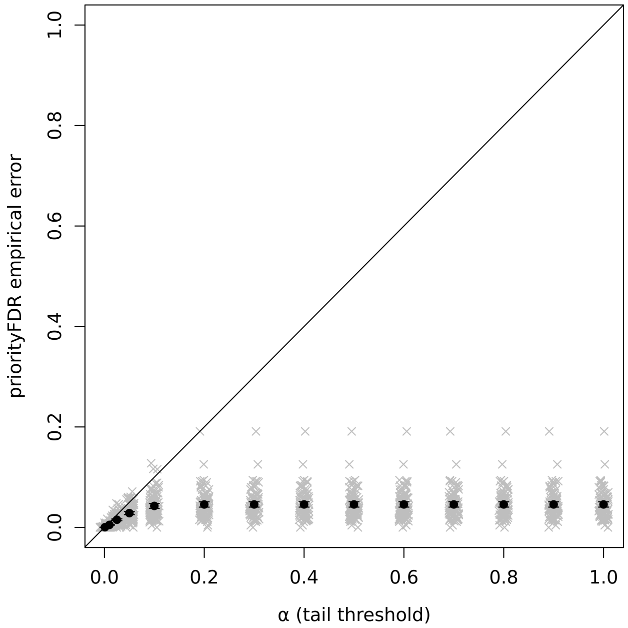

b)

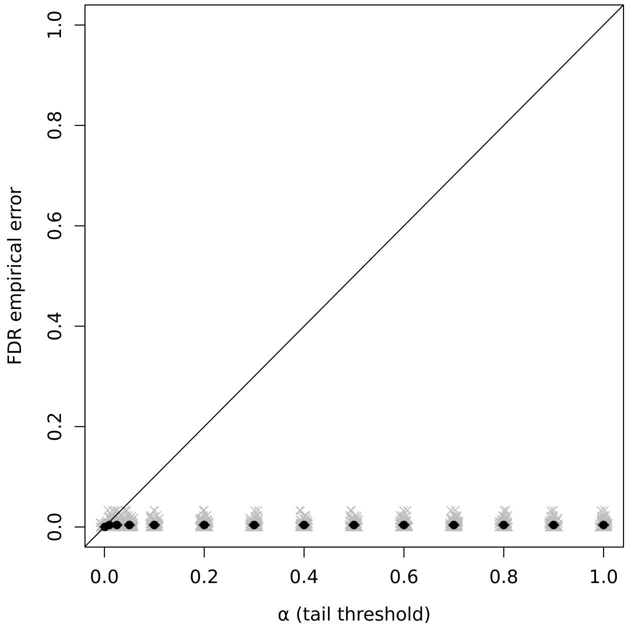

iii) Variables with FDR≤0.1%

a)

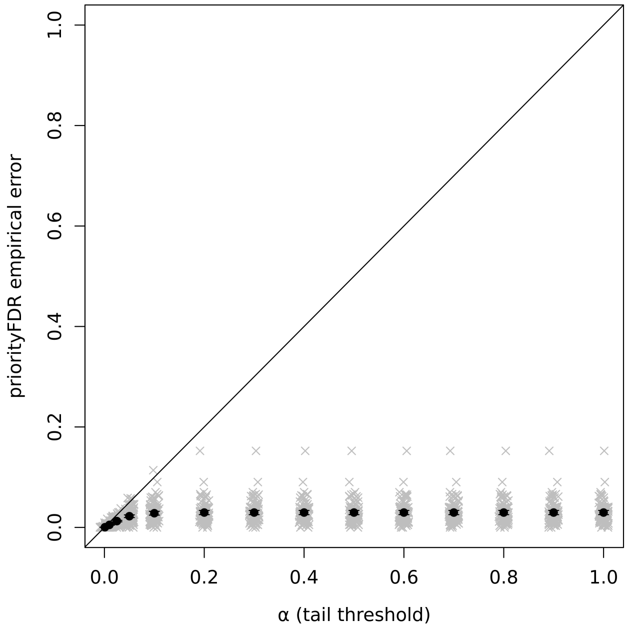

b)

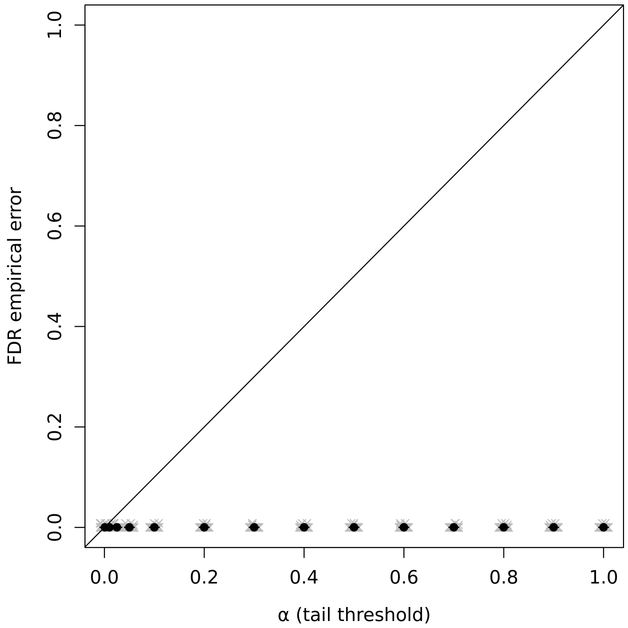

Figure S7

Simulation study from Figure 1 repeated with simulated correlations between the effects of variables, as occurs in the form of LD between genetic variants. Average error rates of a) priorityFDR and b) FDR estimates for 100 GWAS simulations at 14 α thresholds (10^{-3}, 10^{-2}, 2.5×10^{-2}, 5×10^{-2}, and 0.1 to 1 in increments of 0.1) in grey, with means and 95% confidence intervals of the grey points shown in black. Each simulated GWAS contained 10,000 variants. Results are similar to those without any simulation of correlations (Figure 1).

a)

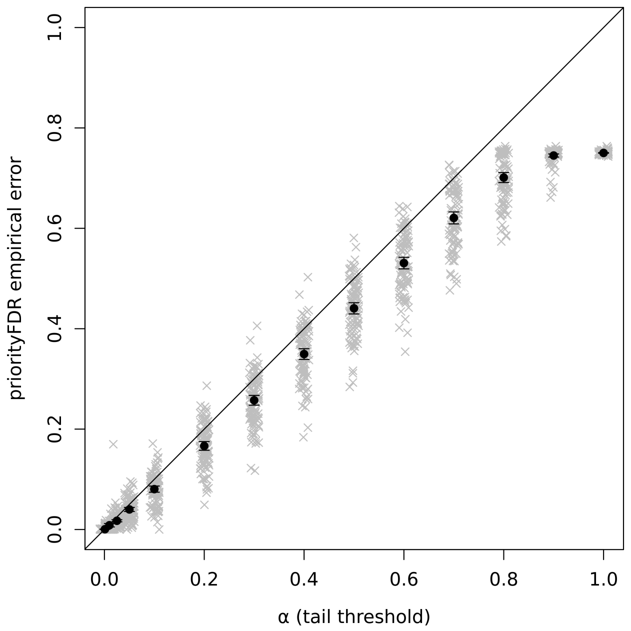

b)

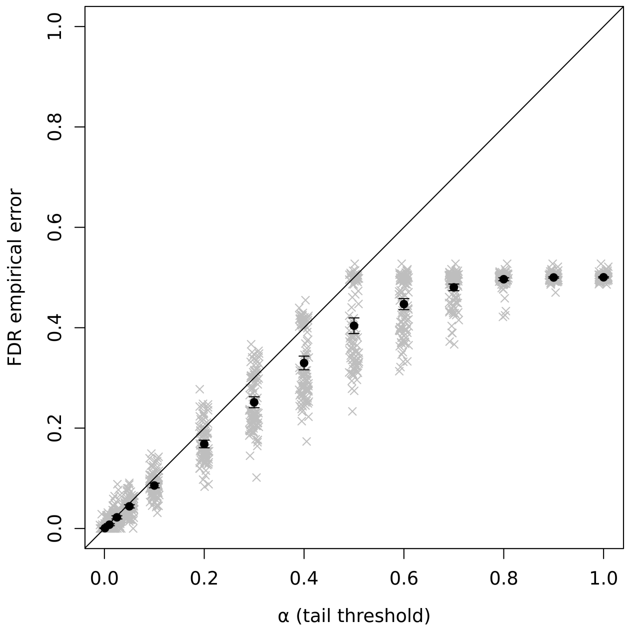

Figure S8

Manhattan plot of T1D GWAS associations. Associations lying above the blue line have FDR below≤1% FDR threshold, and the red line corresponds to the genome-wide significance threshold of 5×10^{-8}. Lead variants for each independent region are highlighted in green. Figure produced using the R package qqman (Turner, 2018).

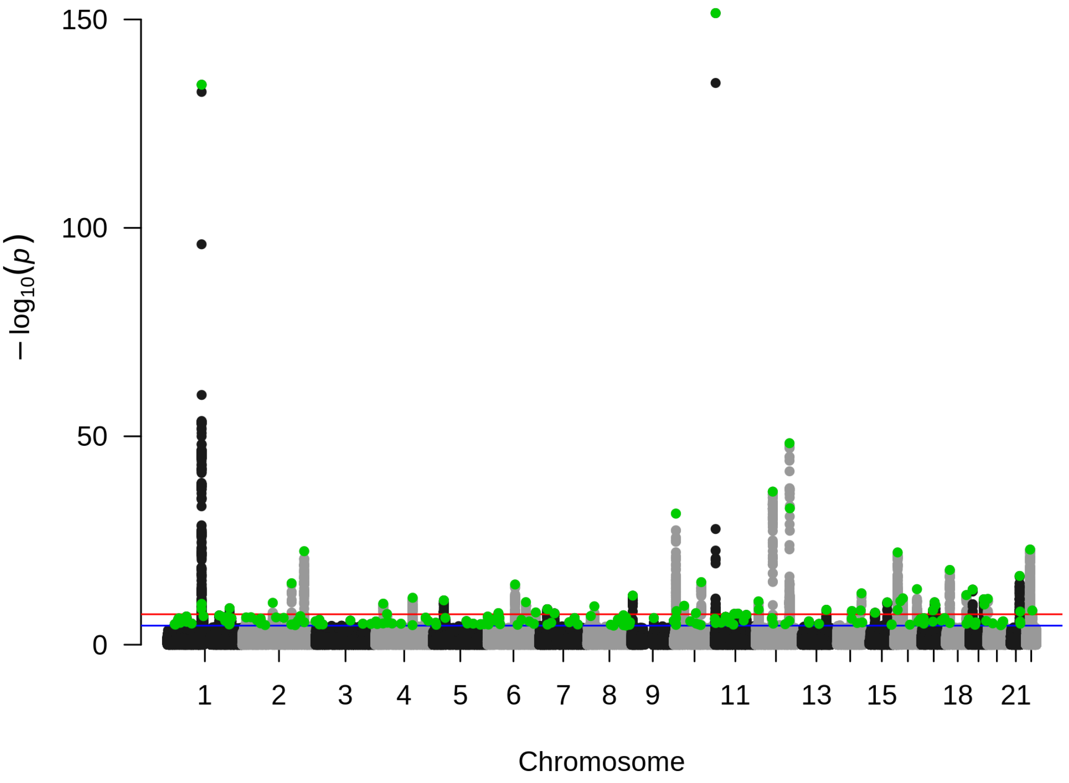

Figure S9

Quantile-quantile plot of observed versus expected T1D GWAS P values under the null hypothesis, excluding HLA region, with genomic control inflation factor of 1.12 (median Chi-square divided by 0.456, the median under the null hypothesis).

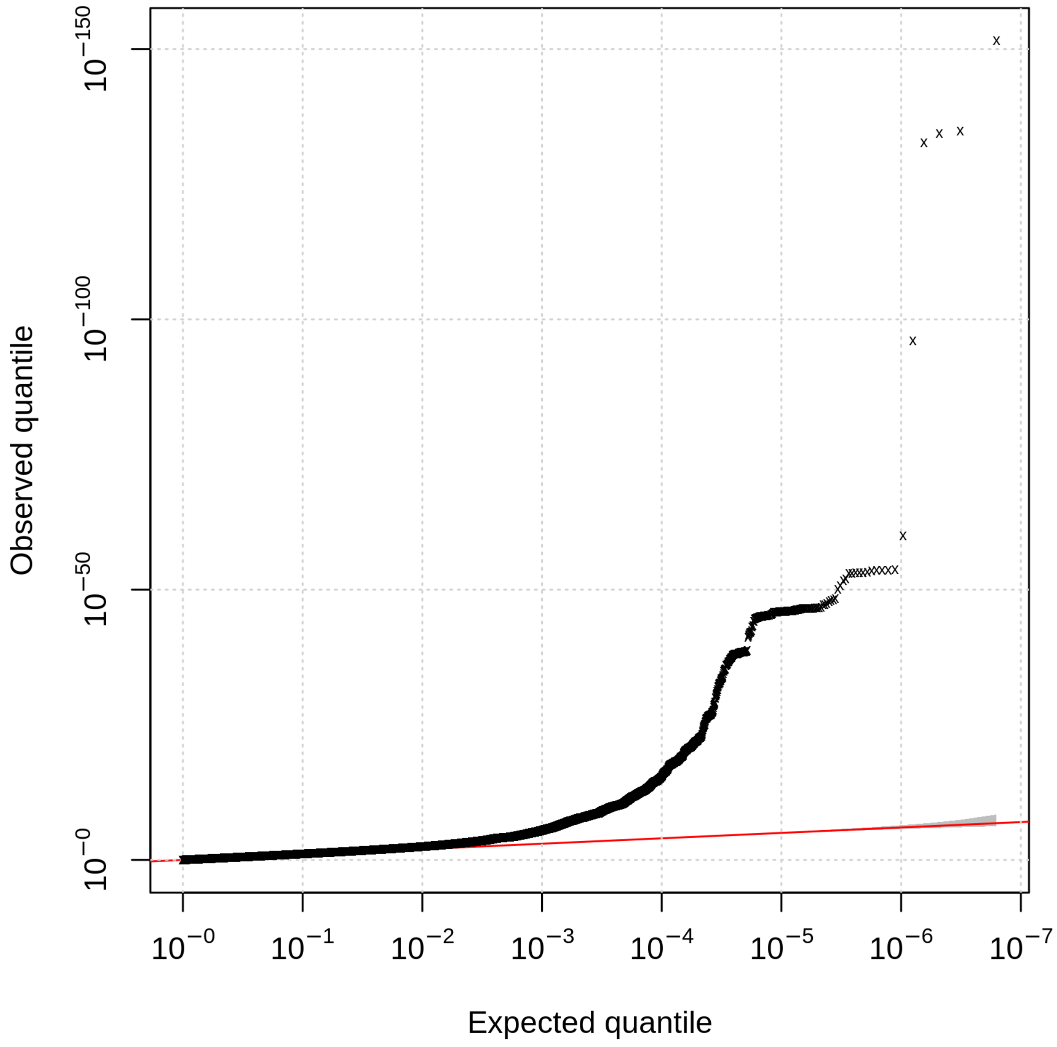

Figure S10

Fine-mapping results for previously unreported signals from Table 2 (genome-wide signals plus those with priorityFDRs within the same range), performed using the UK Illumina cohort only. Data for *RLIMP2* and *MAGI3* are not shown as the nearby presence of PTPN22 makes fine-mapping challenging. When present in a credible set, the location of the lead GWAS variant is indicated by an asterisk (coloured by credible set). Full T1D fine-mapping data is provided in Table S5.

a) *SLC25A37*

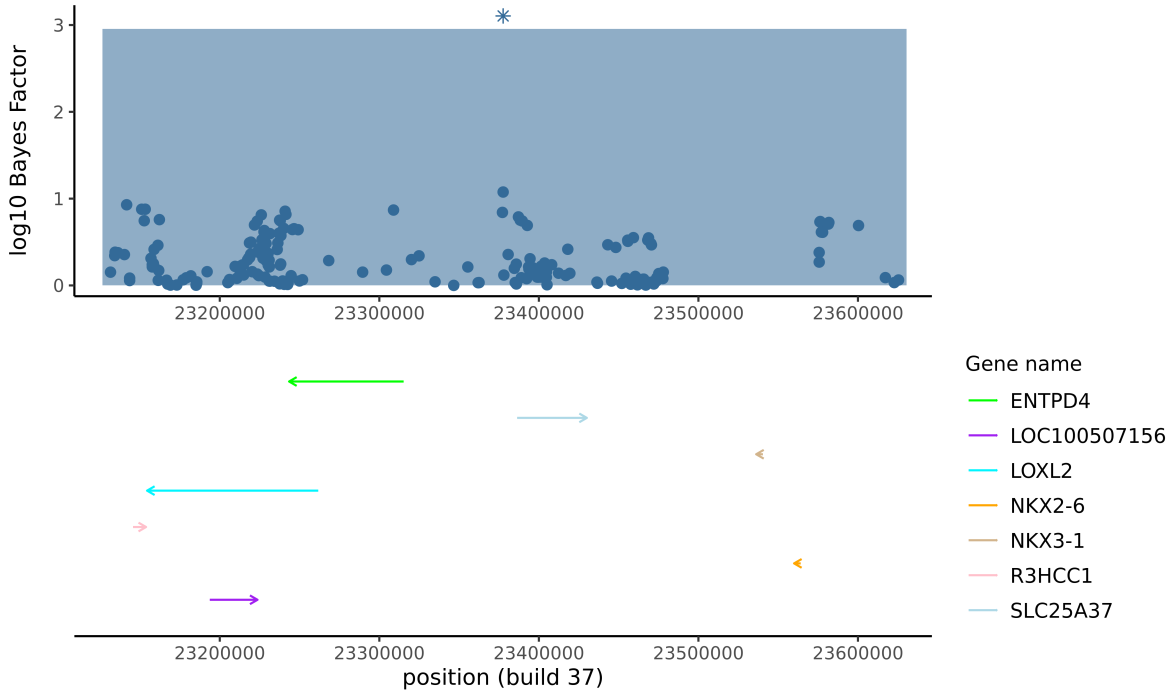

b) *LHFPL5*

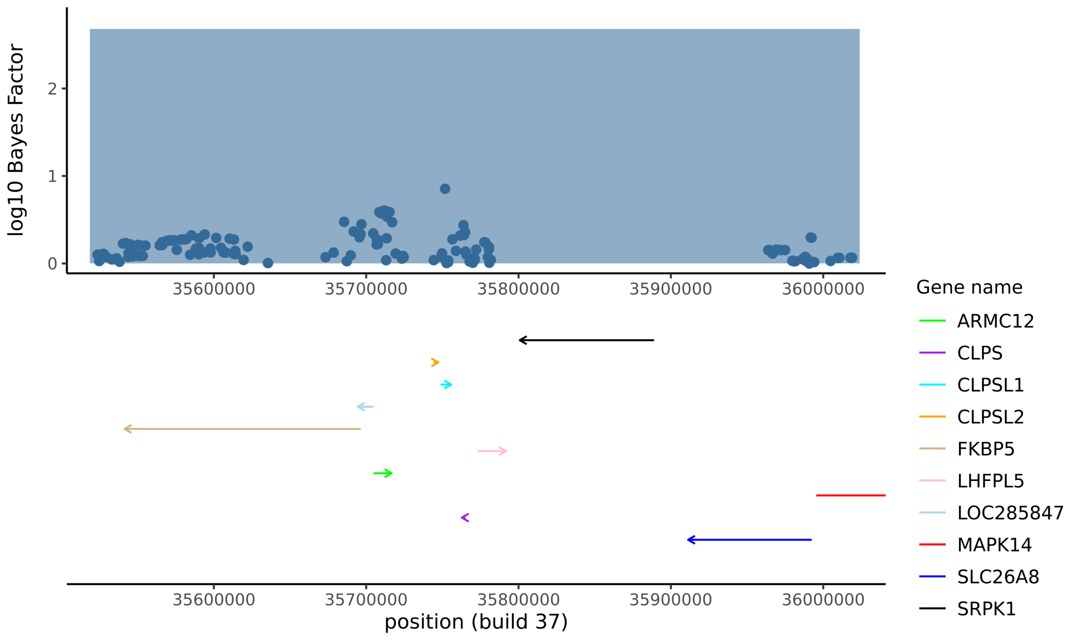

c) *ID4*

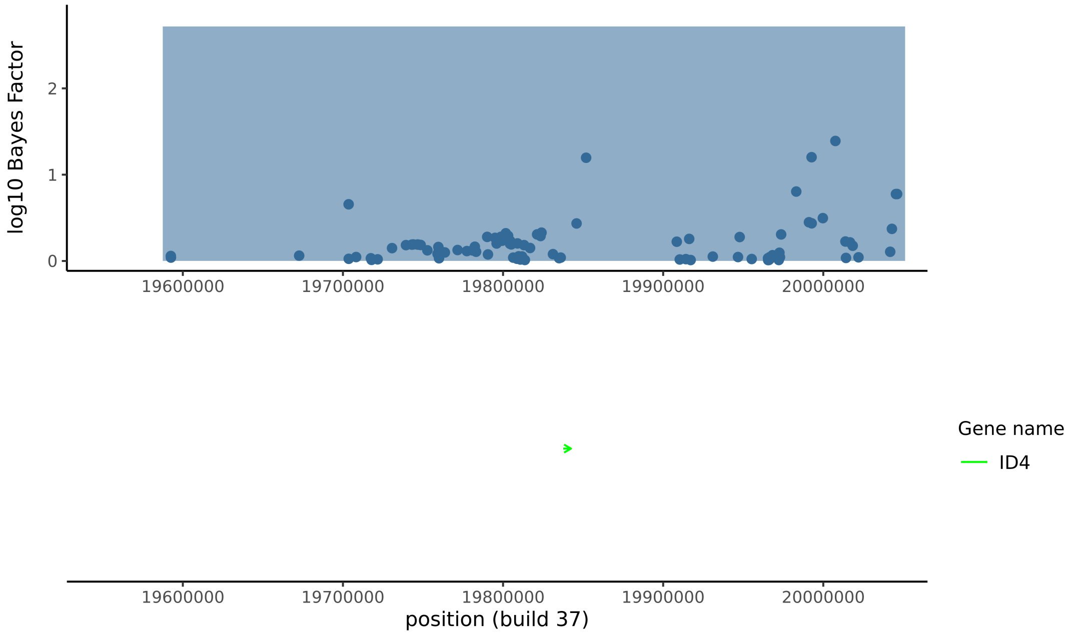

d) *ZBTB20*

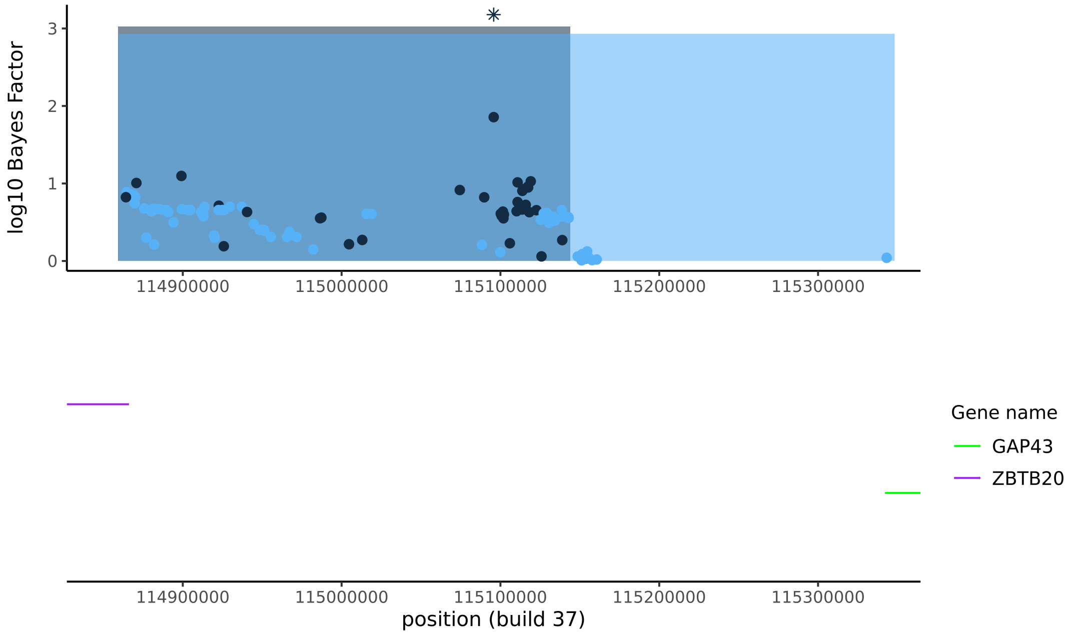

Figure S11

Alternative version of main text Figure 4 with a different inflection point at priorityFDR=0.5%. The OR_{risk} threshold is set at 1.09 as this is the smallest OR_{risk} among lead variants with priorityFDR≤0.5%.

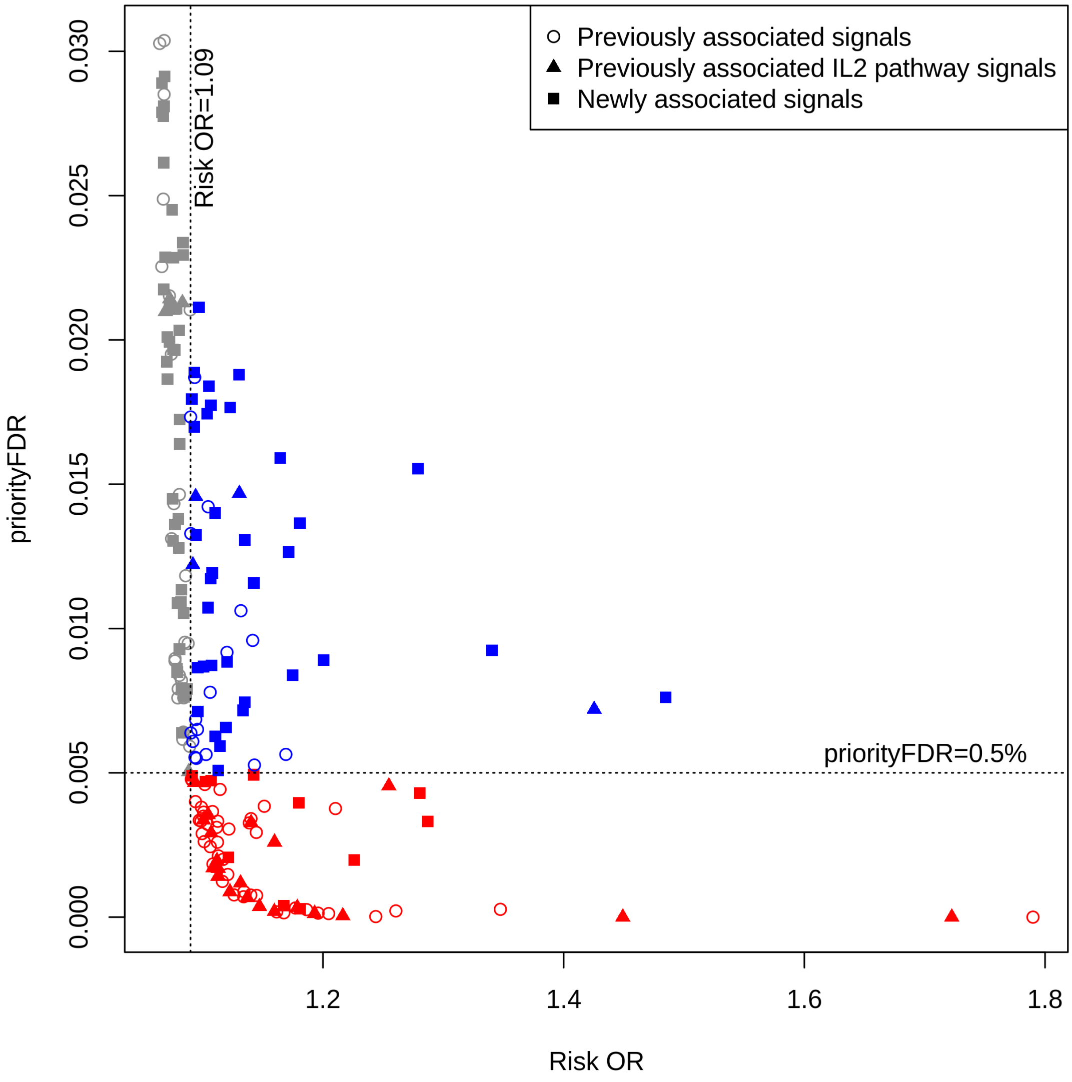

Figure S12

Meta-analysis forest plots for lead GWAS variant effects in the three priorityFDR groups: a) red group (smallest priorityFDRs), b) blue group (large effect size estimates but priorityFDRs greater than the red group) and c) grey group (neither low priorityFDRs or large effect size estimates). The three group names refer to their colours in main text Figure 4. The 26 signals with lowest priorityFDRs are shown within each group (all signals shown for the red group, with n=26). Error bars represent 95% confidence intervals. Variants are ordered from most positive to most negative inferred effects, as determined using priorityFDRs and the signs of observed effect estimates. Data for all signals are shown in Table S2.

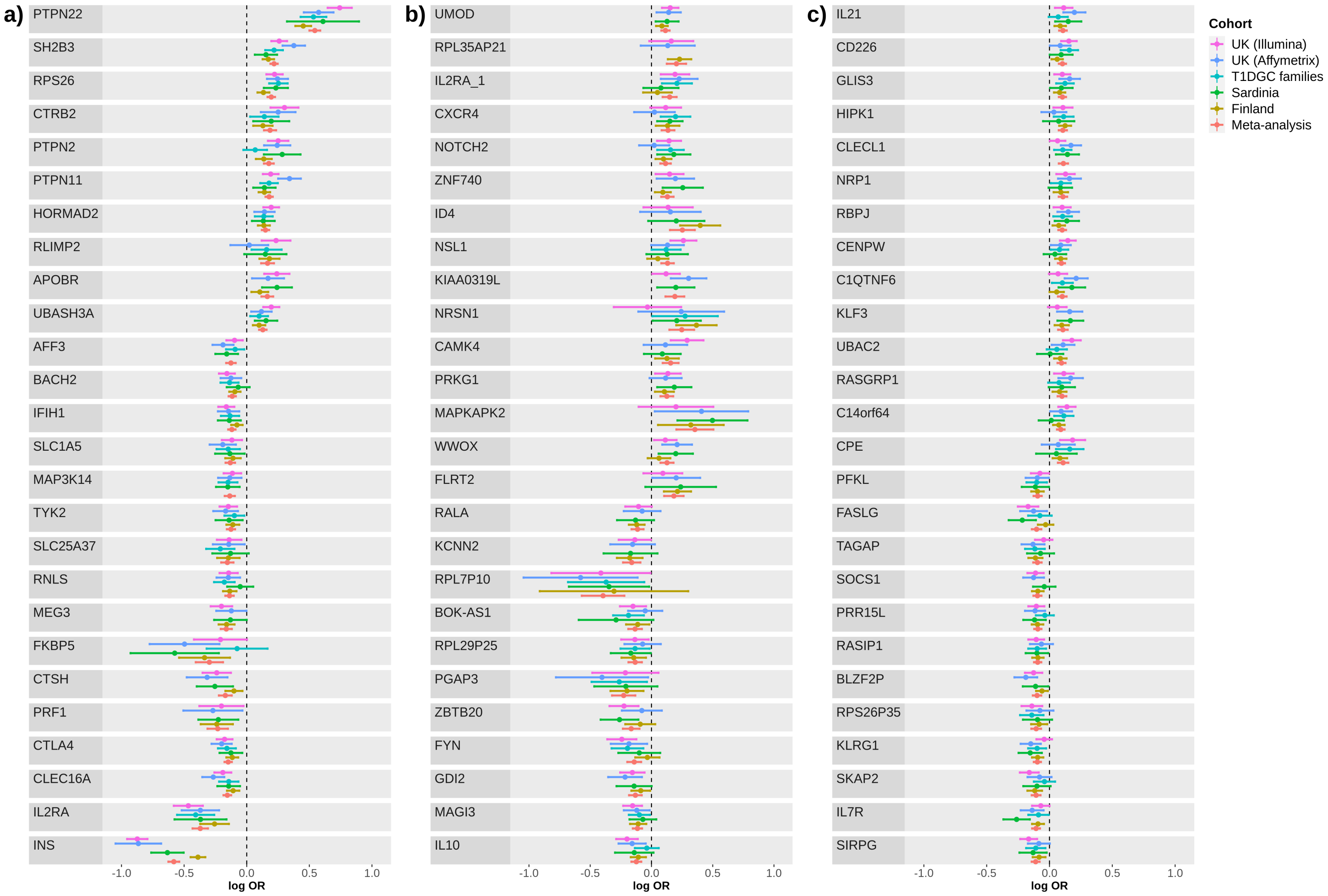

Figure S13

Type 1 diabetes test statistics from FinnGen (data release 6). For each variant, the GWAS Z-score using strictly defined T1D cases (y-axis) is plotted against its Z-sore using non-strictly defined T1D cases (x-axis). Non-strictly defined cases (code E4_DM1, n=7608) have an ICD-10 code of insulin-dependent diabetes mellitus, while strictly defined cases (code E4_DM1_STRICT, n=3,392) comprise the same samples after removal of those with ICD-10 codes of non-insulin-dependent diabetes mellitus. In the main text, we use non-strictly defined cases from FinnGen data release 4 (n=4,933).

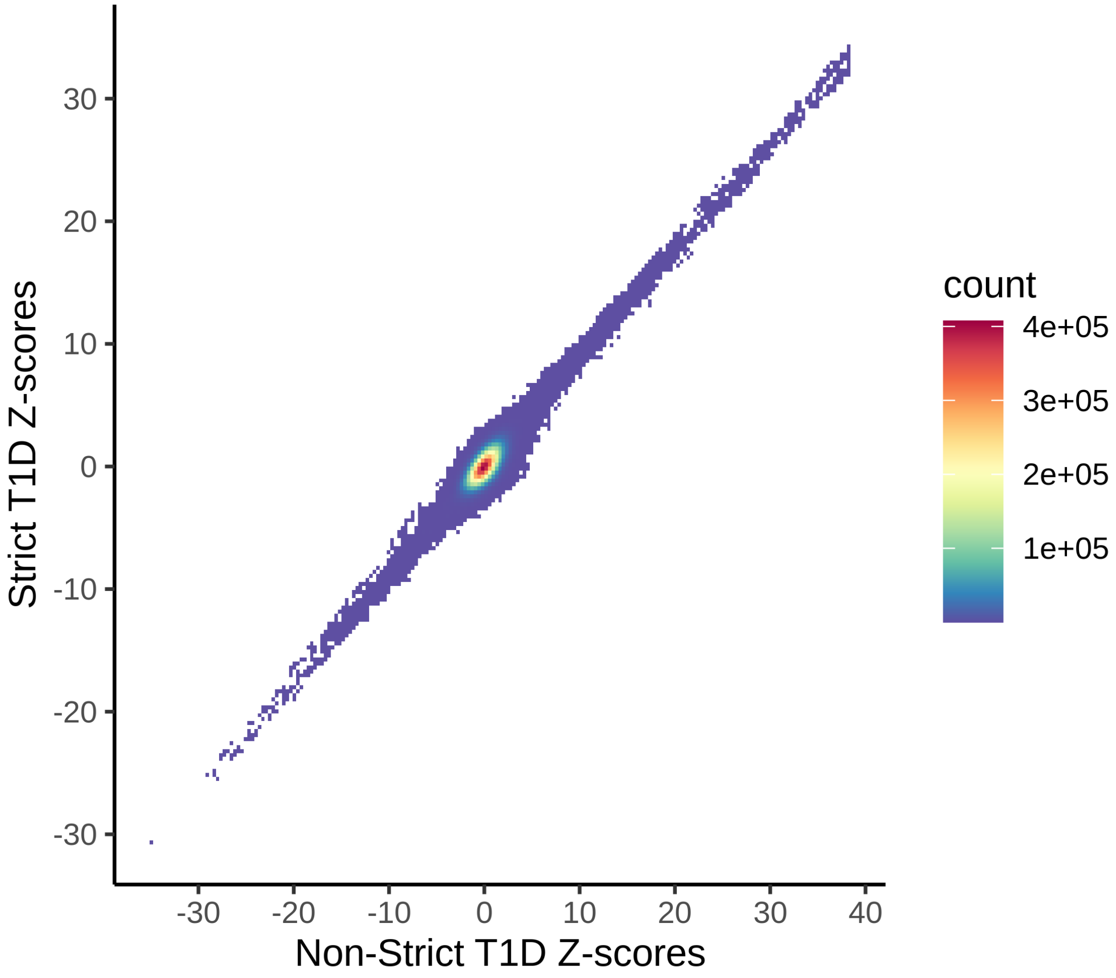

References

TURNER, S. D. 2018. qqman: an R package for visualizing GWAS results using Q-Q and manhattan plots. *Journal of Open Source Software,* 3(25), 731.
